# Transcriptomic profiling in human mesangial cells using patient-derived lupus autoantibodies identified miR-10a as a potential regulator of *IL8*

**DOI:** 10.1038/s41598-017-15160-8

**Published:** 2017-11-06

**Authors:** Pattarin Tangtanatakul, Boonyakiat Thammasate, Alain Jacquet, Rangsima Reantragoon, Trairak Pisitkun, Yingyos Avihingsanon, Asada Leelahavanichkul, Nattiya Hirankarn

**Affiliations:** 1 Medical Microbiology Interdisciplinary Program, Graduate School, Chulalongkorn University, Bangkok, 10330 Thailand; 2 Center of Excellence in Immunology and Immune-mediated Diseases, Department of Microbiology, Faculty of Medicine, Chulalongkorn University, Bangkok, 10330 Thailand; 3 Department of Medicine, Faculty of Medicine, Chulalongkorn University, Bangkok, 10330 Thailand; 4 Chulalongkorn University Systems Biology (CUSB), Faculty of Medicine, Chulalongkorn University, Bangkok, 10330 Thailand

## Abstract

Autoantibody-mediated inflammation directed at resident kidney cells mediates lupus nephritis (LN) pathogenesis. This study investigated the role of miRNA in human mesangial cells (HMCs) stimulated with auto anti-dsDNA immunoglobulin (Ig)G antibodies. HMCs were treated with antibodies purified from active LN patients or non-specific IgG controls in the presence of normal serum. Aberrant miRNA was screened using high throughput sequencing. Anti-dsDNA IgG up-regulated 103 miRNAs and down-regulated 30 miRNAs. The miRNAs regulated genes in the cell cycle, catabolic processes, regulation of transcription and apoptosis signalling. miR-10a was highly abundant in HMCs but was specifically downregulated upon anti-dsDNA IgG induction. Interestingly, the expression of miR-10a in kidney biopsies from class III and IV LN patients (n = 26) was downregulated compared with cadaveric donor kidneys (n = 6). Functional studies highlighted the downstream regulator of miR-10a in the chemokine signalling and cell proliferation or apoptosis pathways. Luciferase assay confirmed for the first time that *IL8* was a direct target of miR-10a in HMCs. In conclusion, anti-dsDNA IgG Ab down-regulated miR-10a expression in HMCs resulting in the induction of various target genes involved in HMC proliferation and chemokine expression.

## Introduction

Lupus nephritis (LN) is an immune-mediated kidney injury, which is a major complication in systemic lupus erythematosus (SLE)^1^. The incidence and prevalence of LN is about 40–70% among SLE patients depending on their ethnicity^2^. Despite advances in medicine, the standard therapeutic approach is still widely based on broad-spectrum immunosuppressants that cause various side effects including increased susceptibility to infectious agents and reproductive system failure^3^. A complete understanding of SLE pathogenesis is necessary to improve therapeutic approaches. Auto anti-dsDNA IgG antibodies are considered a hallmark of LN pathogenesis^4^ and the detection of these antibodies is associated with the development of proliferative LN disease^5,6^. The presence of anti-dsDNA IgG antibodies-immune complexes within glomeruli or cross-reactive anti-dsDNA antibodies to residential kidney cells are a key contributor to driving inflammation in the kidney^7,8^.

Mesangial cells (MCs) are specialised pericytes located in the glomerular tuft^9,10^, which support capillary constriction and dilation, and maintain the glomerular structure by generating a mesangial matrix^11^. A previous study showed that mesangial cells amplified inflammation in the kidney by acting as antigen presenting cells and inflammatory cytokine producing cells^12^. A cDNA microarray of mouse mesangial cells stimulated with anti-dsDNA IgG antibodies resulted in the up-regulation of genes in the cytokine and chemokine signalling pathways^13^. A study of the regulatory mechanisms that control these responses is required and might identify new therapeutic targets.

MicroRNAs function as endogenous epigenetic regulators, which fine-tune gene expression through direct binding with the 3ʹ untranslated regions (UTR) of target mRNA genes resulting in mRNA degradation or translation inhibition^14^. Atypical miRNA expressions were reported in many disease conditions including LN^15,16^. A study of miRNA expression levels in kidney biopsies from LN patients revealed several miRNAs that were either upregulated or downregulated compared with healthy controls^17^. Although evidence has illustrated abnormal miRNAs in LN, which microRNAs are related to anti-dsDNA IgG antibody stimulation in specific resident kidney cells have not been characterised.

The aberrant function of human MCs (HMCs) by anti-dsDNA IgG stimulation was considered an initial step of kidney injury in LN pathogenesis^18^. Studying the regulatory mechanisms during this induction might help understand LN pathogenesis. The objective of this study was to identify aberrant miRNAs and their functional roles in HMCs upon stimulation with anti-dsDNA antibodies, mimicking the initial physiological conditions in LN pathogenesis. In this study, we were focusing on miR-10a due to its potential role to regulate different phenotypes of HMCs. The miR-10a was significantly downregulated in HMCs in the presence of anti-dsDNA IgG as well as in kidney biopsies of LN patients. Its deregulation led to the overexpression of various target genes involved in LN pathogenesis including those involved in mesangial cell proliferation and inflammation. The target genes of miR-10a in HMC were investigated. Furthermore, the *IL8* gene was identified as a new target of miR-10a in mesangial cells.

## Results

### HMCs respond to anti-dsDNA antibodies

**Figure 1:**
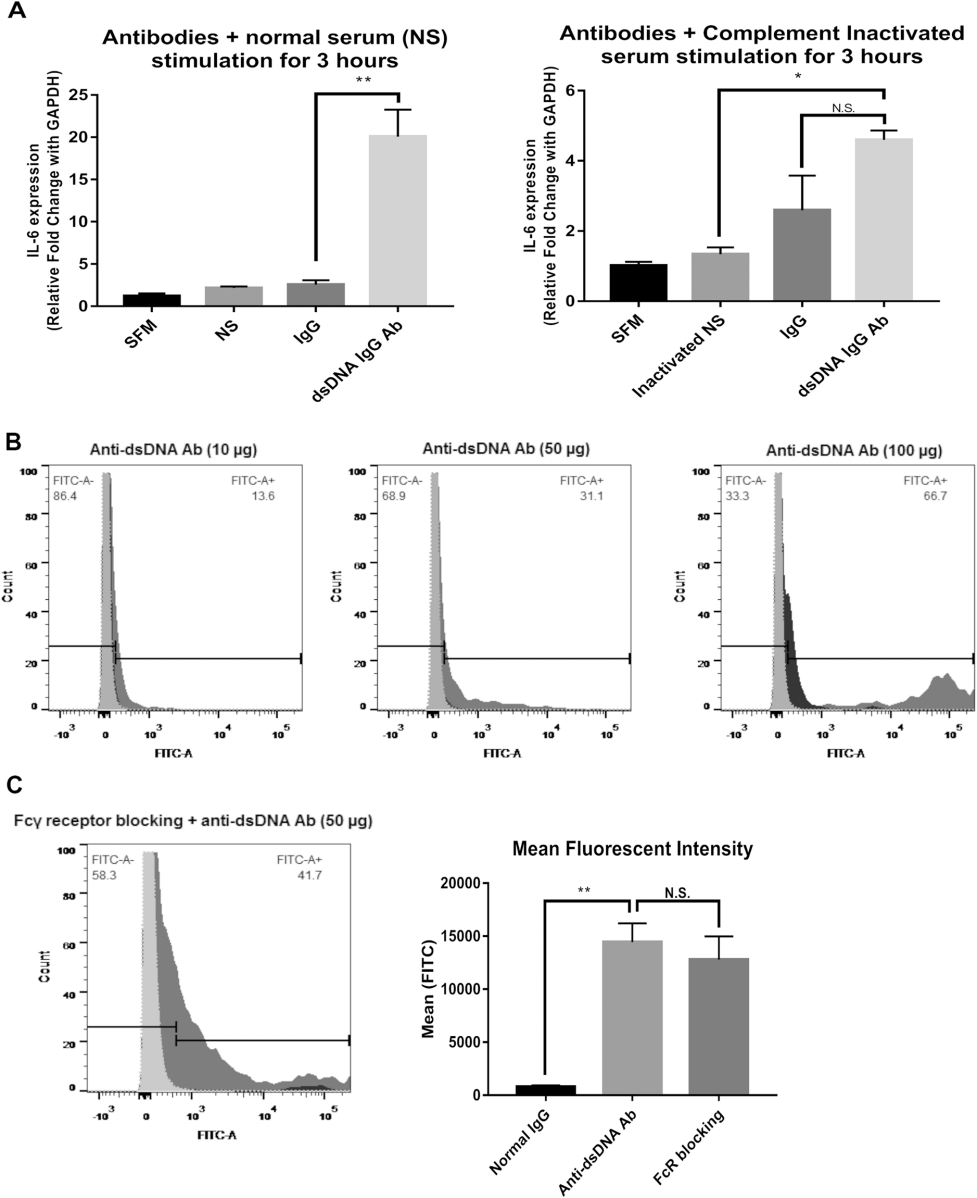
Anti-dsDNA antibodies bind to human mesangial cells and up-regulate *IL6*. (**A**) *IL6* expression in human mesangial cells (HMCs) stimulated with non-specific IgG and anti-dsDNA antibodies (10 µg/mL) in the presence of normal serum (NS) (left) or heat-inactivated serum (56 °C, 30 minutes) (right). (**B**) Flow-cytometry histograms of HMCs stained with anti-dsDNA antibodies (dark grey), non-specific IgG (black), and secondary antibodies alone (light grey) at different concentrations (10, 50, 100 µg/mL). (**C**) (Left) Flow cytometry histogram of HMCs pre-incubated with Fcγ-receptor blocking reagent and stained as described above at a concentration of 50 µg/mL. (Right) Mean fluorescent intensity (MFI) of HMCs stained with anti-dsDNA IgG antibodies (50 µg/mL) with or without pre-incubation with Fcγ-receptor blocking reagents compared with non-specific IgG controls. qPCR data shows a minimum of 3 biological replicates analysed independently. Graphs show data expressed as the mean ± standard error of the mean. *P < 0.05, **P < 0.01, and ***P < 0.001 versus serum free medium or normal IgG, respectively (unpaired *t-*test).

A previous report showed that anti-dsDNA IgG antibodies upregulated interleukin 6 (*IL6*) expression in HMCs^19^. Because we are interested in autoantibody-mediated resident kidney cell induced inflammation, we used *IL6* expression as a marker for HMC responses to autoantibodies in this study. Purified anti-dsDNA IgG antibodies from active LN patients’ sera or purified IgG antibodies from healthy controls (10 µg/mL) in the presence of normal serum were treated with HMCs for 3 hours according to conditions determined in preliminary experiments (Fig. S1). As expected, anti-dsDNA IgG antibodies upregulated *IL6* gene expression significantly compared with IgG antibodies from healthy controls (*p-*value < 0.001) (Fig. 1A). In contrast, heat inactivated serum (complement deactivation) dramatically reduced *IL6* expression, although *IL6* was still expressed and was not significantly different from IgG controls (Fig. 1A). These results suggested that complement activation was necessary for *IL6* induction through autoantibody stimulation. Antibody binding was also verified by flow cytometry. Suspended HMCs were stimulated with anti-dsDNA IgG antibodies or non-specific IgG followed by anti-human IgG Fc region antibodies conjugated with FITC. The numbers of FITC positive cells were increased by anti-dsDNA IgG antibody staining compared with IgG control or in the absence of any primary IgG antibodies (Fig. 1B). The binding was increased dose-dependently. When Fcγ receptor blocking reagents were used, there was no inhibition of antibody binding to HMC membranes (anti-dsDNA Ab 20.5% *vs* IgG 2.22% and anti-dsDNA Ab 20.5% *vs* secondary Ab 2.19%, *p*-value < 0.05) (Fig. 1C). This suggested that antibody binding was not via Fc portion-FcγR ligation on HMCs. We used this condition for further experiments.

### The miRNA profile in HMCs treated with anti-dsDNA IgG antibodies

**Figure 2:**
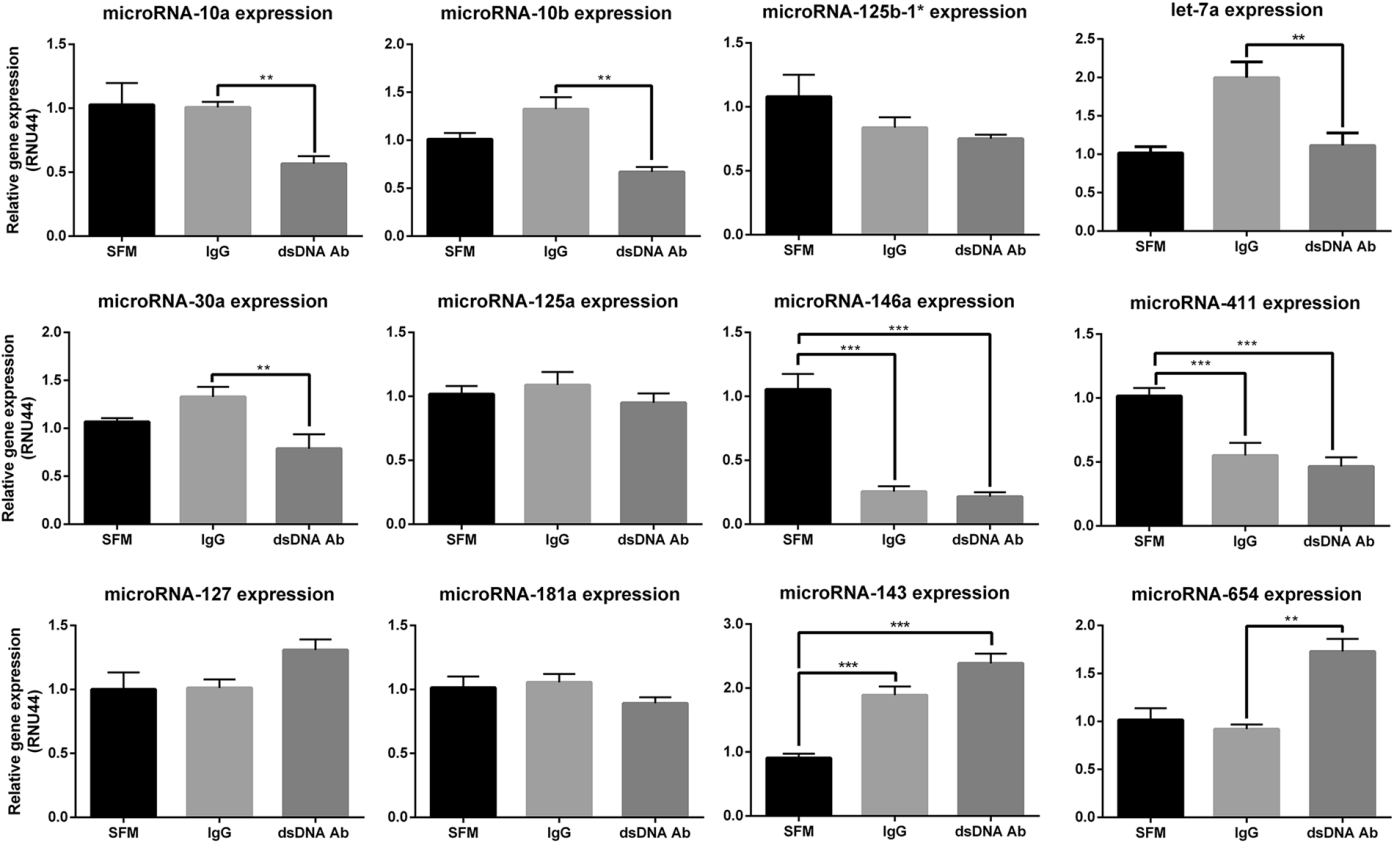
Candidate miRNAs expression in HMCs treated with anti-dsDNA IgG antibodies. Graphs show miRNA expression in HMCs treated with anti-dsDNA IgG antibodies (10 µg/mL) for 3 hours using stem-loop Real-Time PCR and qPCR. Data are normalised with RNU44 expression and compared with untreated conditions (SFM). Data are analysed using unpaired Student’s *t-*test and expressed as the mean ± SEM. *P < 0.05, **P < 0.01 and ***P < 0.001 versus normal healthy IgG controls.

To screen for altered miRNA expression in HMCs stimulated with anti-dsDNA IgG antibodies or IgG antibodies from healthy controls, miRNA sequencing was conducted from one pooled sample of miRNA per group (miRNA extracted from pooled anti-dsDNA IgG antibodies VS pooled control IgG stimulated HMCs). Thus, it should be noted that we mainly used miRNA sequencing as screening tool to select candidate miRNA to validate. List of abundant differentially expressed miRNAs based on fold change are shown in Table 1 (full list is shown in Supplementary Table 1). Candidate miRNAs with fold-changes ≤ 0.8 or > 1.5 (Table 1) were selected for validation by qPCR based on their relevant function (Table 2). The qPCR results confirmed that miR-10a/b, miR-30a and let-7a were significantly downregulated, while miR-654 was significantly upregulated in HMCs upon anti-dsDNA IgG antibody stimulation compared to IgG control. The miR-143 was significantly upregulated in HMCs in both conditions, while miR-146a and miR-411 were significantly downregulated in both conditions. The expressions of other miRNAs including miR-181a, miR-125a, miR-125b-1*, and miR-127 did not differ significantly between the two conditions (Fig. 2A).

**Table 1:** The list of microRNAs with differential ratio between HMCs treated with anti-dsDNA IgG antibodies and IgG from healthy controls (FC ≤ 0.8 and FC ≥ 1.5)^c^.

miRNA	IgG (RPM^a^)	Anti-dsDNA Ab (RPM^a^)	Ratio (dsDNA Ab/IgG)
**hsa-miR-10b-5p**	**145632**.**32**	**65573**.**76**	**0**.**45**
**hsa-miR-10a-5p**	**75054**.**25**	**39070**.**54**	**0**.**52**
**hsa-miR-125b-1** ^b^	**2558**.**54**	**1368**.**74**	**0**.**53**
hsa-miR-424–5p	378.44	239.09	0.63
hsa-miR-500a-5p	276.98	184.91	0.67
**hsa-let-7a-5p** ^b^	**110934**.**01**	**75416**.**84**	**0**.**68**
hsa-miR-146b-5p	1069.9	784.41	0.73
hsa-miR-23a-5p	1845.1	1394.8	0.76
hsa-miR-196a-5p	1180.28	2166.54	0.76
hsa-miR-196b-5p	624.76	479.95	0.77
hsa-miR-335–5p	1983.1	1532.89	0.77
**hsa-miR-30a-5p**	**12534**.**05**	**9851**.**33**	**0**.**79**
**hsa-miR-125a-5p**	**8162**.**27**	**6471**.**66**	**0**.**79**
hsa-miR-186–5p	3217.15	2557.87	0.8
hsa-miR-31–5p	5632.58	8459.06	1.5
hsa-miR-218–5p	143.43	217.75	1.52
hsa-miR-1307–3p	188.23	289.72	1.54
hsa-miR-27b-3p	17248.23	26980.37	1.56
hsa-miR-181a-2–3p	485.25	758.32	1.56
hsa-miR-214–5p	147.62	230.5	1.56
hsa-miR-192–5p	375.36	597.06	1.59
**hsa-miR-127–3p**	**28617**.**11**	**45906**.**19**	**1**.**6**
hsa-miR-130b-3p	1013.6	1635.39	1.61
hsa-miR-615–3p	479.58	773.2	1.61
**hsa-miR-181a-5p**	**10733**.**93**	**17392**.**18**	**1**.**62**
hsa-miR-24–3p	1145.59	1855.56	1.62
hsa-miR-589–5p	257.11	416.36	1.62
hsa-miR-106b-5p	127.11	205.6	1.62
hsa-miR-25–3p	1993.76	3250.42	1.63
**hsa-miR-411–5p**	**4387**.**85**	**7137**.**99**	**1**.**63**
hsa-miR-487b-3p	232.52	380.53	1.64
**hsa-miR-143–3p**	**31655**.**22**	**51789**.**63**	**1**.**64**
hsa-miR-199a	6287.35	10372.02	1.65
hsa-miR-1185–1–3p	138.26	228.98	1.66
hsa-miR-134–5p	431.9	720.06	1.67
hsa-miR-210–3p	211.32	358.36	1.7
hsa-miR-889–3p	619.63	1061.1	1.71
hsa-miR-345–5p	514.41	907.13	1.76
hsa-miR-151a-5p	7084.05	12817.96	1.79
hsa-miR-323a-3p	134.58	241.74	1.8
hsa-miR-410–3p	2002.12	3635.51	1.82
hsa-miR-584–5p	102.03	186.77	1.83
hsa-miR-27a-3p	4591.61	8521.01	1.86
hsa-miR-431–5p	390.89	770.77	1.97
hsa-miR-1307–5p	700.35	1456.81	2.08
**hsa-miR-654–3p**	**3971**.**19**	**8503**.**09**	**2**.**14**

^a^RPM = reads per million.

^b^Note: there is an inconsistent between two algorithms.

^c^Bold letters were miRNAs selected for validation in this study.

**Table 2:** Details of microRNAs that were selected for validation.

miRNA	Expression ratio (anti-dsDNA/IgG)	Findings from previous reports	References
miR-10b	0.45	- Upregulated in breast cancer and promote cell metastasis	64
miR-10a	0.52	- Downregulated in kidney biopsy from ischemic-reperfusion and STZ-induced renal injury mouse model - Upregulated in CD19+ cells from asymptomatic SLE patients compared with healthy controls	39,65
miR-125b-1^a^	0.53	- Anti-apoptotic miRNA in acute myeloid leukaemia	66
let-7a^a^	0.68	- Upregulated in mouse mesangial cells enhancing *IL6* expression - Regulated TNFAIP3-NF-kB signalling which involved in LN pathogenesis	50,67,68
miR-30a	0.79	- Downregulated in B-cells isolated from SLE patients promoted B-cell hyperactivity - Upregulated in LN renal biopsy	69,70
miR-125a	0.79	- Downregulated in T-lymphocytes from SLE patients regulating RANTES levels - Regulate macrophages differentiation and inflammatory cytokine production through NF-kB	71,72
miR-146a	1.26	- Downregulated in THP-1 cells and WBC from SLE patients by IFN type I stimulation - Upregulated in kidney biopsy from LN patients especially in the glomerular part - Downregulated in serum from SLE patients but up-regulated in urine from SLE patients	73–75
miR-411	1.55	- Upregulated in lung cancer cells promoting cell proliferation by target FOXO genes - Upregulated in hepatocellular carcinoma enhanced cell proliferation by target ITCG genes	76,77
miR-127	1.62	- Upregulated in purified splenocytes from MRL/lpr, B6-lpr and NZB/W F_1_ lupus nephritis mice	78
miR-181a	1.62	- Upregulated in serum from SLE patients - Upregulated in human hepatocyte cell line in response to TGF-beta inducing EMT	79,80
miR-143	1.64	- Upregulated in CD4 + T-lymphocytes in asymptomatic SLE patients compared to healthy controls	65
miR-654	2.14	- Upregulated in the EBV-transformed B cell line from European American SLE patients - Downregulated in LN renal biopsy	17,81

LN, lupus nephritis; SLE, systemic lupus erythematosus; STZ, streptozotocin; IFN, interferon; EMT, epithelial-mesenchymal transition; TGF, transforming growth factor.

^a^There is an inconsistent prediction between two algorithm miRDeep2 and sRNAbench.

### Transcriptomic profiling of HMCs treated with anti-dsDNA antibodies and integration with validated and/or predicted targets of candidate miRNAs

**Figure 3:**
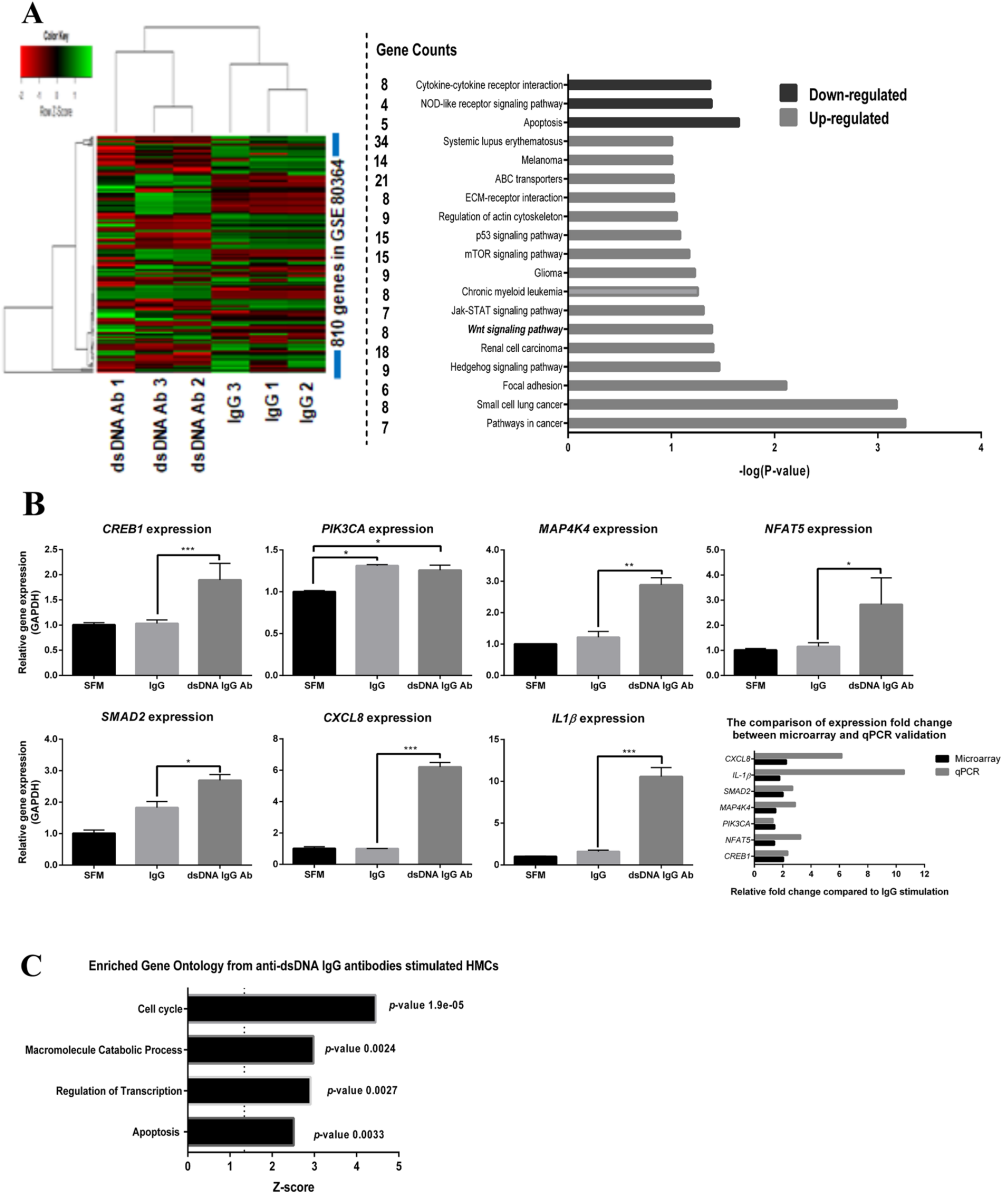
Transcriptomics profiling of HMCs treated with anti-dsDNA IgG antibodies. (**A**) (Left) Heat-map shows the expression correlation between group of samples and significantly upregulated or downregulated genes after anti-dsDNA IgG antibody induction (fold-change > 1.2 or < 1.2). (Right) Genes that were upregulated or downregulated significantly during anti-dsDNA IgG antibody stimulation are clustered based on their involvement in the same pathway (KEGG-pathway analysis). (**B**) The graphs show qPCR validation from selected candidate mRNAs according to microarray result and comparison of fold change expression in microarray and qPCR. The expressions were normalized using GAPDH. (**C**) The graph demonstrates the microarray results using functional annotation clustering and enrichment scores with adjusted *P*-value in the DAVID bioinformatics resources version 6.7 (https://david.ncifcrf.gov/tools.jsp). Microarray data were analysed using lumi R-bioconductor packages. Multiple corrections (FDR) were performed by the Bonferroni-Hochberg method. The *p*-values < 0.05 were considered significant. qPCR data are expressed as mean ± SEM of a minimum of three biological samples from three independent experiments. *P < 0.05, **P < 0.01, and ***P < 0.001 versus scramble controls.

To characterise the HMC response upon auto anti-dsDNA IgG stimulation, we performed transcriptomic profiling using total RNA from previous experiments. Our results revealed that 810 genes were significantly altered by auto anti-dsDNA IgG antibody induction (GSE80364). The genes were clustered by KEGG pathway analysis, which showed that several genes in the extracellular matrix, the WNT, JAK-STAT, mTOR, p53, and SLE-signalling pathways were upregulated (FC > 1.2, FDR adj. *p*-value < 0.05), whereas cell apoptosis, NOD-like receptor signalling and cytokine-cytokine receptor interaction pathways were downregulated (FC < 0.5, FDR adj. *p*-value < 0.05) (Fig. 3A). Some upregulated genes including transforming growth factor-beta SMAD dependent signalling (*SMAD2*), transcription factor CREB and its extracellular signals (*CREB1*), Ca++/Calmodulin-dependent protein kinase activation (*PIK3CA*) and MAPK-pathways (*MAP4K4*), nuclear factor of activated T-cell 5 (*NFAT5*), interleukin-1 beta (*IL1B*) and C-X-C motif chemokine ligand 8 (*CXCL8)* or interleukin 8 (*IL8*) were validated by qPCR (Fig. 3B). Most of them were significantly upregulated in anti-dsDNA IgG antibody treated HMCs compared to IgG from healthy controls, except *PIK3CA*, that was upregulated in both conditions. Gene functional annotation clustering showed that anti-dsDNA IgG antibodies mainly affected genes involved with the cell cycle, catabolic processes and regulation of transcription and apoptosis (Fig. 3C). This help suggests that anti-dsDNA IgG antibodies are important in the increase of HMC proliferation and apoptosis during the early phase of responses. Moreover, these antibodies might induce transcription factors that affect downstream signalling pathways, especially those involved in cytokine and chemokine production.

**Figure 4:**
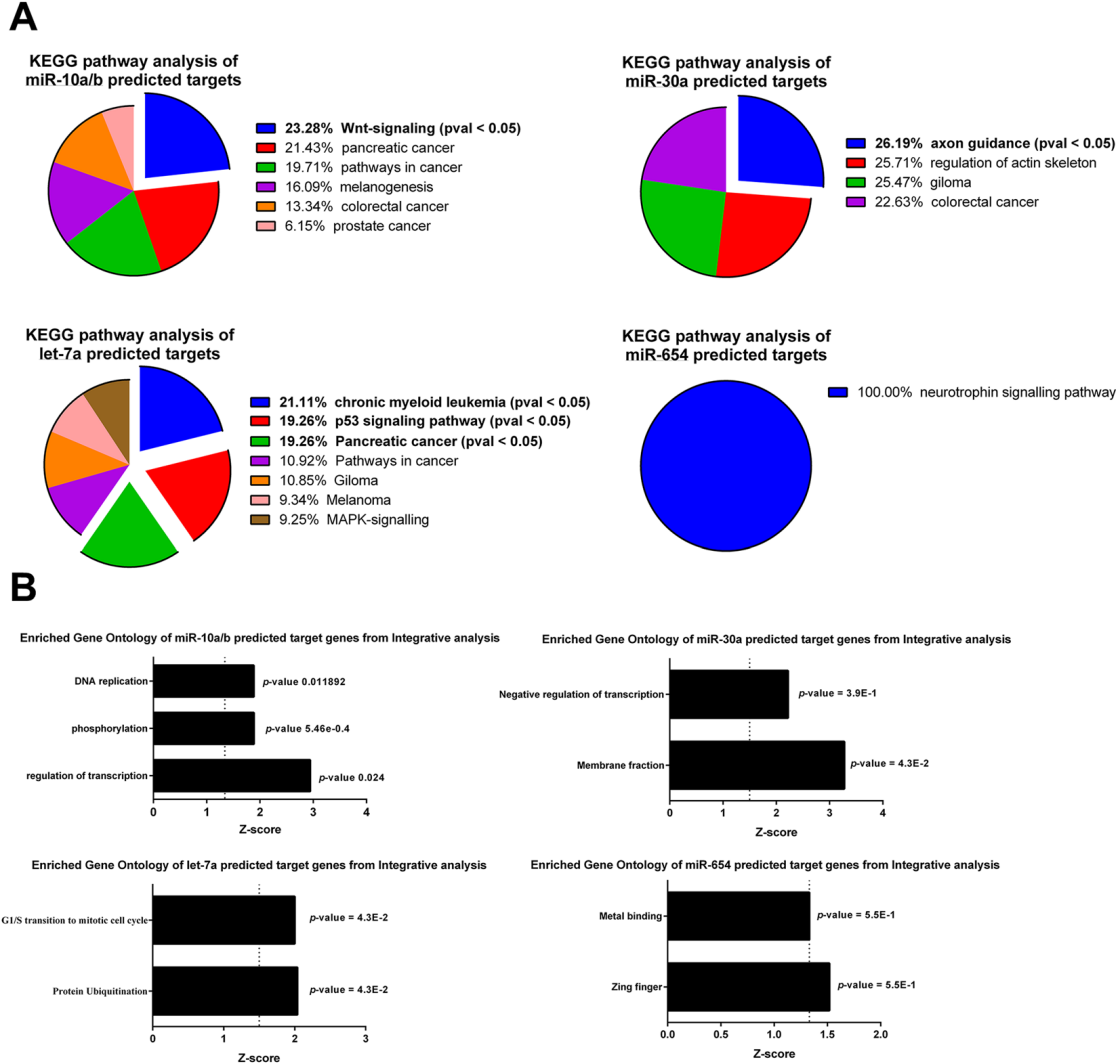
KEGG pathway analysis and functional annotation clustering of predicted/validated target genes of candidate miRNA that differentially expressed in activated HMCs. (**A**) Pie charts show KEGG pathway analysis from integrative analysis between predicted/validated target genes of miR-10a/b, miR-30a, miR-654 and let-7a with anti-dsDNA IgG antibodies stimulation transcriptomic. (**B**) The graph represents gene functional annotation clustering of integrative analysis between candidate miRNA (miR-10a/b, miR-30a, miR-654 and let-7a) and microarray mRNA data using ingenuity pathway analysis (IPA, Qiagen) with negative correlation and high-to-medium confidence target prediction. The prediction criteria are based on TargetScan.

We performed integrative analysis between mRNA profile with putative target genes of miR-10a, miR-10b (noted that they have similar seed region and target genes), let-7a, miR-30a and miR-654. The upregulated miR-654 was associated with 99 of its target genes that were downregulated in activated HMCs (Supplementary Table 2). The KEGG analysis pathway of miR-654 putative target genes that were downregulated in HMCs showed no significant relevant pathway (Fig. 4A and B). The downregulated miR-30a associated with upregulated 428 target genes in HMCs treated with anti-dsDNA IgG antibodies (Supplementary Table 3). The only significant pathway of miR-30a-regulated target genes was in axon guidance pathway (Fig. 4A) and function in membrane fraction (Fig. 4B). The downregulated let-7a was predicted to upregulate 383 target genes in activated HMCs (Supplementary Table 4). The significant pathways were mostly related to cancer e.g., chronic myeloid leukaemia, p53-signalling and pancreatic cancer (Fig. 4A). Functional annotation clustering revealed that let-7a might play a role in protein ubiquitination and G1/S transition to mitotic phase (Fig. 4B). Lastly, we identified 81 predicted miR-10a/b target genes that were downregulated in HMCs treated with anti-dsDNA IgG antibodies (Supplementary Table 5). The functional annotation clustering of these genes was significantly classified as protein phosphorylation, regulation of transcription and DNA replication (Fig. 4B). Interestingly, the KEGG-pathway analysis showed that miR-10a/b target genes in HMCs were present significantly in the WNT-signalling, similarly to microarray results of HMCs treated with anti-dsDNA IgG autoantibodies (Fig. 4A). Since the upregulation of genes in the WNT signalling pathway usually results in increased cell proliferation and differentiation^20^, we hypothesized that miR-10a/b might have an important role in HMCs activated by anti-dsDNA IgG antibodies. In addition, miR-10a and miR-10b were the most abundant microRNA present in HMCs. In this study, we focused mainly on miR-10a. Therefore, we further validate miR-10a expression in kidney biopsy and characterized target genes of miR-10a in HMCs.

### miR-10a is downregulated in kidney biopsies isolated from LN patients and promote HMC proliferations *in vitro*

**Figure 5:**
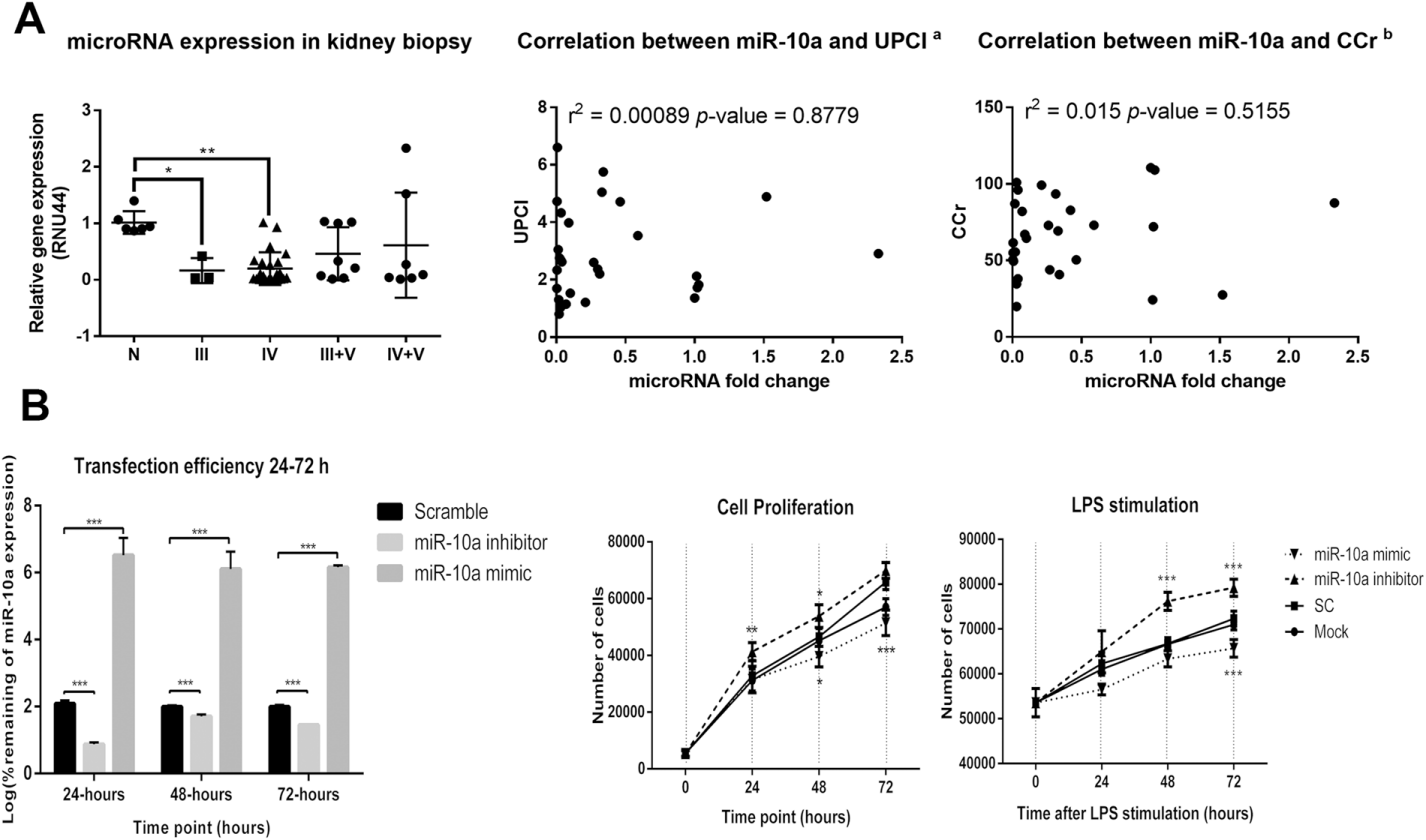
The miR-10a expression in kidney biopsy from LN patients and the effect of miR-10a inhibitor or miR-10a mimic transfection in human mesangial cell proliferation. (**A**) (Left) miR-10a expression in kidney biopsies from lupus nephritis (LN) patients grouped by histology class including class III (n = 3), IV (n = 23), III + V (n = 8), and IV + V (n = 8) compared with cadaveric donor kidney biopsies (n = 6). Expressions are normalised to RNU44 expression. Graphs show the correlation between miR-10a expression and disease severity determined by urine protein creatinine index (UPCI)^a^ (Middle) or creatinine clearance (CCr)^b^ (Right). (^a^) There are missing data for UPCI and miR-10a expression correlation. We conducted this correlation only from 29 samples. (^b^) The correlation was performed using data from 29 samples. (**B**) (Left) The graph shows the log percent remaining of miR-10a expression after miR-10a inhibitor or miR-10a mimic transfection compared with scramble negative controls at different time points (24, 48 and 72 hours). (Middle) Cell proliferation assay using human mesangial cells (HMCs) transfected with miR-10a inhibitor (▲), miR-10a mimic (▼), scramble negative control (■) and lipofectamine or mock control (●) at different time points (0, 24, 48 and 72 hours). (Right) The cells were stimulated with LPS before proliferation assay. The cell number was compared between samples at the same time points using an unpaired Student’s *t-*test. Data were analysed using the Mann-Whitney *U-*test in tissue samples and are expressed as the mean ± SEM. (*) denotes P < 0.05, and (**) denotes P < 0.01 versus cadaveric donor kidney or scramble controls.

In the likely manner with qPCR, the expression of miR-10a in kidney biopsies from class III and IV LN patients (n = 26) were downregulated compared with cadaveric donor kidneys (n = 6) (Fig. 5A). However, the miR-10a expression was not significantly different in LN patients with mixed classes (class III + V (n = 8) or class IV + V (n = 8)) compared to control. There was no significant correlation between miR-10a expression and urine protein (UPCI) or creatinine clearances (CCr) (Fig. 5A).

The previous experiment showed downregulated miR-10a promoted myeloid cells hyperproliferation^21^. Furthermore, HMC proliferation is a key feature in type I–IV LN histology^22^. We therefore hypothesised that the downregulation of miR-10a might result in HMC over expansion. HMCs were transiently transfected with a miR-10a inhibitor and a miR-10a mimic for 24, 48 and 72 hours. At 24 hours after mimic transfection, the miR-10a overexpression was greater than 100% but was less than 5% after miR-10a inhibitor transfection compared to scramble controls (Fig. 5B). It should be noted that the miR-10a expression increased to 30–50% at 48 and 72 hours after miR-10a inhibitor transfection whereas the miR-10a mimic transfection showed a consistent overexpression of miR-10a from 24 to 72 hours. This was likely resulted from decreased efficiency of transient inhibitor transfection rather than the induction of miR-10a since the level of miR-10a was stable over time in the scramble controls (data not shown). Cell proliferation was investigated using an MTS assay (OD490). As expected, the miR-10a inhibitor transfection increased HMC proliferation significantly (Fig. 5B). On the other hand, the miR-10a mimic transfection decreased cell proliferation compared to scramble negative controls, indicating miR-10a responses controlled HMC proliferation. In order to examine the role of miR-10a under stimulatory condition, LPS was used to stimulate HMCs. Upon LPS stimulation, the effects of miR-10a inhibitor and miR-10a overexpression was enhanced (Fig. 5B).

### Validation of miR-10a target genes in HMCs

**Figure 6:**
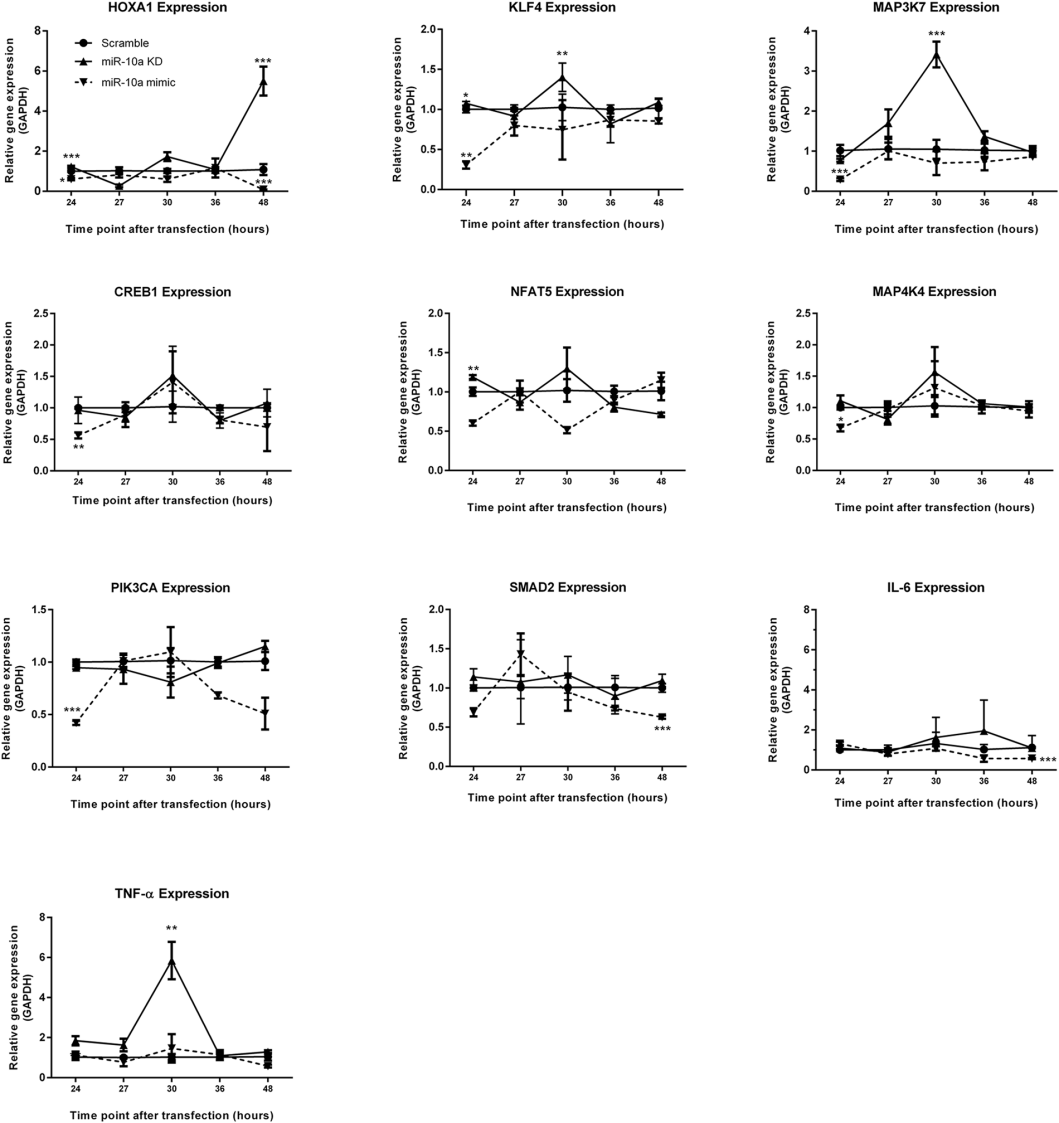
The kinetic expression of potential miR-10a target genes in miR-10a transient knockdown or miR-10a overexpressing human mesangial cells. Human mesangial cells (HMCs) were transfected with miR-10a inhibitor (▲), miR-10a mimic (▼) or scramble negative controls (●) using Lipofectamine RNAiMAX (siRNA concentration = 5 pmol). The total RNA including miRNA was collected at 24, 27, 30, 36 and 48 hours after transfection. Fold changes were compared between samples at the same time points using the unpair *t-*test. Data are shown as mean ± S.E.M. of a minimum of three biological replicates from three independent experiments. *P < 0.05, **P < 0.01, and ***P < 0.001 versus scramble controls.

We selected previous target genes of miR-10a for validation by real-time PCR. Among known validated targets Homeobox A1 (*HOXA1* ^23^), Kruppel-like factor 4 (*KLF4* ^24^) and mitogen-activated protein kinase kinase kinase 7 (*MAP3K7* ^25^) in other cell types, *HOXA1* was slightly upregulated after 24 hours transfection. Its expression was highly upregulated at 48 hours while *KLF4* and *MAP3K7* were significantly upregulated at 30 hours after miR-10a inhibitor transfection (Fig. 6). miR-10a mimic transfection exhibited the opposite result for *HOXA1* expression. *KLF4* and *MAP3K7* were markedly downregulated at 24 hours and then returned to normal levels at 27 hours. Based on integrative analysis, miR-10a was predicted to control *SMAD2*, *CREB1*, *PIK3CA*, *MAP4K4*, and *NFAT5* which were crucial for generating extracellular matrix, responses to stress activation, and inflammatory responses (Supplementary Table 5). These genes contain a conserved region for the miR-10a binding site in their 3ʹUTR as determined by TargetScan version 7 (released August 2015) with a high confidence score. Surprisingly, miR-10a knockdown HMCs slightly upregulated *NFAT5* at 24 hours whereas other genes (*CREB1*, *MAP4K4*, *PIK3CA*, and *SMAD2)* were not significantly upregulated. In contrast, gene expression in miR-10a mimic transfectants showed significant downregulation of most genes, of which included *CREB1*, *PIK3CA*, and *MAP4K4* at 24 hours after transfection (Fig. 6), whereas *NFAT5* and *SMAD2* showed a non-significant downregulation. *SMAD2* was significantly downregulated at 48 hours after transfection (Fig. 6). These results present the spatial and temporal regulation of miR-10a. We next tested whether miR-10a knockdown might affect pro-inflammatory cytokine gene expression. Unexpectedly, *TNFA* was significantly upregulated in miR-10a knockdown HMCs, but there was no effect on *IL6* and *IL1B* expression (Fig. 6). However, the expression of *IL6* was significantly downregulated after miR-10a mimic transfection at 48 hours. Taken together, these results suggest that the miR-10a downregulation is involved in HMC proliferation, possibly mediated by *HOXA1*, *KLF4* and *MAP3K7*. Moreover, the kinetic expression of miR-10a target genes shows dynamic miR-10a regulatory function.

### Transcriptomic profiling of miR-10a knockdown HMCs indicates *IL8* is a novel target

**Figure 7:**
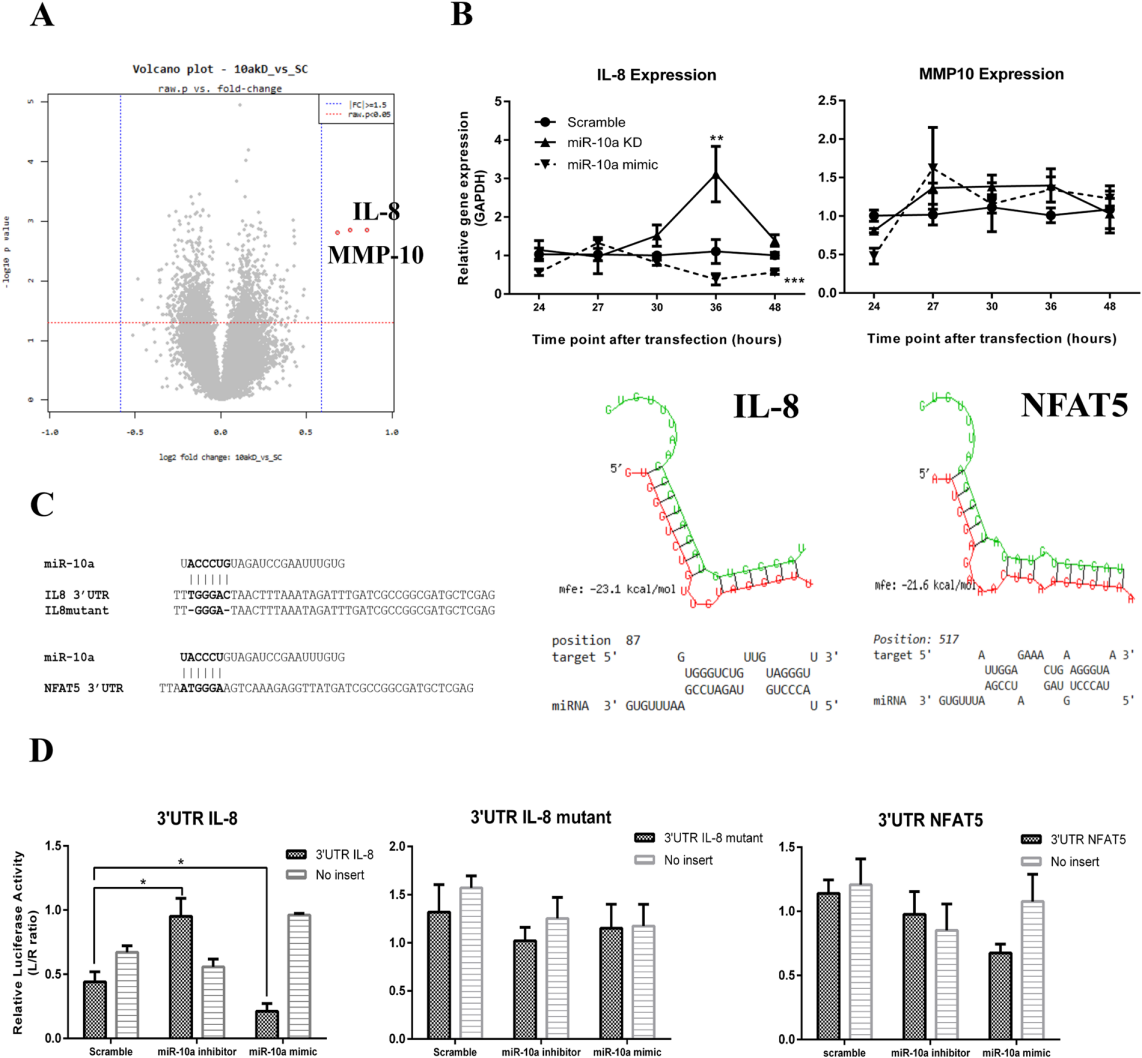
Transcriptomic profiling after miR-10a transient knockdown indicates *IL8* is a miR-10a target. (**A**) Volcano plot represents fold change differences between human mesangial cells (HMCs) transfected with miR-10a inhibitor compared with scramble negative control (raw P-value by an unpaired Student’s *t-*test). (**B**) The graphs show kinetic qPCR results of *IL8* and *MMP10* expression after miR-10a inhibitor (▲), miR-10a mimic (▼) or scramble control (●) transfection for 48 hours. (**C**) Bioinformatics prediction of miR-10a 3′UTR binding site in *IL8* and *NFAT5* with predictive binding energy and probability for actual binding. (**D**) The ratio of luciferase activity (relative luciferase ratio, Luciferase/Renilla) in miR-10a mimic co-transfection with a plasmid containing the *IL8* 3′UTR, *IL8* mutant 3′UTR and *NFAT5* 3′UTR compared to no insertion controls. Data are the mean ± S.E.M. of a minimum of three replicated experiments. Both qPCR and luciferase data were analysed using unpair *t-*test. *P < 0.05, **P < 0.01, and ***P < 0.001 versus the scramble group.

Further analysis to identify novel miR-10a target genes in HMCs was conducted using transcriptomic profiling in miR-10a inhibitor-transfected HMCs. Differentially expressed gene (DEG) analysis revealed 1264 genes were altered after miR-10a inhibitor transfection (raw *p*-value < 0.05). Unexpectedly, only two genes were upregulated over 1.5–fold. These included *IL8* and matrix metalloproteinase protein 10 (*MMP10*) (Fig. 7A). Using target predicted bioinformatics, we showed that miR-10a conserved seed regions bound complementarily to the 3′UTR of *IL8* (mfe = −23.1, mirSVR score = −0.1843, PhastCons score = 0.6004, Fig. 7C). However, there was no complementary region in the *MMP10* 3′UTR. Real-Time PCR results confirmed that only *IL8* was upregulated in miR-10a knockdown cells, whereas *MMP10* was slightly, but non-significantly upregulated (Fig. 7B). Consistently, miR-10a overexpression significantly downregulated *IL8* at 48 hours after transfection (Fig. 7B). To confirm that *IL8* mRNA is a direct target of miR-10a, we used a pmiR-Glo plasmid that contained the 3′UTR of *IL8* and co-transfected it with a miR-10a mimic, miR-10a inhibitor, or scramble negative control compared with the parent pmiR-Glo plasmid. Luciferase activity in pmiR-Glo containing the 3′UTR of *IL8* was dramatically decreased by miR-10a mimic transfection; in contrast, the activity was greatly increased by miR-10a inhibitor transfection (Fig. 7D). Furthermore, direct mutation at 3′UTR of *IL8* which is a seed region of miR-10a abrogated the effects observed from transfection of both miR-10a inhibitor and its mimic (Fig. 7D). This suggested that *IL8* is a novel direct target gene of miR-10a. We also conducted a luciferase assay for *NFAT5*, which is another potential novel target of miR-10a. As mentioned previously, *NFAT5* was slightly upregulated upon miR-10a inhibitor transfection. Moreover, the bioinformatics prediction showed that miR-10a bound directly to the 3′UTR of *NFAT5*. We therefore suspect that *NFAT5* is a direct target of miR-10a. Using a similar process, we found no reduction or implementation of luciferase activity upon either miR-10a mimic or miR-10a inhibitor transfection (Fig. 7D). This indicated that miR-10a directly controls *IL8*, but not *NFAT5*, expression.

## Discussion

Specific autoantibodies are associated with specific organ damage in systemic lupus erythematosus patients (SLE)^26^, especially auto anti-dsDNA IgG antibodies are associated with LN development^27^. The antibodies induce inflammation through immune complex deposition or their capability to recognise antigens or chromatin structures on endothelial cells, glomerular basement membranes and other resident kidney cells^28^. In this study, we demonstrated that polyclonal anti-dsDNA IgG antibodies purified from active LN patients bound to HMCs via the F’(ab) region, and that this binding capacity was increased in dose-dependent manner. HMCs are kidney pericyte cells that connect endothelial cells with the glomerular basement membrane. HMCs are involved in mesangial matrix production, eliminating immune complexes, antigen presentation and cytokine or chemokine production^29^. In our study, we demonstrated that the binding of auto anti-dsDNA IgG to HMCs in the presence of normal serum increased pro-inflammatory cytokine expression (*IL6*), which is an important mediator of the acute phase response usually secreted by antigen-presenting cells, such as macrophages. We found that incubating antibodies with complement-inactivated serum reduced *IL6* levels indicating that complement activation might be the main mechanism of pro-inflammatory cytokine production in HMCs. Consistent with a previous report, the terminal complexes of complements upregulated *IL6* expression in human smooth muscle cells^30^, suggesting the direct binding of autoantibodies to HMCs induces *IL6* expression through complement fixation.

Our study is the first report of miRNAs profiles that control acute inflammation responses in HMCs stimulated with anti-dsDNA IgG antibodies. Selecting from the list of miRNAs that we screened using miRNA sequencing, we were able to confirm by qPCR that several miRNA levels were altered after anti-dsDNA IgG antibody stimulation. The miR-654 was the only miRNA that was significantly upregulated by qPCR in this study. However, when we analysed putative target genes of miR-654 that were down regulated in HMCs treated with anti-dsDNA IgG antibodies, there was no significant pathways that was related to SLE pathogenesis. There were 4 miRNAs, miR-10a, miR-10b, miR-30a, miR-let-7a that were significantly downregulated by qPCR. In this study, we particularly focused entirely on miR-10a/b, which were the first 2 miRNAs that were significantly downregulated with the highest fold change difference. The miR-10a/b is encoded in the *HOX* cluster (*HOXB* and *HOXD*) of developmental genes^31^. Its expression is important in developmental pathways and is dysregulated in several types of cancers^32–35^. Although miR-10a and miR-10b showed similar expression patterns, miR-10b is predominantly shown to control cell metastasis and cell proliferation while miR-10a is a regulator of various inflammation processes^36^. Despite the interest in both miR-10a and miR10b, we mainly focused on characterization of the role of miR-10a involved in inflammation initiation due to auto anti-dsDNA IgG antibody stimulation. miR-10a is linked to pathogenesis of autoimmune diseases and inflammatory cytokine regulation. For example, a previous study in a mouse model of autoimmune inflammatory bowel disease showed that downregulated miR-10a in dendritic cells resulted in the upregulation of *IL-12/IL-23p40* ^37^. Studies in rheumatoid arthritis also indicated that TNF-α and IL-1β stimulation of fibroblast-like synoviocytes (FLSs) also downregulated miR-10a via an NF-κB dependent mechanism by inducing YY1 transcription factor^38^. Furthermore, an ischemic-reperfusion kidney injury mouse model showed marked decrease in miR-10a expression in the kidney, which was later identified as an acute kidney trauma marker^39^. Although miR-10a expression is not kidney-specific, it is predominantly expressed in mouse and human kidneys^40^. In this study, we also demonstrated miR-10a downregulation in kidney biopsies from LN patients when compared with levels in cadaveric kidney donors. Hence, we hypothesized that downregulated miR-10a by anti-dsDNA IgG antibodies might be an important contributing factor to LN pathogenesis.

Using integrative analysis between predicted target genes for specific microRNA and total RNA profiling from the same condition, the important significant pathway revealed from miR-10a target genes in HMCs is the WNT signalling pathway. This is interesting because it was consistent with a previous report in miR-10a knockout mice^24^. Since WNT signalling pathways are important in cell development, proliferation and differentiation^20^, we therefore hypothesize that miR-10a is crucial in the HMC propagation. Proliferation assays confirmed that miR-10a regulated HMC proliferation in both conditions (LPS stimulation or unstimulated). This suggested that anti-dsDNA IgG antibodies promote HMCs expansion by downregulating miR-10a. However, the mechanism by which miR-10a regulate cell proliferation is still unknown. Regarding to the miR-10a transfection, experimental validated target genes including *HOXA1*, *KLF4* and *MAP3K7* were studied. These genes are important in cell proliferation, differentiation, and pro-inflammatory cytokine production; thus, their upregulation might partly be involved in HMC proliferation. The experiments to examine a clear molecular mechanism in which miR-10a regulate HMCs proliferation might be useful for further study.

As for microarray results, we unexpectedly detect only two genes which were significantly upregulated after miR-10a inhibitor transfection for 24 hours. Those unexpected outcomes may be explained with several reasons. First, some miRNAs share their seed region and these predicted targets might be under the control of other miRNAs. Thus, we did not detect any elevated target gene expression by miR-10a knockdown; however, target gene expression was downregulated by miR-10a mimic transfection because these genes bear a miR-10a binding site. Second, miRNA regulation might not lead to target mRNA degradation, but rather repressed protein translation. Therefore, protein detection might be necessary to validate the miRNA target genes. One limitation of the study was that we did not analyse the corresponding proteins. Third, because miRNAs are expressed under specific conditions, the stimulation or stress environment chosen might be important when characterising miRNA targets. It should be noted that the miR-10a knockout mice did not show any abnormal phenotypes^24^; however; carcinogen administration in those mice can promote intestinal neoplasia. Therefore, environmental stress or specific stimuli might help identify more miRNA target genes. Unfortunately, we did not use any stimuli in our miR-10a knockdown microarray experiment.

An important finding in this study is that downregulation of miR-10a upregulates *IL8*. Luciferase assays confirmed *IL8* as a direct target for miR-10a in HMCs. This highlights the role of IL-8 in LN pathogenesis in the model of autoantibody-induced kidney injury. Recently, an *IL8* polymorphism has been reported to associate with severe LN in African Americans^41^. Additionally, immunohistochemistry found that IL-8 was dramatically increased in LN kidney compared to normal kidney^42^. IL-8 is a chemokine that recruits phagocytic cells to inflammatory sites^43^. *In vivo* evidence of immune complexes induce inflammation suggested that IL-8 is essential in type III hypersensitivity, which is a major characteristic of LN^44^. Neutrophils are a major source of renal endogenous nucleosome-induced immune complex formation^45,46^, and upregulation of *IL8* in HMCs might recruit neutrophils into inflammatory sites, thus, further amplifying inflammation. Neutrophils undergo “NETosis” cell death programming promoting chromatin accumulation in the glomerulus^47,48^. Moreover, IL-8 might induce the expression of cell adhesion molecules in neighbouring cells including endothelial and epithelial cells^49^. Interestingly, neutrophils producing IL-8 is required for immune complex deposition trigging inflammation^44^. However, additional *in vivo* experiments in LN mouse model are necessary to confirm these results.

Apart from miR-10a that we fully validated, let-7a and miR-30a were another 2 downregulated miRNAs in HMCs upon stimulation with anti-dsDNA IgG antibodies that were interesting and needed further validation. Based on predicted target genes of let-7a, we hypothesized that this miRNA should be another important miRNA that regulates cell proliferation and cell apoptosis. Interestingly, our analysis clearly showed that *IL6* was a target of let-7a. However, a previous study in a mouse model of spontaneous LN showed upregulation of let-7a enhanced *IL6* expression in mesangial cells during active stages^50^. It is possible that the target of miRNA might be species-specific or the expression *in vivo* might be more complicated as a result of complex interplay from various factors. However, study in human LN showed that let-7a was downregulated in kidney biopsy from paediatric LN class III and IV but upregulated in LN class II^17,51^. Further study to characterize the functional role of let-7a in HMCs is required. Down-regulation of miR-30a after anti-dsDNA IgG stimulation was also demonstrated in this study. The down-regulation of miR-30a in kidney biopsy was found in various kidney diseases including focal segmental glomerulosclerosis and glomerular minor lesion^52^. Similar to let-7a, the miR-30a was previously reported to be upregulated in kidney biopsy from LN class II but downregulated in other classes of LN^17,51^. Unfortunately, due to limited tissue availability, we could not investigate level of these 2 miRNAs in kidney biopsy in our study.

Microarray profiles of antibody-stimulated HMCs revealed several upregulated genes in pathways involved in cell proliferation, apoptosis, extracellular matrix, and interferon type I signalling. Interferon type I signalling is upregulated in SLE patients and in kidney biopsies from LN patients^53^, suggesting our *in vitro* model can at least be used to study the regulation of inflammatory responses in mesangial cells. The current study suggested that anti-dsDNA IgG antibodies disrupted cell proliferation and cytokine production in HMCs, partly by downregulating miR-10a expression. This finding was strengthening by the fact that miR-10a expression was significantly downregulated in kidney biopsies from LN patients with severe mesangial cell proliferation (proliferative LN; PLN, class III and IV). Interestingly, elevated anti-dsDNA antibody titre was marked as risk factor for proliferative LN (class III or IV)^6^. However, in LN patients with mixed proliferative LN (MPLN; the proliferative LN co-exist with membranous LN, class III + V or IV + V), the miR-10a expression was not significantly downregulated. This could partly be explained by a different pathogenesis between MPLN and PLN as suggested by previous studies that MPLN was associated with poor renal outcome and low probability to enter complete remission compared to PLN^54^. However, it should be noted that the expression experiment was performed in a limited number of kidney biopsies and this finding should be validated in more samples.

**Figure 8:**
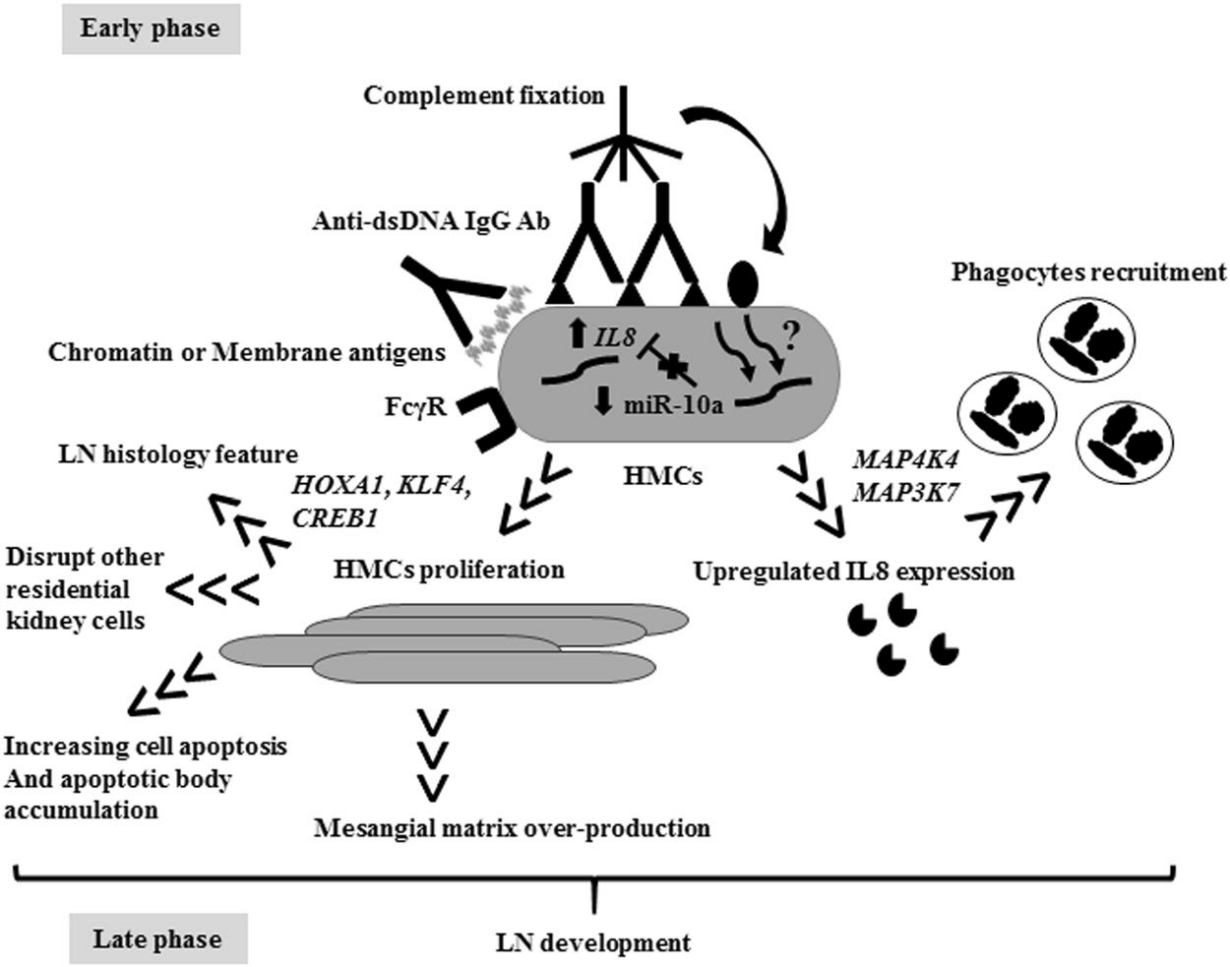
Putative mechanism of auto antibody mediated human mesangial cell induced inflammation and aberrant miRNA downstream regulation. In the early phase, auto antibodies (Ab) attach to chromatin structure or membrane antigens on human mesangial cell (HMC) membranes inducing complement fixation. The miR-10a is downregulated and enhances cell proliferation or apoptosis through *HOXA1*, *KLF4*, and *CREB1*. *IL8* or *CXCL8* are putative direct targets of miR-10a. The IL-8 attracts phagocytes into the kidney. HMCs interact with endothelial cells and the glomerular basement membrane, and abnormal functions of HMCs might disturb other resident kidney cell functions. The increase in HMC number might increase mesangial matrixes resulting in chromatin or apoptotic body accumulation. In summary, these processes might drive the progression of lupus nephritis (LN) disease and provide insights into LN pathogenesis.

Because HMCs are found specifically in kidneys, drugs targeted against HMCs might be useful to reduce various side effects caused by aggressive immunosuppressants in active LN patients. Previous studies of miRNAs in HMCs mainly focused on the late phase of inflammation, especially extracellular matrix production and kidney fibrosis^55,56^. In conclusion, several candidate miRNAs were altered by anti-dsDNA IgG antibody induction, which controlled cell proliferation and pro-inflammatory cytokine expression. Furthermore, anti-dsDNA antibodies downregulated miR-10a expression, which induces various target genes resulting in HMC proliferation and *IL8* gene expression (Fig. 8). Because one miRNA is capable of regulating many key phenotypes of HMC, which are important in LN pathogenesis, the manipulation of key miRNA including miR-10a might be a potential target for therapy. Moreover, its potential role as a biomarker to predict disease severity, early flares or treatment responses requires further exploration.

## Materials and Methods

### Sample Collection

The study was performed at the Nephrology Unit, Department of Medicine, Chulalongkorn Memorial Hospital. The subjects were recruited from 2013–2014. Sera (3 mL) were collected from active LN patients (N = 20) and healthy controls (N = 20) from the Thai Red Cross-National Blood Centre. All patients fulfilled at least 4 of 11 criteria according to the American College of Rheumatology diagnostic criteria with kidney biopsy proven. Patients in the active stage were defined by a urine protein creatinine index (UPCI) > 1.0 and the presence of RBCs and WBCs in the urine > 5 cells/HPF, or presence of course granular cast without infection. For kidney biopsies, forty-two samples were collected from active LN patients after histological examination. The classification is based on The International Society of Nephrology (ISN)/ Renal Pathology Society (RPS) criteria (2003). All the protocols were approved by the Ethical Committee of the Faculty of Medicine, Chulalongkorn University, Thailand (EC. No. 268/56 and EC. No. 136/59) and conducted based on the approved guidelines by the Declaration of Helsinki. All the subjects provided written consent form. Demographic data are shown in Table 3.

**Table 3:** Lupus nephritis patient demographic data and clinical scores.

Characteristics	Active LN	Healthy Controls	P-value
Number	20	20	NA
Sex (F/M)	19/1	19/1	NA
Age (average, s.d.)	33.75, 8.52	38.3, 8.93	0.1518
Clinical parameters (average, s.d.)			
Serum creatinine (mg/dL)	1.002, 0.1065	NA	NA
Proteinuria (g/day)	3.081, 0.3996	NA	NA
Urinary Erythrocyte Count (/HPF)	10.55, 4.863	NA	NA
MDRD^a^ GFR (mL/min)	82.08, 7.259	NA	NA
LN Renal Histology			
Class III	1/20	NA	NA
Class IV	11/20	NA	NA
Class V	2/20	NA	NA
Class III + V	1/20	NA	NA
Class IV + V	5/20	NA	NA
Activity index (average, s.d.)	5.9, 3.63	NA	NA
Chronicity index (average, s.d.)	5.2 ± 3.85	NA	NA
Steroid dose (mg/day) (average, s.d.)	19.63 ± 2.829	NA	NA

F, female; M, male; NA, not available; s.d., standard deviation; GFR, glomerular filtration rate; MDRD, The Modification of Diet in Renal Disease; HPF, high power field; LN, Lupus nephritis.

^a^The equation to calculate estimate glomerular filtration rate for Thais is 375.5 × serum Creatinine (−0.848) × age (−0.364) × 0.712 (if female).

### Polyclonal anti-dsDNA IgG antibody and non-specific IgG preparation

To purify anti-dsDNA IgG antibodies, active LN serum (N = 20) were pooled, each serum samples (3 mL) was diluted five times with 1X PBS (pH 7.4) and applied onto a protein G sepharose column (2 × 16 mm, GE Healthcare Life Science, Bangkok, Thailand). Similar processes were done with pooled healthy serum (N = 20) to isolate non-specific IgG controls. The matrix was subsequently washed with equilibrating buffer to remove unbound proteins. Bound antibodies were eluted with 0.1 M glycine buffer (pH 2.6) and the collected fractions were immediately neutralised with 1 M Tris-HCl buffer (pH 9). Non-specific IgG controls were dialyzed in 1X PBS overnight. To purify anti-dsDNA IgG antibodies, previously dialyzed against 25 mM Tris-HCl buffer (pH 7.4) containing 1 mM EDTA, 50 mM NaCl, 1 mM β-mercaptoethanol, and 10% glycerol, were loaded onto a DNA-cellulose column (3 × 16 mm, GE Healthcare Life Science). The anti-dsDNA IgG were eluted by 2 M NaCl and dialyzed against PBS (pH 7.4). The antibodies were stored at −80 °C until used.

### Measurements of antibody purity, activity and concentration

Antibody activity and purity were examined by Direct ELISA (cat no. EA 1572–9601 G, Euroimmun, Lübeck, Germany) and SDS-PAGE, respectively (Fig. S2). Antibody activity was also confirmed by immunofluorescence against HEp-2 cells. Purified anti-dsDNA IgG antibodies showed a homogenous staining pattern under an immunofluorescent microscope. Total IgG concentration was determined by nephelometry (BN Prospec System, Siemens, Berlin, Germany). The antibodies were filtered through a 0.22 μM filter before being used in experiments.

### Primary HMC culture and antibody stimulation

HMCs (Sciencell, Carlsbad, CA, USA), passages 4–7, were cultured in mesangial culture medium (MCM) at 37 °C at 5% CO_2_. The cells (1 × 10^5^ cells/well in a 24-well plate) were cultured in serum free medium for 24 hours before incubation with serum (1:10, 1:100, 1:1000) for 0, 3, 6, 12, and 24 hours. For antibody stimulation, the cells were incubated with anti-dsDNA IgG antibodies or non-specific IgG (10 µg/mL) with or without serum (1:100) for 3 hours. For heat inactivated serum, the sera were heated at 56 °C for 30 minutes before use.

### Flow cytometry

Flow cytometry were conducted as previously described^57^. Briefly, confluent HMCs (1 × 10^6^ cells) were trypsinised and cultured in suspension overnight (EBSS + 2% FBS, no agitation). The cells were pelleted and washed with FACs buffer (DPBS + 2% FBS) 3 times. Next, anti-dsDNA IgG antibodies or non-specific IgG (10, 20, 50, 100 µg/ml) were incubated for 1 hour on ice, then labelled with anti-human IgG Fc region-FITC or isotype control (BioLegend, San Diego, CA, USA). Fcγ receptor blocking reagent (Miltenyi Biotec, Bergisch Gladbach) was pre-incubated with HMCs (1:5 reagent volume per cells) for 10 minutes at 4 °C in the dark to confirm binding specificity. HMCs were also labeled with anti-HLA class I-FITC (clone W6/32) or anti-PDGFR-β-FITC antibodies (BioLegend) as a positive control. Flow cytometric results were analysed by FlowJo software.

### RNA preparation

Total RNA and miRNA were extracted using mirVana® small RNA extraction kit (Invitrogen Life Technologies, Waltham, MA, USA) using small RNA enrichment procedures. This method would help to obtain two separate fractions containing large RNA and small RNA. Purified RNA samples were processed with DNase treatment, aliquoted and stored at −80 °C until use. RNA purity and integrity were evaluated based on the OD 260/280 ratio and analysed on an Agilent 2100 Bioanalyzer (Agilent Technologies, Palo Alto, USA). Samples with a RIN number > 8 and a ratio between 28 s rRNA and 18 s rRNA > 1.6 were considered for further microarray and small RNASeq analysis.

### RT-PCR and qPCR

The isolated total RNA fraction was retro-transcribed using the Applied Biosystems™ High Capacity cDNA Reverse Transcription kit (Thermo Fisher Scientific, Waltham, MA, USA). Real-Time PCR was carried out using the Applied Biosystems® 7500 Real-Time PCR System. A list of primers is shown in Supplementary Table 6. The miRNAs were amplified individually using miR-10a (ID000387), miR-10b (ID002218), miR-143 (ID002249), miR-411 (ID001610), miR-181a (ID000480), miR-125a (ID002198), miR-125b-1* (ID002378), miR-127 (ID002229), miR-654 (ID002239), miR-30a (ID000417), let-7a (ID000377) and RNU44 (ID001094) assays (Invitrogen Life Technologies). Expressions were determined by C_T_ and expression fold change was calculated as previously described^58^.

### cDNA microarray

For cDNA microarray, two sets of experiment were performed as the following: 1) RNA from pooled anti-dsDNA IgG antibody stimulated HMCs VS pooled IgG control stimulated HMCs (Noted that these were the same samples used in miRNA sequencing experiment, but triplicate samples per group were used in cDNA microarray experiments) and 2) transient miR-10a knockdown HMCs VS scramble control (N = 3/group). Large RNA was amplified and purified using the Ambion Illumina RNA amplification kit (Ambion, Austin, USA) to yield biotinylated cRNA according to the manufacturer’s instructions. Briefly, 550 ng of total RNA was retro-transcribed to cDNA using a T7 oligo(dT) primer. Second-strand cDNA was synthesised and labelled with biotinylated-NTP. After purification, the cRNA was quantified using an ND-1000 Spectrophotometer (NanoDrop, Wilmington, USA). Then, 750 ng of labelled cRNA samples were hybridised to human HT-12 expression v.4 bead array for 16–18 h at 58 °C, according to the manufacturer’s instructions (Illumina Inc., San Diego, USA). Detection of array signal was carried out using Amersham fluorolink streptavidin-Cy3 (GE Healthcare Bio-Sciences, Little Chalfont, UK) following the bead array manual. Arrays were scanned with an Illumina bead array reader confocal scanner according to the manufacturer’s instructions. The quality of hybridisation and overall chip performance were monitored by visual inspection of both internal quality control checks and the raw scanned data. Raw data were extracted using the software provided by the manufacturer (Illumina GenomeStudio v2011.1; Gene Expression Module v1.9.0). Array probes were transformed by logarithm and normalised by the quantile method. The statistical significance of all expression data was determined using an unpaired *t-*test and fold change, in which the null hypothesis was that no difference existed among the groups. The false discovery rate (FDR) was controlled by adjusting p-values using the FDR algorithm (p-value < 0.05). For a DEG set, hierarchical cluster analysis was performed using complete linkage and Euclidean distance as a measure of similarity. KEGG pathway analysis, Gene Set Enrichment (GSE), Gene Ontology (GO) and Functional Annotation analysis for significant probe lists and predicted miRNA predicted target genes from integrative analysis were performed using DAVID version 6.7 (http://david.abcc.ncifcrf.gov/home.jsp). All data analysis of differentially expressed genes were conducted using R 3.0.2 (lumi-package)^59^. The microarray data from anti-dsDNA IgG antibody stimulated HMCs and transient knockdown of miR-10a in HMCs were deposited in the GEO database (repository numbers GSE80364 and GSE79574, respectively).

### Small RNA library preparation, Next Generation Sequencing and sequence annotation

Small RNA extracted from HMCs treated with pooled anti-dsDNA IgG antibodies or pooled non-specific IgG controls were used for next generation sequencing (N = 1/group). Small RNA libraries were prepared using the TruSeq Small RNA library preparation kits (Illumina®, Bioactive Ltd, Thailand). Briefly, small amounts of RNA (1 µg) were pooled and ligated with the adapter using T4 RNA ligase. The processed RNA was retro-transcribed and amplified with a PCR primer. The cDNA libraries were selectively purified by 6% polyacrylamide gel electrophoresis using a custom RNA ladder and high-resolution ladder as MW references. The prepared library was denatured with NaOH before loading. Sequencing was run using a MiSeq Sequencing System (Illumina®). The sequencing data were imported by Illumina® GenomeStudio Software. Data were analysed by using two bioinformatics tools including miRDeep 2.0^60^ and sRNAbench^61^. Sequencing quality was evaluated by FastQC. The sequences were aligned to the miRbase 20 database. The number of amplicons was normalised to reads per million reads (RPM).

### miRNA transfection

The HMCs were transfected with a miR-10a inhibitor, miR-10a mimic and scramble controls using Lipofectamine RNAiMax (oligonucleotides 5 pmol: RNAiMax 1.5 µL/well) (Thermo Fisher Scientific) for 24, 48 and 72 hours. Transfection efficiency were calculated by (2^(−ΔΔCT)^ × 100)_._ This represents the remaining percentage of miR-10a expression. The log (10) transform was applied to show transfection efficiency in miR-10a overexpression.

### Cell Proliferation assay

Cell proliferation assays were evaluated by adding MTS (Cell Aqueous One Solution Cell Proliferation Assay, Promega, Madison, WI, USA). Cell density was detected by measuring light absorbance (OD 490) using Verioskan (Thermo Fisher Scientific). To verify the number of cells according to absorbance, a standard curve was generated using various numbers of cells from 5,000 to 60,000 in triplicates. The absorbance was recorded according to the methods described above. For LPS stimulation, the cells were transfected with miR-10a inhibitor, miR-10a mimic and scramble for 24 hours. In the following, LPS (10 ng/mL) were incubated with HMCs for 24 hours. Cell proliferation was determined after stimulation.

### Dual Luciferase assay

The pmirGLO dual luciferase miRNA Target expression vector (Promega) was used. The miR-10a binding sites and binding capacity in the presence of the 3′UTR of *IL8* and *NFAT5* were predicted using TargetScan^57^, miRanda^62^ and RNAhybrid^63^ (Fig. 7C). These sequences were inserted into the pmirGLO plasmid, and were transformed into *Escherichia coli* (TOP10) and selected using ampicillin (100 μg). The plasmids were extracted using the Fast and Easy Plasmid Prep kit (JenaBioscience, Jena, Germany) and endotoxin-free plasmid preparation kit (Cat. No. 12362, Qiagen, Thailand). The insertion was confirmed by Sanger sequencing. pmirGlo containing the 3′UTR of *IL8* or the 3′ of *NFAT5* was co-transfected with miR-10a inhibitor, miR-10a mimic or scramble sequences for 48 hours using Lipofectamine 2000 (DNA 500 ng: 15 pmol RNA: Lipofectamine 2.5 µL/24 wells) (Thermo Fisher Scientific). Luciferase activity was determined using Dual-Glo® Luciferase assay systems (Promega). Luciferase activity was detected using Varioskan (Thermo Fisher Scientific) according to manufacturer’s instruction.

### Statistical analysis

Expression data were compared using an unpaired Student’s *t-*test. The non-parametric Mann-Whitney *U*-test was used to draw comparisons between groups, or an unpaired *t-*test was used to compare reporter gene activity. To compare differences between groups, a two-way ANOVA was applied. For the correlation study, the Pearson-Spearman technique was used. *P-*values less than 0.05 were considered statistically significant.

## Electronic supplementary material

Supplementary figures and tables
