# Supplementary material for: Transcriptomic profiling in human mesangial cells using patient-derived lupus autoantibodies identified miR-10a as a potential regulator of IL8
Source: Sci Rep. 2017 Nov 6;7:14517. doi: 10.1038/s41598-017-15160-8 (PMC5673966; doi:10.1038/s41598-017-15160-8)
Supplement: Supplementary file 1 — Supplementary figures and tables [file 41598_2017_15160_MOESM1_ESM.doc]

**Supplementary Info**

**Transcriptomic profiling in human mesangial cells using patient-derived lupus autoantibodies identified miR-10a as a potential regulator of *IL8***

Pattarin Tangtanatakul, PhD^1,2^, Boonyakiat Thammasate, MD^2^, Alain Jacquet, PhD^3^, Rangsima Reantragoon, MD, PhD^2^, Trairak Pisitkun, MD^2,4^, Yingyos Avihingsanon, MD^2,3^, Asada Leelahavanichkul, MD, PhD^2^, Nattiya Hirankarn, MD, PhD^2^

^1^Medical Microbiology Interdisciplinary Program, Graduate School, Chulalongkorn University, Bangkok 10330, Thailand.

^2^Center of Excellence in Immunology and Immune-mediated Diseases, Department of Microbiology, Faculty of Medicine, Chulalongkorn University, Bangkok 10330, Thailand.

^3^Department of Medicine, Faculty of Medicine, Chulalongkorn University, Bangkok 10330, Thailand.

^4^Chulalongkorn University Systems Biology (CUSB), Faculty of Medicine, Chulalongkorn University, Bangkok, 10330, Thailand

*Corresponding author: Nattiya Hirankarn (E-mail: Nattiya.H@chula.ac.th)

**Figure S1. IL-6 expression in mesangial cells after serum stimulation.** Time-dependent (A) and dose-dependent (B) expression of IL-6.

**
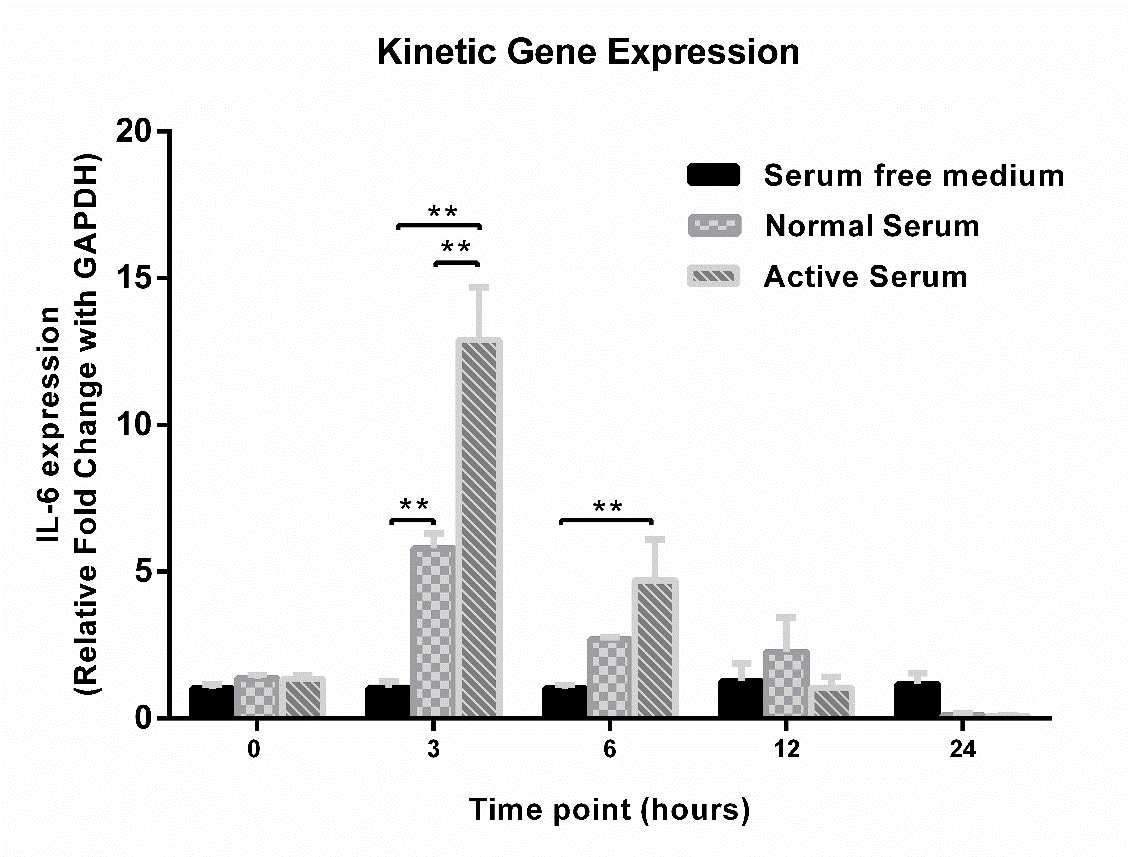
A.**

**B.**

**
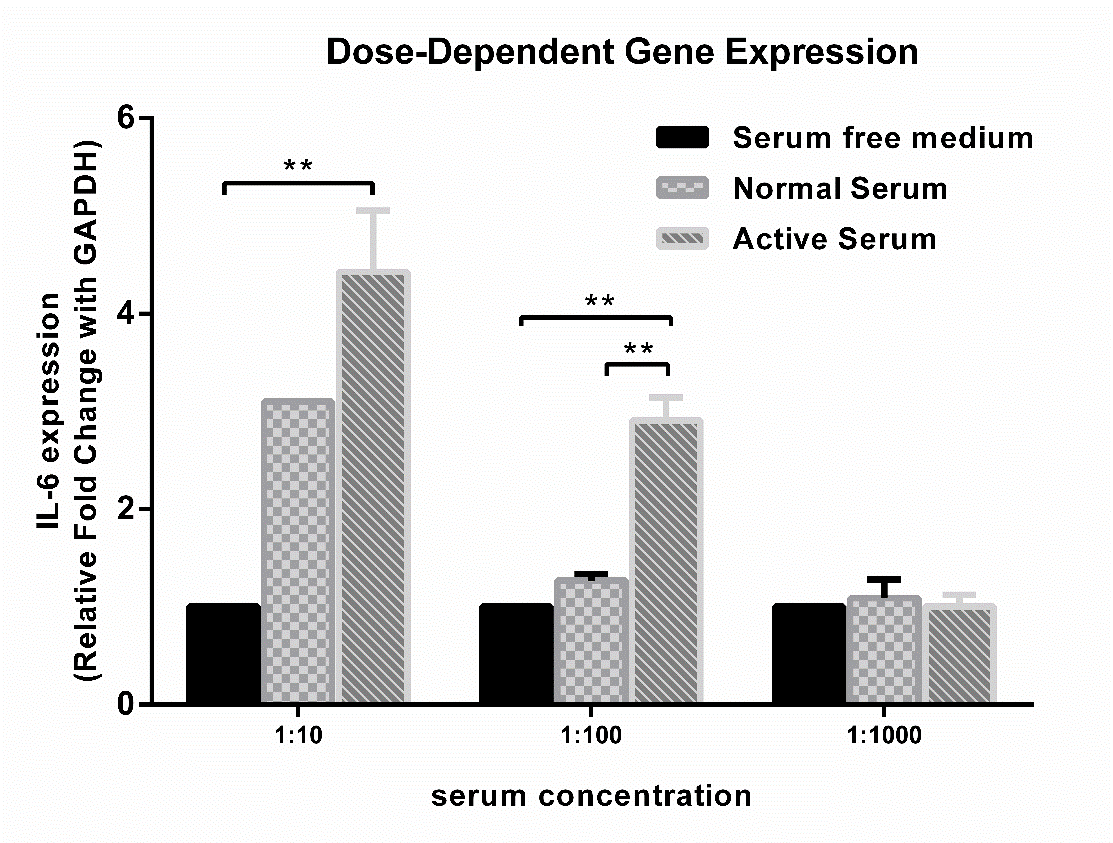
**

**Figure S2. Denaturing 10% SDS-PAGE shows antibody purity during sequential chromatography.**

**Marker L1 L2 L3 L4 L5**

**
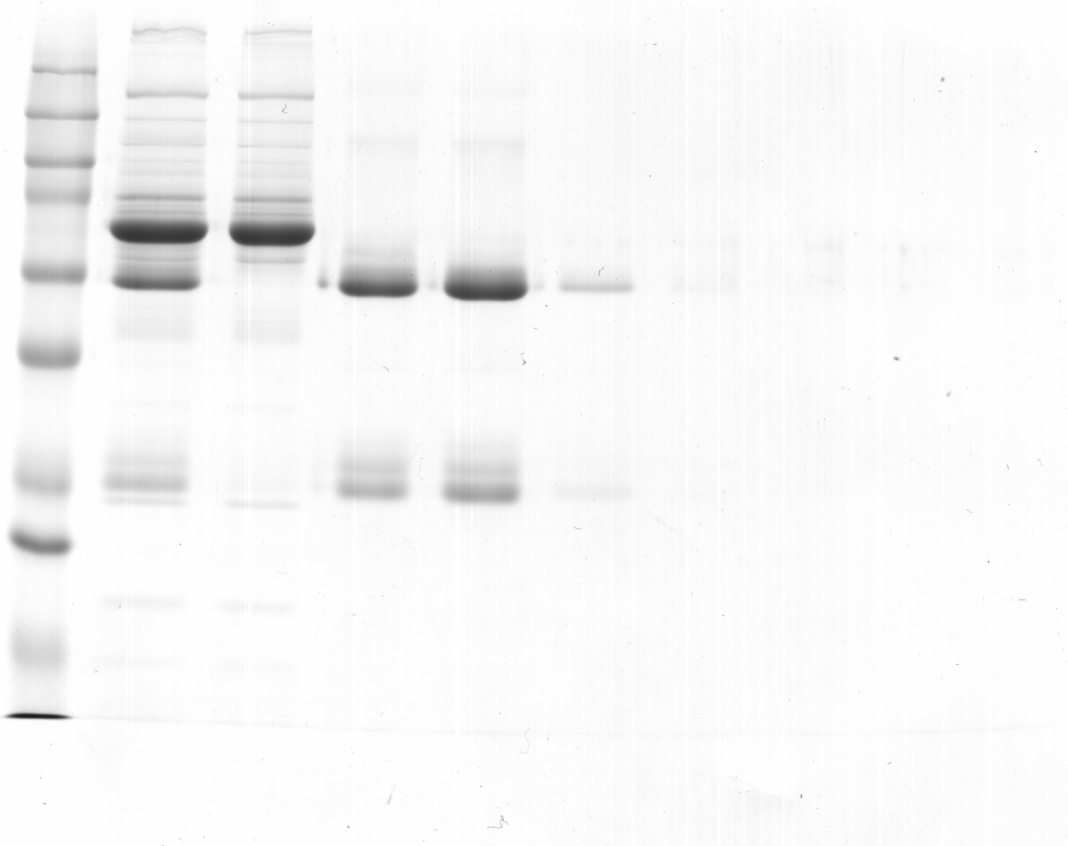
**

**Marker lane: Protein marker (PageRuler^TM^ Prestained Protein Ladder, cat.no. 26616, ThermoFisher Scientific, Thailand)**

**L1: Diluted crude serum (1:200)**

**L2: Diluted flow-through from sepharose protein G column (1:5)**

**L3: Diluted elution from sepharose protein G column (1:5) (non-specific IgG)**

**L4: Flow-through from DNA cellulose column**

**L5: Elution from DNA cellulose column (anti-dsDNA IgG antibodies)**

**Supplementary Table 1.** List of differentially expressed microRNA after anti-dsDNA IgG stimulation

| **miRNA** | **IgG (RPM)** | **anti-dsDNA (RPM)** | **Foldchange** |
| --- | --- | --- | --- |
| **hsa-miR-215-5p** | 4.113506129 | 112.1845032 | 27.27 |
| **hsa-miR-6819-5p** | 0.097940622 | 0.883342545 | 9.02 |
| **hsa-miR-7704** | 0.685584355 | 5.888950298 | 8.59 |
| **hsa-miR-4755-5p** | 0.195881244 | 1.472237574 | 7.52 |
| **hsa-miR-3944-5p** | 0.097940622 | 0.58889503 | 6.01 |
| **hsa-miR-320c** | 2.74233742 | 14.13348071 | 5.15 |
| **hsa-miR-4473** | 0.881465599 | 4.122265208 | 4.68 |
| **hsa-miR-3173-5p** | 0.195881244 | 0.883342545 | 4.51 |
| **hsa-miR-4727-5p** | 0.195881244 | 0.883342545 | 4.51 |
| **hsa-miR-5010-3p** | 0.195881244 | 0.883342545 | 4.51 |
| **hsa-miR-122-5p** | 0.293821866 | 1.17779006 | 4.01 |
| **hsa-miR-665** | 102.0541283 | 351.5703328 | 3.44 |
| **hsa-miR-1260b** | 370.2155516 | 1161.889894 | 3.14 |
| **hsa-miR-130b-5p** | 263.852036 | 808.5528759 | 3.06 |
| **hsa-miR-216a-5p** | 0.293821866 | 0.883342545 | 3.01 |
| **hsa-miR-3175** | 1.762931198 | 5.300055268 | 3.01 |
| **hsa-miR-3684** | 0.489703111 | 1.472237574 | 3.01 |
| **hsa-miR-4661-5p** | 0.489703111 | 1.472237574 | 3.01 |
| **hsa-miR-373-5p** | 0.195881244 | 0.58889503 | 3.01 |
| **hsa-miR-4717-5p** | 0.391762489 | 1.17779006 | 3.01 |
| **hsa-miR-597-5p** | 0.391762489 | 1.17779006 | 3.01 |
| **hsa-miR-1255a** | 1.37116871 | 3.827817693 | 2.79 |
| **hsa-miR-129-5p** | 1.175287466 | 3.238922664 | 2.76 |
| **hsa-miR-1245a** | 1.37116871 | 3.533370179 | 2.58 |
| **hsa-miR-1249** | 1.37116871 | 3.533370179 | 2.58 |
| **hsa-miR-33b-5p** | 8.912596614 | 22.96690616 | 2.58 |
| **hsa-miR-92a-2-5p** | 400.5771445 | 1002.004893 | 2.50 |
| **hsa-miR-7976** | 1.664990576 | 4.122265208 | 2.48 |
| **hsa-miR-212-5p** | 47.10943924 | 110.4178181 | 2.34 |
| **hsa-miR-4707-5p** | 0.881465599 | 2.061132604 | 2.34 |
| **hsa-miR-194-5p** | 14.39727145 | 31.80033161 | 2.21 |
| **hsa-miR-320b** | 12.04669652 | 25.61693379 | 2.13 |
| **hsa-miR-654-5p** | 3932.413919 | 8308.425527 | 2.11 |
| **hsa-miR-3152-5p** | 18.80459945 | 38.86707196 | 2.07 |
| **hsa-miR-128-2-5p** | 33.49569277 | 69.195166 | 2.07 |
| **hsa-miR-1307-5p** | 678.9243926 | 1388.320033 | 2.04 |
| **hsa-miR-3115** | 0.293821866 | 0.58889503 | 2.00 |
| **hsa-miR-4642** | 0.293821866 | 0.58889503 | 2.00 |
| **hsa-miR-5581-5p** | 0.587643733 | 1.17779006 | 2.00 |
| **hsa-miR-6516-5p** | 0.587643733 | 1.17779006 | 2.00 |
| **hsa-miR-6815-5p** | 0.293821866 | 0.58889503 | 2.00 |
| **hsa-miR-9-5p** | 3.525862397 | 7.066740357 | 2.00 |
| **hsa-miR-24-2-5p** | 769.7153493 | 1513.460226 | 1.97 |
| **hsa-miR-24-3p** | 769.7153493 | 1513.460226 | 1.97 |
| **hsa-miR-24-1-5p** | 770.0091712 | 1513.754674 | 1.97 |
| **hsa-miR-431-5p** | 369.1382048 | 723.7519916 | 1.96 |
| **hsa-miR-27a-5p** | 3560.141614 | 6923.344417 | 1.94 |
| **hsa-miR-584-5p** | 72.47606037 | 140.7459121 | 1.94 |
| **hsa-miR-302d-5p** | 1.37116871 | 2.650027634 | 1.93 |
| **hsa-miR-3934-5p** | 1.077346843 | 2.061132604 | 1.91 |
| **hsa-miR-302a-5p** | 1.86087182 | 3.533370179 | 1.90 |
| **hsa-miR-940** | 6.46408106 | 12.07234811 | 1.87 |
| **hsa-miR-652-5p** | 17.33549012 | 32.09477912 | 1.85 |
| **hsa-miR-3177-5p** | 1.273228088 | 2.355580119 | 1.85 |
| **hsa-miR-107** | 229.0831152 | 423.1210789 | 1.85 |
| **hsa-miR-1294** | 1.762931198 | 3.238922664 | 1.84 |
| **hsa-miR-210-5p** | 170.2208013 | 310.9365757 | 1.83 |
| **hsa-miR-766-5p** | 21.74281811 | 39.45596699 | 1.81 |
| **hsa-miR-302c-5p** | 0.489703111 | 0.883342545 | 1.80 |
| **hsa-miR-3662** | 0.489703111 | 0.883342545 | 1.80 |
| **hsa-miR-200b-5p** | 6.855843549 | 12.36679562 | 1.80 |
| **hsa-miR-410-5p** | 1985.648173 | 3551.920372 | 1.79 |
| **hsa-miR-323a-5p** | 131.3383743 | 234.0857743 | 1.78 |
| **hsa-miR-151b** | 6849.575349 | 12139.1877 | 1.77 |
| **hsa-miR-345-5p** | 483.6307921 | 847.1255003 | 1.75 |
| **hsa-miR-143-5p** | 23344.44111 | 40170.88556 | 1.72 |
| **hsa-miR-1322** | 7.541427904 | 12.95569065 | 1.72 |
| **hsa-miR-134-5p** | 327.4154998 | 562.1003059 | 1.72 |
| **hsa-miR-33a-5p** | 16.84578701 | 28.85585646 | 1.71 |
| **hsa-miR-889-5p** | 612.1288883 | 1034.099672 | 1.69 |
| **hsa-miR-199b-5p** | 5506.417657 | 9300.124757 | 1.69 |
| **hsa-miR-199a-5p** | 11018.41793 | 18603.78289 | 1.69 |
| **hsa-miR-181b-5p** | 4031.627769 | 6753.153754 | 1.68 |
| **hsa-miR-126-5p** | 94.02299724 | 156.9405254 | 1.67 |
| **hsa-miR-615-5p** | 412.1341379 | 684.0015771 | 1.66 |
| **hsa-miR-532-5p** | 74.33693219 | 123.3735087 | 1.66 |
| **hsa-miR-483-5p** | 52.10441097 | 86.27312186 | 1.66 |
| **hsa-miR-127-5p** | 23325.44063 | 38570.56332 | 1.65 |
| **hsa-miR-3661** | 6.268199816 | 10.30566302 | 1.64 |
| **hsa-miR-708-5p** | 55.43439212 | 90.68983458 | 1.64 |
| **hsa-miR-25-5p** | 1856.660374 | 3011.314735 | 1.62 |
| **hsa-miR-181a-5p** | 20669.68272 | 33518.13841 | 1.62 |
| **hsa-miR-589-5p** | 241.2277523 | 390.7318522 | 1.62 |
| **hsa-miR-1185-5p** | 160.8185015 | 259.7027081 | 1.61 |
| **hsa-miR-92a-1-5p** | 9706.699178 | 15669.31895 | 1.61 |
| **hsa-miR-106b-5p** | 119.1937371 | 192.2742272 | 1.61 |
| **hsa-miR-484** | 383.9272387 | 618.0453337 | 1.61 |
| **hsa-miR-487b-5p** | 228.4954714 | 367.7649461 | 1.61 |
| **hsa-miR-411-5p** | 4311.150305 | 6911.272069 | 1.60 |
| **hsa-miR-27b-5p** | 15093.13957 | 23812.55942 | 1.58 |
| **hsa-miR-192-5p** | 354.2512302 | 556.5058031 | 1.57 |
| **hsa-miR-132-5p** | 166.7928795 | 260.2916032 | 1.56 |
| **hsa-miR-140-5p** | 401.0668476 | 604.7951956 | 1.51 |
| **hsa-miR-218-5p** | 408.3144536 | 613.9230685 | 1.50 |
| **hsa-miR-1303** | 3.721743641 | 5.594502783 | 1.50 |
| **hsa-miR-487a-5p** | 89.71360987 | 134.8569618 | 1.50 |
| **hsa-miR-5094** | 2.350574931 | 3.533370179 | 1.50 |
| **hsa-miR-3126-5p** | 0.783524977 | 1.17779006 | 1.50 |
| **hsa-miR-3145-5p** | 0.391762489 | 0.58889503 | 1.50 |
| **hsa-miR-3918** | 0.391762489 | 0.58889503 | 1.50 |
| **hsa-miR-3942-5p** | 0.783524977 | 1.17779006 | 1.50 |
| **hsa-miR-4786-5p** | 1.567049954 | 2.355580119 | 1.50 |
| **hsa-miR-499a-5p** | 0.783524977 | 1.17779006 | 1.50 |
| **hsa-miR-339-5p** | 65.03257309 | 97.46212742 | 1.50 |
| **hsa-miR-186-5p** | 3217.153556 | 2557.865562 | 0.80 |
| **hsa-miR-185-5p** | 24.87691802 | 19.7279835 | 0.79 |
| **hsa-miR-125a-5p** | 8162.273507 | 6471.661929 | 0.79 |
| **hsa-miR-668-5p** | 12.73228088 | 10.01121551 | 0.79 |
| **hsa-miR-30a-5p** | 12534.04906 | 9851.330505 | 0.79 |
| **hsa-miR-335-5p** | 1983.101717 | 1532.893762 | 0.77 |
| **hsa-miR-196b-5p** | 624.7632285 | 479.9494493 | 0.77 |
| **hsa-miR-196a-5p** | 2839.788339 | 2166.544814 | 0.76 |
| **hsa-miR-23a-5p** | 1845.10338 | 1394.797878 | 0.76 |
| **hsa-miR-539-5p** | 168.9475732 | 127.4957739 | 0.75 |
| **hsa-miR-501-5p** | 129.1836806 | 96.87323239 | 0.75 |
| **hsa-miR-146b-5p** | 1069.903356 | 784.4081796 | 0.73 |
| **hsa-miR-377-5p** | 78.3524977 | 56.82837037 | 0.73 |
| **hsa-miR-331-5p** | 44.95474556 | 32.38922664 | 0.72 |
| **hsa-miR-455-5p** | 124.2866495 | 87.45091192 | 0.70 |
| **hsa-miR-7-5p** | 23.21192744 | 16.19461332 | 0.70 |
| **hsa-miR-590-5p** | 34.9648021 | 23.85024871 | 0.68 |
| **hsa-let-7a-5p** | 110934.0127 | 75416.84199 | 0.68 |
| **hsa-miR-500a-5p** | 276.9760794 | 184.9130393 | 0.67 |
| **hsa-miR-495-5p** | 62.87787941 | 41.5170996 | 0.66 |
| **hsa-miR-548e-5p** | 9.989943457 | 6.477845327 | 0.65 |
| **hsa-miR-374a-5p** | 203.9123753 | 130.7346966 | 0.64 |
| **hsa-miR-424-5p** | 378.4425639 | 239.0913821 | 0.63 |
| **hsa-miR-337-5p** | 65.71815745 | 40.92820457 | 0.62 |
| **hsa-miR-548au-5p** | 41.6247644 | 22.67245865 | 0.54 |
| **hsa-miR-10a-5p** | 75054.24931 | 39070.5352 | 0.52 |
| **hsa-miR-664a-5p** | 16.25814327 | 8.244530417 | 0.51 |
| **hsa-miR-10b-5p** | 145632.3184 | 65573.75601 | 0.45 |
| **hsa-miR-5690** | 4.309387374 | 1.766685089 | 0.41 |
| **hsa-miR-145-5p** | 353.1738834 | 119.545691 | 0.34 |
| **hsa-miR-190a-5p** | 64.6408106 | 17.66685089 | 0.27 |
| **hsa-miR-19b-1-5p** | 112.3378936 | 29.739199 | 0.26 |
| **hsa-miR-19b-2-5p** | 356.2100427 | 80.67861908 | 0.23 |
| **hsa-miR-7974** | 145.0500614 | 29.15030397 | 0.20 |

**Supplementary Table 2.** Downregulated genes in HMCs treated with anti-dsDNA antibodies that contain a target binding site for miR-654.

| **ID** | **RefSeq** | **Gene Name** |
| --- | --- | --- |
| **ADAP2** | NM_018404.2 | ArfGAP with dual PH domains 2(ADAP2) |
| **AFF3** | NM_001025108.1 | AF4/FMR2 family member 3(AFF3) |
| **AKT3** | NM_181690.1 | AKT serine/threonine kinase 3(AKT3) |
| **AP3S2** | NM_005829.3 | adaptor related protein complex 3 sigma 2 subunit(AP3S2) |
| **ARL17A/ARL17B** | NM_001039083.3 | ADP ribosylation factor like GTPase 17B |
| **ARNT2** | NM_014862.3 | aryl hydrocarbon receptor nuclear translocator 2(ARNT2) |
| **B4GALT4** | NM_003778.3 | beta-1,4-galactosyltransferase 4(B4GALT4) |
| **BCL9L** | NM_182557.2 | B-cell CLL/lymphoma 9-like(BCL9L) |
| **C15orf40** | NM_144597.1 | chromosome 15 open reading frame 40(C15orf40) |
| **CCDC74B** | NM_207310.1 | coiled-coil domain containing 74B(CCDC74B) |
| **CD276** | NM_025240.2 | CD276 molecule(CD276) |
| **CD3EAP** | NM_012099.1 | CD3e molecule associated protein(CD3EAP) |
| **CDCP1** | NM_178181.1 | CUB domain containing protein 1(CDCP1) |
| **CHM** | NM_000390.2 | CHM, Rab escort protein 1(CHM) |
| **CNOT4** | NM_013316.2 | CCR4-NOT transcription complex subunit 4(CNOT4) |
| **CSHL1** | NM_022579.1 | chorionic somatomammotropin hormone like 1(CSHL1) |
| **CXorf36** | NM_024689.1 | chromosome X open reading frame 36(CXorf36) |
| **CYB5R3** | NM_000398.4 | cytochrome b5 reductase 3(CYB5R3) |
| **DNAJC24** | NM_181706.4 | DnaJ heat shock protein family (Hsp40) member C24(DNAJC24) |
| **DTX3L** | NM_138287.2 | deltex E3 ubiquitin ligase 3L(DTX3L) |
| **DYNLL2** | NM_080677.1 | dynein light chain LC8-type 2(DYNLL2) |
| **EFNB1** | NM_004429.3 | ephrin B1(EFNB1) |
| **EPHA8** | NM_020526.3 | EPH receptor A8(EPHA8) |
| **ERGIC1** | NM_020462.1 | endoplasmic reticulum-golgi intermediate compartment 1(ERGIC1) |
| **FOSL2** | NM_005253.3 | FOS like 2, AP-1 transcription factor subunit(FOSL2) |
| **GEM** | NM_181702.1 | GTP binding protein overexpressed in skeletal muscle(GEM) |
| **GOLGA3** | NM_005895.3 | golgin A3(GOLGA3) |
| **HDAC7** | NM_001098416.2 | histone deacetylase 7(HDAC7) |
| **HOOK3** | NM_032410.2 | hook microtubule tethering protein 3(HOOK3) |
| **IGF2BP3** | NM_006547.2 | insulin like growth factor 2 mRNA binding protein 3(IGF2BP3) |
| **ING1** | NM_198219.1 | inhibitor of growth family member 1(ING1) |
| **INO80E** | NM_173618.1 | INO80 complex subunit E(INO80E) |
| **IRS1** | NM_005544.1 | insulin receptor substrate 1(IRS1) |
| **ITM2C** | NM_001012516.1 | integral membrane protein 2C(ITM2C) |
| **IVD** | NM_002225.2 | isovaleryl-CoA dehydrogenase(IVD) |
| **KCTD11** | NM_001002914.2 | potassium channel tetramerization domain containing 11(KCTD11) |
| **KLF13** | NM_015995.2 | Kruppel like factor 13(KLF13) |
| **LRTOMT** | NM_145309.1 | leucine rich transmembrane and O-methyltransferase domain containing(LRTOMT) |
| **LSM12** | NM_152344.2 | LSM12 homolog(LSM12) |
| **LY6G5C** | NM_001002849.1 | lymphocyte antigen 6 complex, locus G5C(LY6G5C) |
| **LYSMD1** | NM_212551.3 | LysM domain containing 1(LYSMD1) |
| **LZTS1** | NM_021020.1 | leucine zipper tumor suppressor 1(LZTS1) |
| **MAPK14** | NM_139013.1 | mitogen-activated protein kinase 14(MAPK14) |
| **ME2** | NM_002396.3 | malic enzyme 2(ME2) |
| **MGLL** | NM_007283.5 | monoglyceride lipase(MGLL) |
| **MLEC** | NM_014730.2 | malectin(MLEC) |
| **MOCS1** | NM_005943.3 | molybdenum cofactor synthesis 1(MOCS1) |
| **MTPAP** | NM_018109.3 | mitochondrial poly(A) polymerase(MTPAP) |
| **MTSS1** | NM_014751.4 | MTSS1, I-BAR domain containing(MTSS1) |
| **NACC2** | NM_144653.3 | NACC family member 2(NACC2) |
| **NT5DC3** | NM_016575.1 | 5'-nucleotidase domain containing 3(NT5DC3) |
| **NTRK2** | NM_006180.3 | neurotrophic receptor tyrosine kinase 2(NTRK2) |
| **PARS2** | NM_152268.2 | prolyl-tRNA synthetase 2, mitochondrial (putative)(PARS2) |
| **PEA15** | NM_003768.2 | phosphoprotein enriched in astrocytes 15(PEA15) |
| **PKP2** | NM_001005242.1 | plakophilin 2(PKP2) |
| **PLAGL2** | NM_002657.2 | PLAG1 like zinc finger 2(PLAGL2) |
| **PPM1F** | NM_014634.2 | protein phosphatase, Mg2+/Mn2+ dependent 1F(PPM1F) |
| **PPM1M** | NM_144641.1 | protein phosphatase, Mg2+/Mn2+ dependent 1M(PPM1M) |
| **PSMD9** | NM_002813.4 | proteasome 26S subunit, non-ATPase 9(PSMD9) |
| **PTRH1** | NM_001002913.1 | peptidyl-tRNA hydrolase 1 homolog(PTRH1) |
| **RAB11FIP1** | NM_001002233.1 | RAB11 family interacting protein 1(RAB11FIP1) |
| **RAB40C** | NM_021168.2 | RAB40C, member RAS oncogene family(RAB40C) |
| **RCOR2** | NM_173587.2 | REST corepressor 2(RCOR2) |
| **RNF4** | NM_002938.2 | ring finger protein 4(RNF4) |
| **RNF41** | NM_194358.1 | ring finger protein 41(RNF41) |
| **RNPEPL1** | NM_018226.3 | arginyl aminopeptidase like 1(RNPEPL1) |
| **SCLY** | NM_016510.3 | selenocysteine lyase(SCLY) |
| **SEPT9** | NM_006640.3 | Homo sapiens septin 9, transcript variant 3, mRNA. |
| **SH2B3** | NM_005475.1 | SH2B adaptor protein 3(SH2B3) |
| **SH3PXD2B** | NM_001017995.1 | SH3 and PX domains 2B(SH3PXD2B) |
| **SIRT5** | NM_012241.2 | sirtuin 5(SIRT5) |
| **SLC6A8** | NM_005629.1 | solute carrier family 6 member 8(SLC6A8) |
| **SLCO3A1** | NM_013272.2 | solute carrier organic anion transporter family member 3A1(SLCO3A1) |
| **SMG5** | NM_015327.1 | SMG5, nonsense mediated mRNA decay factor(SMG5) |
| **ST5** | NM_213618.1 | suppression of tumorigenicity 5(ST5) |
| **STC1** | NM_003155.2 | stanniocalcin 1(STC1) |
| **STK38** | NM_007271.2 | serine/threonine kinase 38(STK38) |
| **STK40** | NM_032017.1 | serine/threonine kinase 40(STK40) |
| **SUPT16H** | NM_007192.2 | SPT16 homolog, facilitates chromatin remodeling subunit(SUPT16H) |
| **SYNGR1** | NM_145738.1 | synaptogyrin 1(SYNGR1) |
| **TEX261** | NM_144582.2 | testis expressed 261(TEX261) |
| **TMEM11** | NM_003876.1 | transmembrane protein 11(TMEM11) |
| **TMEM140** | NM_018295.2 | transmembrane protein 140(TMEM140) |
| **TPM2** | NM_213674.1 | tropomyosin 2 (beta)(TPM2) |
| **TRIL** | NM_014817.3 | TLR4 interactor with leucine rich repeats(TRIL) |
| **TRIM26** | NM_003449.3 | tripartite motif containing 26(TRIM26) |
| **TRIM5** | NM_033034.1 | tripartite motif containing 5(TRIM5) |
| **TSPAN9** | NM_006675.3 | tetraspanin 9(TSPAN9) |
| **UBXN2B** | NM_001077619.1 | UBX domain protein 2B(UBXN2B) |
| **UNC5C** | NM_003728.2 | unc-5 netrin receptor C(UNC5C) |
| **WDR82** | NM_025222.3 | WD repeat domain 82(WDR82) |
| **YKT6** | NM_006555.3 | YKT6 v-SNARE homolog (S. cerevisiae)(YKT6) |
| **ZBTB3** | NM_024784.2 | zinc finger and BTB domain containing 3(ZBTB3) |
| **ZBTB7A** | NM_015898.2 | zinc finger and BTB domain containing 7A(ZBTB7A) |
| **ZHX3** | NM_015035.3 | zinc fingers and homeoboxes 3(ZHX3) |
| **ZMIZ1** | NM_020338.2 | zinc finger MIZ-type containing 1(ZMIZ1) |
| **ZNF598** | NM_178167.2 | zinc finger protein 598(ZNF598) |
| **ZNF609** | NM_015042.1 | zinc finger protein 609(ZNF609) |

**Supplementary Table 3.** Upregulated genes in HMCs treated with anti-dsDNA antibodies that contain a target binding site for miR-30a.

| **ID** | **RefSeq_mRNA** | **Gene Name** |
| --- | --- | --- |
| **VPS13C** | NM_017684.3 | vacuolar protein sorting 13 homolog C(VPS13C) |
| **VPS26B** | NM_052875.3 | VPS26, retromer complex component B(VPS26B) |
| **WNK3** | NM_020922.2 | WNK lysine deficient protein kinase 3(WNK3) |
| **WWP1** | NM_007013.3 | WW domain containing E3 ubiquitin protein ligase 1(WWP1) |
| **ZBTB11** | NM_014415.2 | zinc finger and BTB domain containing 11(ZBTB11) |
| **ZBTB40** | NM_001083621.1 | zinc finger and BTB domain containing 40(ZBTB40) |
| **ZCCHC4** | NM_024936.2 | zinc finger CCHC-type containing 4(ZCCHC4) |
| **ZFYVE26** | NM_015346.2 | zinc finger FYVE-type containing 26(ZFYVE26) |
| **ZMYND8** | NM_183047.1 | zinc finger MYND-type containing 8(ZMYND8) |
| **ZNF518A** | NM_014803.3 | zinc finger protein 518A(ZNF518A) |
| **ZNF608** | NM_020747.2 | zinc finger protein 608(ZNF608) |
| **ZNF644** | NM_016620.2 | zinc finger protein 644(ZNF644) |
| **ZNF652** | NM_014897.1 | zinc finger protein 652(ZNF652) |
| **ZNF706** | NM_016096.3 | zinc finger protein 706(ZNF706) |
| **ZNF746** | NM_152557.3 | zinc finger protein 746(ZNF746) |
| **ZNF770** | NM_014106.2 | zinc finger protein 770(ZNF770) |
| **ABL1** | NM_007313.2 | ABL proto-oncogene 1, non-receptor tyrosine kinase(ABL1) |
| **ABL2** | NM_005158.3 | ABL proto-oncogene 2, non-receptor tyrosine kinase(ABL2) |
| **ACTC1** | NM_005159.4 | actin, alpha, cardiac muscle 1(ACTC1) |
| **ACTR1A** | NM_005736.2 | ARP1 actin-related protein 1 homolog A, centractin alpha(ACTR1A) |
| **ADAM12** | NM_003474.3 | ADAM metallopeptidase domain 12(ADAM12) |
| **ADAMTS6** | NM_197941.2 | ADAM metallopeptidase with thrombospondin type 1 motif 6(ADAMTS6) |
| **ADRA2A** | NM_000681.2 | adrenoceptor alpha 2A(ADRA2A) |
| **AHNAK** | NM_001620.1 | AHNAK nucleoprotein(AHNAK) |
| **AKAP10** | NM_007202.2 | A-kinase anchoring protein 10(AKAP10) |
| **ALG9** | NM_001077692.1 | ALG9, alpha-1,2-mannosyltransferase(ALG9) |
| **AMOTL2** | NM_016201.2 | angiomotin like 2(AMOTL2) |
| **ANKHD1/ANKHD1-EIF4EBP3** | NM_017747.1 | #N/A |
| **ANKRA2** | NM_023039.2 | ankyrin repeat family A member 2(ANKRA2) |
| **ANKRD20A4 (includes others)** | NM_032250.2 | #N/A |
| **AP1B1** | NM_145730.1 | adaptor related protein complex 1 beta 1 subunit(AP1B1) |
| **AP3M1** | NM_207012.2 | adaptor related protein complex 3 mu 1 subunit(AP3M1) |
| **ARL15** | NM_019087.1 | ADP ribosylation factor like GTPase 15(ARL15) |
| **ARL4C** | NM_005737.3 | ADP ribosylation factor like GTPase 4C(ARL4C) |
| **ASB3/GPR75-ASB3** | NM_016115.3 | #N/A |
| **ATAD2B** | NM_017552.1 | ATPase family, AAA domain containing 2B(ATAD2B) |
| **ATG5** | NM_004849.1 | autophagy related 5(ATG5) |
| **ATRN** | NM_139321.1 | attractin(ATRN) |
| **AVEN** | NM_020371.2 | apoptosis and caspase activation inhibitor(AVEN) |
| **AVL9** | NM_015060.1 | AVL9 cell migration associated(AVL9) |
| **BACH1** | NM_206866.1 | BTB domain and CNC homolog 1(BACH1) |
| **BAHD1** | NM_014952.3 | bromo adjacent homology domain containing 1(BAHD1) |
| **BAZ2B** | NM_013450.2 | bromodomain adjacent to zinc finger domain 2B(BAZ2B) |
| **BCL6** | NM_001706.2 | B-cell CLL/lymphoma 6(BCL6) |
| **BCL9** | NM_004326.2 | B-cell CLL/lymphoma 9(BCL9) |
| **BNC2** | NM_017637.5 | basonuclin 2(BNC2) |
| **C10orf76** | NM_024541.2 | chromosome 10 open reading frame 76(C10orf76) |
| **C12orf76** | NM_207435.1 | chromosome 12 open reading frame 76(C12orf76) |
| **C19orf12** | NM_001031726.2 | chromosome 19 open reading frame 12(C19orf12) |
| **C3orf18** | NM_016210.2 | chromosome 3 open reading frame 18(C3orf18) |
| **CABLES2** | NM_031215.2 | Cdk5 and Abl enzyme substrate 2(CABLES2) |
| **CALB2** | NM_007088.2 | calbindin 2(CALB2) |
| **CAPN5** | NM_004055.4 | calpain 5(CAPN5) |
| **CAPZA1** | NM_006135.2 | capping actin protein of muscle Z-line alpha subunit 1(CAPZA1) |
| **CARS** | NM_001014438.1 | cysteinyl-tRNA synthetase(CARS) |
| **CAT** | NM_001752.2 | catalase(CAT) |
| **CCDC97** | NM_052848.1 | coiled-coil domain containing 97(CCDC97) |
| **CCNJL** | NM_024565.5 | cyclin J like(CCNJL) |
| **CDC7** | NM_003503.2 | cell division cycle 7(CDC7) |
| **CDCA7** | NM_031942.4 | cell division cycle associated 7(CDCA7) |
| **CELSR3** | NM_001407.2 | cadherin EGF LAG seven-pass G-type receptor 3(CELSR3) |
| **CEP350** | NM_014810.3 | centrosomal protein 350(CEP350) |
| **CFL2** | NM_138638.1 | cofilin 2(CFL2) |
| **CHFR** | NM_018223.1 | checkpoint with forkhead and ring finger domains(CHFR) |
| **CHKA** | NM_212469.1 | choline kinase alpha(CHKA) |
| **CLCC1** | NM_015127.3 | chloride channel CLIC like 1(CLCC1) |
| **CLRN1** | NM_052995.2 | clarin 1(CLRN1) |
| **COMT** | NM_007310.1 | catechol-O-methyltransferase(COMT) |
| **CORO2A** | NM_003389.2 | coronin 2A(CORO2A) |
| **COTL1** | NM_021149.2 | coactosin like F-actin binding protein 1(COTL1) |
| **CPEB4** | NM_030627.1 | cytoplasmic polyadenylation element binding protein 4(CPEB4) |
| **CRCP** | NM_014478.4 | CGRP receptor component(CRCP) |
| **CSNK1A1** | NM_001025105.1 | casein kinase 1 alpha 1(CSNK1A1) |
| **CTDSPL2** | NM_016396.1 | CTD small phosphatase like 2(CTDSPL2) |
| **DAGLA** | NM_006133.1 | diacylglycerol lipase alpha(DAGLA) |
| **DCP2** | NM_152624.4 | decapping mRNA 2(DCP2) |
| **DCTN4** | NM_016221.2 | dynactin subunit 4(DCTN4) |
| **DDAH1** | NM_012137.2 | dimethylarginine dimethylaminohydrolase 1(DDAH1) |
| **DDIT4** | NM_019058.2 | DNA damage inducible transcript 4(DDIT4) |
| **DDX46** | NM_014829.2 | DEAD-box helicase 46(DDX46) |
| **DENND1B** | NM_144977.2 | DENN domain containing 1B(DENND1B) |
| **DENND5B** | NM_144973.3 | DENN domain containing 5B(DENND5B) |
| **DGKD** | NM_003648.2 | diacylglycerol kinase delta(DGKD) |
| **DHX40** | NM_024612.3 | DEAH-box helicase 40(DHX40) |
| **DIO2** | NM_001007023.2 | deiodinase, iodothyronine type II(DIO2) |
| **DIP2B** | NM_173602.2 | disco interacting protein 2 homolog B(DIP2B) |
| **DIP2C** | NM_014974.1 | disco interacting protein 2 homolog C(DIP2C) |
| **DLG5** | NM_004747.3 | discs large MAGUK scaffold protein 5(DLG5) |
| **DLGAP4** | NM_183006.2 | DLG associated protein 4(DLGAP4) |
| **DMD** | NM_004006.1 | dystrophin(DMD) |
| **DMXL2** | NM_015263.2 | Dmx like 2(DMXL2) |
| **DNAJC13** | NM_015268.3 | DnaJ heat shock protein family (Hsp40) member C13(DNAJC13) |
| **DOCK7** | NM_033407.2 | dedicator of cytokinesis 7(DOCK7) |
| **DOK5** | NM_018431.3 | docking protein 5(DOK5) |
| **DPY19L1** | NM_015283.1 | dpy-19 like 1(DPY19L1) |
| **DPY19L3** | NM_207325.1 | dpy-19 like 3 (C. elegans)(DPY19L3) |
| **DPYSL2** | NM_001386.4 | dihydropyrimidinase like 2(DPYSL2) |
| **DSEL** | NM_032160.2 | dermatan sulfate epimerase-like(DSEL) |
| **E2F3** | NM_001949.2 | E2F transcription factor 3(E2F3) |
| **EAF1** | NM_033083.6 | ELL associated factor 1(EAF1) |
| **EDC3** | NM_025083.2 | enhancer of mRNA decapping 3(EDC3) |
| **EDEM3** | NM_025191.2 | ER degradation enhancing alpha-mannosidase like protein 3(EDEM3) |
| **EDNRA** | NM_001957.1 | endothelin receptor type A(EDNRA) |
| **EEA1** | NM_003566.2 | early endosome antigen 1(EEA1) |
| **EFR3A** | NM_015137.3 | EFR3 homolog A(EFR3A) |
| **EGR3** | NM_004430.2 | early growth response 3(EGR3) |
| **EIF5A2** | NM_020390.5 | eukaryotic translation initiation factor 5A2(EIF5A2) |
| **ELL2** | NM_012081.4 | elongation factor for RNA polymerase II 2(ELL2) |
| **ELOVL5** | NM_021814.3 | ELOVL fatty acid elongase 5(ELOVL5) |
| **EML4** | NM_019063.2 | echinoderm microtubule associated protein like 4(EML4) |
| **ENAH** | NM_018212.4 | enabled homolog (Drosophila)(ENAH) |
| **EPB41L5** | NM_020909.2 | erythrocyte membrane protein band 4.1 like 5(EPB41L5) |
| **EPDR1** | NM_017549.3 | ependymin related 1(EPDR1) |
| **EPHB2** | NM_017449.3 | EPH receptor B2(EPHB2) |
| **EPN2** | NM_014964.3 | epsin 2(EPN2) |
| **ERAP1** | NM_016442.3 | endoplasmic reticulum aminopeptidase 1(ERAP1) |
| **ERMAP** | NM_001017922.1 | erythroblast membrane associated protein (Scianna blood group)(ERMAP) |
| **ERRFI1** | NM_018948.2 | ERBB receptor feedback inhibitor 1(ERRFI1) |
| **EVI5** | NM_005665.4 | ecotropic viral integration site 5(EVI5) |
| **FAF2** | NM_014613.2 | Fas associated factor family member 2(FAF2) |
| **FAM109A** | NM_144671.3 | family with sequence similarity 109 member A(FAM109A) |
| **FAM126B** | NM_173822.2 | family with sequence similarity 126 member B(FAM126B) |
| **FAM131B** | NM_001031690.1 | family with sequence similarity 131 member B(FAM131B) |
| **FAM13A** | NM_001015045.1 | family with sequence similarity 13 member A(FAM13A) |
| **FAM160B1** | NM_001135051.1 | family with sequence similarity 160 member B1(FAM160B1) |
| **FAM168B** | NM_001009993.2 | family with sequence similarity 168 member B(FAM168B) |
| **FAM179B** | NM_015091.2 | family with sequence similarity 179 member B(FAM179B) |
| **FAM43A** | NM_153690.4 | family with sequence similarity 43 member A(FAM43A) |
| **FAM46C** | NM_017709.3 | family with sequence similarity 46 member C(FAM46C) |
| **FAM73B** | NM_032809.2 | #N/A |
| **FBXL17** | NM_022824.1 | F-box and leucine rich repeat protein 17(FBXL17) |
| **FBXL20** | NM_032875.1 | F-box and leucine rich repeat protein 20(FBXL20) |
| **FBXO32** | NM_058229.2 | F-box protein 32(FBXO32) |
| **FBXO34** | NM_017943.2 | F-box protein 34(FBXO34) |
| **FKBP3** | NM_002013.2 | FK506 binding protein 3(FKBP3) |
| **FLVCR1** | NM_014053.1 | feline leukemia virus subgroup C cellular receptor 1(FLVCR1) |
| **FLVCR2** | NM_017791.1 | feline leukemia virus subgroup C cellular receptor family member 2(FLVCR2) |
| **FOXD1** | NM_004472.2 | forkhead box D1(FOXD1) |
| **FXR1** | NM_005087.2 | FMR1 autosomal homolog 1(FXR1) |
| **FYCO1** | NM_024513.1 | FYVE and coiled-coil domain containing 1(FYCO1) |
| **FZD2** | NM_001466.2 | frizzled class receptor 2(FZD2) |
| **GALNT2** | NM_004481.2 | polypeptide N-acetylgalactosaminyltransferase 2(GALNT2) |
| **GCNT2** | NM_145655.3 | glucosaminyl (N-acetyl) transferase 2, I-branching enzyme (I blood group)(GCNT2) |
| **GIGYF2** | NM_015575.3 | GRB10 interacting GYF protein 2(GIGYF2) |
| **GLCCI1** | NM_138426.2 | glucocorticoid induced 1(GLCCI1) |
| **GLCE** | NM_015554.1 | glucuronic acid epimerase(GLCE) |
| **GLUD1** | NM_005271.1 | glutamate dehydrogenase 1(GLUD1) |
| **GNAI2** | NM_002070.2 | G protein subunit alpha i2(GNAI2) |
| **GNG10** | NM_001017998.2 | G protein subunit gamma 10(GNG10) |
| **GNPDA1** | NM_005471.3 | glucosamine-6-phosphate deaminase 1(GNPDA1) |
| **GOLGA4** | NM_002078.3 | golgin A4(GOLGA4) |
| **GOSR2** | NM_004287.3 | golgi SNAP receptor complex member 2(GOSR2) |
| **GOT2** | NM_002080.2 | glutamic-oxaloacetic transaminase 2(GOT2) |
| **GPR108** | NM_001080452.1 | G protein-coupled receptor 108(GPR108) |
| **GPR124** | NM_032777.6 | #N/A |
| **HCFC2** | NM_013320.1 | host cell factor C2(HCFC2) |
| **HDAC5** | NM_005474.4 | histone deacetylase 5(HDAC5) |
| **HDAC9** | NM_178425.2 | histone deacetylase 9(HDAC9) |
| **HMGB3** | NM_005342.2 | high mobility group box 3(HMGB3) |
| **HNRNPC** | NM_001077442.1 | heterogeneous nuclear ribonucleoprotein C (C1/C2)(HNRNPC) |
| **HOXA11** | NM_005523.5 | homeobox A11(HOXA11) |
| **HPS4** | NM_152842.1 | HPS4, biogenesis of lysosomal organelles complex 3 subunit 2(HPS4) |
| **HSPA5** | NM_005347.2 | heat shock protein family A (Hsp70) member 5(HSPA5) |
| **IFFO2** | NM_001136265.1 | intermediate filament family orphan 2(IFFO2) |
| **IFNAR2** | NM_207584.1 | interferon alpha and beta receptor subunit 2(IFNAR2) |
| **IGF1R** | NM_000875.2 | insulin like growth factor 1 receptor(IGF1R) |
| **IGF2R** | NM_000876.2 | insulin like growth factor 2 receptor(IGF2R) |
| **IKZF2** | NM_016260.2 | IKAROS family zinc finger 2(IKZF2) |
| **INO80D** | NM_017759.4 | INO80 complex subunit D(INO80D) |
| **IP6K3** | NM_001142883.1 | inositol hexakisphosphate kinase 3(IP6K3) |
| **IRS2** | NM_003749.2 | insulin receptor substrate 2(IRS2) |
| **ITGA4** | NM_000885.4 | integrin subunit alpha 4(ITGA4) |
| **ITGA5** | NM_002205.2 | integrin subunit alpha 5(ITGA5) |
| **ITGB3** | NM_000212.2 | integrin subunit beta 3(ITGB3) |
| **ITSN1** | NM_003024.2 | intersectin 1(ITSN1) |
| **JAK1** | NM_002227.2 | Janus kinase 1(JAK1) |
| **JARID2** | NM_004973.2 | jumonji and AT-rich interaction domain containing 2(JARID2) |
| **JDP2** | NM_130469.2 | Jun dimerization protein 2(JDP2) |
| **JOSD1** | NM_014876.3 | Josephin domain containing 1(JOSD1) |
| **KCTD5** | NM_018992.2 | potassium channel tetramerization domain containing 5(KCTD5) |
| **KIAA0247** | NM_014734.2 | #N/A |
| **KIAA0408** | NM_014702.3 | KIAA0408(KIAA0408) |
| **KIAA1033** | NM_015275.1 | KIAA1033(KIAA1033) |
| **KIF21B** | XM_935437.1 | kinesin family member 21B(KIF21B) |
| **KLF10** | NM_005655.1 | Kruppel like factor 10(KLF10) |
| **KLHL20** | NM_014458.3 | kelch like family member 20(KLHL20) |
| **KLHL28** | NM_017658.3 | kelch like family member 28(KLHL28) |
| **KRAS** | NM_033360.2 | KRAS proto-oncogene, GTPase(KRAS) |
| **LARGE** | NM_004737.3 | #N/A |
| **LARP1** | NM_033551.2 | La ribonucleoprotein domain family member 1(LARP1) |
| **LARP4** | NM_199188.1 | La ribonucleoprotein domain family member 4(LARP4) |
| **LATS2** | NM_014572.2 | large tumor suppressor kinase 2(LATS2) |
| **LCOR** | NM_032440.2 | ligand dependent nuclear receptor corepressor(LCOR) |
| **LDLR** | NM_000527.2 | low density lipoprotein receptor(LDLR) |
| **LHFPL2** | NM_005779.1 | lipoma HMGIC fusion partner-like 2(LHFPL2) |
| **LIFR** | NM_002310.3 | leukemia inhibitory factor receptor alpha(LIFR) |
| **LIN7C** | NM_018362.2 | lin-7 homolog C, crumbs cell polarity complex component(LIN7C) |
| **LMBR1** | NM_022458.3 | limb development membrane protein 1(LMBR1) |
| **LOX** | NM_002317.3 | lysyl oxidase(LOX) |
| **LPGAT1** | NM_014873.1 | lysophosphatidylglycerol acyltransferase 1(LPGAT1) |
| **LPP** | NM_005578.2 | LIM domain containing preferred translocation partner in lipoma(LPP) |
| **LRCH2** | NM_020871.3 | leucine rich repeats and calponin homology domain containing 2(LRCH2) |
| **LRRC8D** | NM_018103.3 | leucine rich repeat containing 8 family member D(LRRC8D) |
| **LYSMD3** | NM_198273.1 | LysM domain containing 3(LYSMD3) |
| **LYST** | NM_001005736.1 | lysosomal trafficking regulator(LYST) |
| **MAF** | NM_005360.3 | MAF bZIP transcription factor(MAF) |
| **MAL** | NM_002371.2 | mal, T-cell differentiation protein(MAL) |
| **MAML1** | NM_014757.3 | mastermind like transcriptional coactivator 1(MAML1) |
| **MAN1A2** | NM_006699.3 | mannosidase alpha class 1A member 2(MAN1A2) |
| **MAN1B1** | NM_016219.2 | mannosidase alpha class 1B member 1(MAN1B1) |
| **MAP3K1** | NM_005921.1 | mitogen-activated protein kinase kinase kinase 1(MAP3K1) |
| **MAP3K12** | NM_006301.2 | mitogen-activated protein kinase kinase kinase 12(MAP3K12) |
| **MAP3K5** | NM_005923.3 | mitogen-activated protein kinase kinase kinase 5(MAP3K5) |
| **MAP4K4** | NM_145686.2 | mitogen-activated protein kinase kinase kinase kinase 4(MAP4K4) |
| **MARCKS** | NM_002356.5 | myristoylated alanine rich protein kinase C substrate(MARCKS) |
| **MAST3** | NM_015016.1 | microtubule associated serine/threonine kinase 3(MAST3) |
| **MAT2A** | NM_005911.4 | methionine adenosyltransferase 2A(MAT2A) |
| **MATR3** | NM_018834.4 | matrin 3(MATR3) |
| **MBD6** | NM_052897.3 | methyl-CpG binding domain protein 6(MBD6) |
| **MBNL1** | NM_207293.1 | muscleblind like splicing regulator 1(MBNL1) |
| **MBNL2** | NM_144778.2 | muscleblind like splicing regulator 2(MBNL2) |
| **MEF2D** | NM_005920.2 | myocyte enhancer factor 2D(MEF2D) |
| **MEIS2** | NM_002399.2 | Meis homeobox 2(MEIS2) |
| **MEX3B** | NM_032246.3 | mex-3 RNA binding family member B(MEX3B) |
| **MFSD11** | NM_024311.2 | major facilitator superfamily domain containing 11(MFSD11) |
| **MFSD6** | NM_017694.3 | major facilitator superfamily domain containing 6(MFSD6) |
| **MIB1** | NM_020774.2 | mindbomb E3 ubiquitin protein ligase 1(MIB1) |
| **MICAL1** | NM_022765.2 | microtubule associated monooxygenase, calponin and LIM domain containing 1(MICAL1) |
| **MIER2** | NM_017550.1 | MIER family member 2(MIER2) |
| **MIER3** | NM_152622.3 | MIER family member 3(MIER3) |
| **MINPP1** | NM_004897.2 | multiple inositol-polyphosphate phosphatase 1(MINPP1) |
| **MKL2** | NM_014048.3 | MKL1/myocardin like 2(MKL2) |
| **MNT** | NM_020310.2 | MAX network transcriptional repressor(MNT) |
| **MOV10** | NM_020963.2 | Mov10 RISC complex RNA helicase(MOV10) |
| **MPL** | NM_005373.1 | MPL proto-oncogene, thrombopoietin receptor(MPL) |
| **MXRA5** | NM_015419.2 | matrix remodeling associated 5(MXRA5) |
| **MYO5A** | NM_000259.2 | myosin VA(MYO5A) |
| **NADK** | NM_023018.3 | NAD kinase(NADK) |
| **NAGPA** | NM_016256.2 | N-acetylglucosamine-1-phosphodiester alpha-N-acetylglucosaminidase(NAGPA) |
| **NAP1L1** | NM_139207.1 | nucleosome assembly protein 1 like 1(NAP1L1) |
| **NAPG** | NM_003826.1 | NSF attachment protein gamma(NAPG) |
| **NCALD** | NM_032041.1 | neurocalcin delta(NCALD) |
| **NCOA3** | NM_181659.1 | nuclear receptor coactivator 3(NCOA3) |
| **NCOR2** | NM_001077261.1 | nuclear receptor corepressor 2(NCOR2) |
| **NEFM** | NM_005382.1 | neurofilament, medium polypeptide(NEFM) |
| **NEURL1B** | NM_001142651.1 | neuralized E3 ubiquitin protein ligase 1B(NEURL1B) |
| **NF1** | NM_001042492.1 | neurofibromin 1(NF1) |
| **NFAT5** | NM_173215.1 | nuclear factor of activated T-cells 5(NFAT5) |
| **NFATC3** | NM_173164.1 | nuclear factor of activated T-cells 3(NFATC3) |
| **NFIA** | NM_005595.1 | nuclear factor I A(NFIA) |
| **NFIB** | NM_005596.2 | nuclear factor I B(NFIB) |
| **NID1** | NM_002508.2 | nidogen 1(NID1) |
| **NOTCH1** | NM_017617.3 | notch 1(NOTCH1) |
| **NPAS2** | NM_002518.3 | neuronal PAS domain protein 2(NPAS2) |
| **NR4A2** | NM_006186.2 | nuclear receptor subfamily 4 group A member 2(NR4A2) |
| **NRK** | NM_198465.2 | Nik related kinase(NRK) |
| **NRP2** | NM_201266.1 | neuropilin 2(NRP2) |
| **NSD1** | NM_172349.1 | nuclear receptor binding SET domain protein 1(NSD1) |
| **NT5E** | NM_002526.1 | 5'-nucleotidase ecto(NT5E) |
| **NUAK1** | NM_014840.2 | NUAK family kinase 1(NUAK1) |
| **OSBPL8** | NM_001003712.1 | oxysterol binding protein like 8(OSBPL8) |
| **OTUD6B** | NM_016023.2 | OTU domain containing 6B(OTUD6B) |
| **OXR1** | NM_181354.3 | oxidation resistance 1(OXR1) |
| **P4HA2** | NM_001017973.1 | prolyl 4-hydroxylase subunit alpha 2(P4HA2) |
| **PAAF1** | NM_025155.1 | proteasomal ATPase associated factor 1(PAAF1) |
| **PAFAH1B2** | NM_002572.2 | platelet activating factor acetylhydrolase 1b catalytic subunit 2(PAFAH1B2) |
| **PAPOLA** | NM_032632.3 | poly(A) polymerase alpha(PAPOLA) |
| **PARP16** | NM_017851.4 | poly(ADP-ribose) polymerase family member 16(PARP16) |
| **PAWR** | NM_002583.2 | pro-apoptotic WT1 regulator(PAWR) |
| **PBRM1** | NM_181042.2 | polybromo 1(PBRM1) |
| **PCDH10** | NM_032961.1 | protocadherin 10(PCDH10) |
| **PDE5A** | NM_033430.2 | phosphodiesterase 5A(PDE5A) |
| **PDGFRB** | NM_002609.3 | platelet derived growth factor receptor beta(PDGFRB) |
| **PDSS1** | NM_014317.3 | prenyl (decaprenyl) diphosphate synthase, subunit 1(PDSS1) |
| **PDSS2** | NM_020381.2 | prenyl (decaprenyl) diphosphate synthase, subunit 2(PDSS2) |
| **PGM1** | NM_002633.2 | phosphoglucomutase 1(PGM1) |
| **PGP** | NM_001042371.2 | phosphoglycolate phosphatase(PGP) |
| **PHACTR2** | NM_001100164.1 | phosphatase and actin regulator 2(PHACTR2) |
| **PHF13** | NM_153812.1 | PHD finger protein 13(PHF13) |
| **PHIP** | NM_017934.4 | pleckstrin homology domain interacting protein(PHIP) |
| **PHTF2** | NM_020432.2 | putative homeodomain transcription factor 2(PHTF2) |
| **PI4K2B** | NM_018323.2 | phosphatidylinositol 4-kinase type 2 beta(PI4K2B) |
| **PIGA** | NM_020473.2 | phosphatidylinositol glycan anchor biosynthesis class A(PIGA) |
| **PIGX** | NM_017861.1 | phosphatidylinositol glycan anchor biosynthesis class X(PIGX) |
| **PIK3CD** | NM_005026.2 | phosphatidylinositol-4,5-bisphosphate 3-kinase catalytic subunit delta(PIK3CD) |
| **PIK3R2** | NM_005027.2 | phosphoinositide-3-kinase regulatory subunit 2(PIK3R2) |
| **PIP4K2B** | NM_003559.4 | phosphatidylinositol-5-phosphate 4-kinase type 2 beta(PIP4K2B) |
| **PITX1** | NM_002653.3 | paired like homeodomain 1(PITX1) |
| **PKNOX2** | NM_022062.2 | PBX/knotted 1 homeobox 2(PKNOX2) |
| **PLCXD3** | NM_001005473.1 | phosphatidylinositol specific phospholipase C X domain containing 3(PLCXD3) |
| **PLEKHO2** | NM_025201.3 | pleckstrin homology domain containing O2(PLEKHO2) |
| **PLXNA1** | NM_032242.2 | plexin A1(PLXNA1) |
| **PNKD** | NM_022572.3 | paroxysmal nonkinesigenic dyskinesia(PNKD) |
| **PPFIA1** | NM_177423.1 | PTPRF interacting protein alpha 1(PPFIA1) |
| **PPP1R12A** | NM_002480.1 | protein phosphatase 1 regulatory subunit 12A(PPP1R12A) |
| **PPP1R14C** | NM_030949.2 | protein phosphatase 1 regulatory inhibitor subunit 14C(PPP1R14C) |
| **PPP1R2** | NM_006241.3 | protein phosphatase 1 regulatory inhibitor subunit 2(PPP1R2) |
| **PPP3R1** | NM_000945.3 | protein phosphatase 3 regulatory subunit B, alpha(PPP3R1) |
| **PPTC7** | NM_139283.1 | PTC7 protein phosphatase homolog(PPTC7) |
| **PROM1** | NM_006017.1 | prominin 1(PROM1) |
| **PRRG1** | NM_000950.1 | proline rich and Gla domain 1(PRRG1) |
| **PRUNE2** | NM_138818.2 | prune homolog 2(PRUNE2) |
| **PSD3** | NM_015310.3 | pleckstrin and Sec7 domain containing 3(PSD3) |
| **PTGFRN** | NM_020440.2 | prostaglandin F2 receptor inhibitor(PTGFRN) |
| **PTP4A1** | NM_003463.3 | protein tyrosine phosphatase type IVA, member 1(PTP4A1) |
| **PTPDC1** | NM_152422.3 | protein tyrosine phosphatase domain containing 1(PTPDC1) |
| **PTPN13** | NM_080684.1 | protein tyrosine phosphatase, non-receptor type 13(PTPN13) |
| **PTPRK** | NM_002844.2 | protein tyrosine phosphatase, receptor type K(PTPRK) |
| **PXK** | NM_017771.3 | PX domain containing serine/threonine kinase like(PXK) |
| **RAB22A** | NM_020673.2 | RAB22A, member RAS oncogene family(RAB22A) |
| **RAB27B** | NM_004163.3 | RAB27B, member RAS oncogene family(RAB27B) |
| **RAD23B** | NM_002874.3 | RAD23 homolog B, nucleotide excision repair protein(RAD23B) |
| **RAI14** | NM_015577.1 | retinoic acid induced 14(RAI14) |
| **RALGAPB** | NM_020336.2 | Ral GTPase activating protein non-catalytic beta subunit(RALGAPB) |
| **RANBP10** | NM_020850.1 | RAN binding protein 10(RANBP10) |
| **RAP1B** | NM_015646.4 | RAP1B, member of RAS oncogene family(RAP1B) |
| **RAP2C** | NM_021183.3 | RAP2C, member of RAS oncogene family(RAP2C) |
| **RAPGEF2** | NM_014247.2 | Rap guanine nucleotide exchange factor 2(RAPGEF2) |
| **RAPH1** | NM_203365.2 | Ras association (RalGDS/AF-6) and pleckstrin homology domains 1(RAPH1) |
| **RARB** | NM_000965.2 | retinoic acid receptor beta(RARB) |
| **RASAL2** | NM_004841.2 | RAS protein activator like 2(RASAL2) |
| **RASD1** | NM_016084.3 | ras related dexamethasone induced 1(RASD1) |
| **RASSF4** | NM_032023.3 | Ras association domain family member 4(RASSF4) |
| **RAVER2** | NM_018211.2 | ribonucleoprotein, PTB binding 2(RAVER2) |
| **RBM12** | NM_006047.4 | RNA binding motif protein 12(RBM12) |
| **RBM15B** | NM_013286.3 | RNA binding motif protein 15B(RBM15B) |
| **RCOR3** | NM_018254.2 | REST corepressor 3(RCOR3) |
| **RECK** | NM_021111.1 | reversion inducing cysteine rich protein with kazal motifs(RECK) |
| **RELL1** | NM_001085399.1 | RELT like 1(RELL1) |
| **RFTN2** | NM_144629.1 | raftlin family member 2(RFTN2) |
| **RFX7** | NM_022841.5 | regulatory factor X7(RFX7) |
| **RGL1** | NM_015149.3 | ral guanine nucleotide dissociation stimulator like 1(RGL1) |
| **RGS2** | NM_002923.1 | regulator of G-protein signaling 2(RGS2) |
| **RIOK3** | NM_003831.3 | RIO kinase 3(RIOK3) |
| **RNF169** | NM_001098638.1 | ring finger protein 169(RNF169) |
| **RNF170** | NM_030954.2 | ring finger protein 170(RNF170) |
| **RNF44** | NM_014901.4 | ring finger protein 44(RNF44) |
| **RNMT** | NM_003799.1 | RNA guanine-7 methyltransferase(RNMT) |
| **RPRD1A** | NM_018170.3 | regulation of nuclear pre-mRNA domain containing 1A(RPRD1A) |
| **RPRD2** | NM_015203.3 | regulation of nuclear pre-mRNA domain containing 2(RPRD2) |
| **RPS6KA2** | NM_001006932.1 | ribosomal protein S6 kinase A2(RPS6KA2) |
| **RTN4R** | NM_023004.5 | reticulon 4 receptor(RTN4R) |
| **RUNX1** | NM_001001890.1 | runt related transcription factor 1(RUNX1) |
| **SACS** | NM_014363.3 | sacsin molecular chaperone(SACS) |
| **SAMD4A** | NM_015589.3 | sterile alpha motif domain containing 4A(SAMD4A) |
| **SATB1** | NM_002971.2 | SATB homeobox 1(SATB1) |
| **SBF1** | NM_002972.1 | SET binding factor 1(SBF1) |
| **SCAMP1** | NM_004866.4 | secretory carrier membrane protein 1(SCAMP1) |
| **SCN9A** | NM_002977.2 | sodium voltage-gated channel alpha subunit 9(SCN9A) |
| **SCYL3** | NM_020423.4 | SCY1 like pseudokinase 3(SCYL3) |
| **SDAD1** | NM_018115.2 | SDA1 domain containing 1(SDAD1) |
| **SEC23IP** | NM_007190.2 | SEC23 interacting protein(SEC23IP) |
| **SEC24A** | NM_021982.1 | SEC24 homolog A, COPII coat complex component(SEC24A) |
| **SEC62** | NM_003262.3 | SEC62 homolog, preprotein translocation factor(SEC62) |
| **SEL1L3** | NM_015187.3 | SEL1L family member 3(SEL1L3) |
| **SEMA6B** | NM_032108.2 | semaphorin 6B(SEMA6B) |
| **SENP5** | NM_152699.3 | SUMO1/sentrin specific peptidase 5(SENP5) |
| **SERPINE1** | NM_000602.1 | serpin family E member 1(SERPINE1) |
| **SFXN1** | NM_022754.4 | sideroflexin 1(SFXN1) |
| **SGCB** | NM_000232.3 | sarcoglycan beta(SGCB) |
| **SGMS2** | NM_152621.4 | sphingomyelin synthase 2(SGMS2) |
| **SH3PXD2A** | NM_014631.2 | SH3 and PX domains 2A(SH3PXD2A) |
| **SH3RF1** | NM_020870.3 | SH3 domain containing ring finger 1(SH3RF1) |
| **SHOC2** | NM_007373.2 | SHOC2, leucine rich repeat scaffold protein(SHOC2) |
| **SIDT2** | NM_001040455.1 | SID1 transmembrane family member 2(SIDT2) |
| **SIK3** | NM_025164.3 | SIK family kinase 3(SIK3) |
| **SIX4** | NM_017420.3 | SIX homeobox 4(SIX4) |
| **SLC12A6** | NM_001042496.1 | solute carrier family 12 member 6(SLC12A6) |
| **SLC22A23** | NM_015482.1 | solute carrier family 22 member 23(SLC22A23) |
| **SLC22A5** | NM_003060.2 | solute carrier family 22 member 5(SLC22A5) |
| **SLC29A3** | NM_018344.3 | solute carrier family 29 member 3(SLC29A3) |
| **SLC36A1** | NM_078483.2 | solute carrier family 36 member 1(SLC36A1) |
| **SLC38A1** | NM_030674.3 | solute carrier family 38 member 1(SLC38A1) |
| **SLC38A7** | NM_018231.1 | solute carrier family 38 member 7(SLC38A7) |
| **SLC39A10** | NM_020342.1 | solute carrier family 39 member 10(SLC39A10) |
| **SLC41A2** | NM_032148.2 | solute carrier family 41 member 2(SLC41A2) |
| **SLC4A7** | NM_003615.3 | solute carrier family 4 member 7(SLC4A7) |
| **SLC6A9** | NM_001024845.1 | solute carrier family 6 member 9(SLC6A9) |
| **SLC7A6** | NM_001076785.1 | solute carrier family 7 member 6(SLC7A6) |
| **SLC9A8** | NM_015266.1 | solute carrier family 9 member A8(SLC9A8) |
| **SMAD2** | NM_001003652.2 | SMAD family member 2(SMAD2) |
| **SMARCD2** | NM_003077.2 | SWI/SNF related, matrix associated, actin dependent regulator of chromatin, subfamily d, member 2(SMARCD2) |
| **SNTB2** | NM_006750.3 | syntrophin beta 2(SNTB2) |
| **SNX1** | NM_003099.3 | sorting nexin 1(SNX1) |
| **SNX16** | NM_022133.2 | sorting nexin 16(SNX16) |
| **SNX27** | NM_030918.5 | sorting nexin family member 27(SNX27) |
| **SNX30** | NM_001012994.1 | sorting nexin family member 30(SNX30) |
| **SNX6** | NM_021249.3 | sorting nexin 6(SNX6) |
| **SOCS1** | NM_003745.1 | suppressor of cytokine signaling 1(SOCS1) |
| **SOCS3** | NM_003955.3 | suppressor of cytokine signaling 3(SOCS3) |
| **SON** | NM_032195.1 | SON DNA binding protein(SON) |
| **SOS1** | NM_005633.2 | SOS Ras/Rac guanine nucleotide exchange factor 1(SOS1) |
| **SOX13** | NM_005686.2 | SRY-box 13(SOX13) |
| **SOX4** | NM_003107.2 | SRY-box 4(SOX4) |
| **SOX9** | NM_000346.2 | SRY-box 9(SOX9) |
| **SP4** | NM_003112.3 | Sp4 transcription factor(SP4) |
| **SPAG9** | NM_003971.3 | sperm associated antigen 9(SPAG9) |
| **SSBP2** | NM_012446.2 | single stranded DNA binding protein 2(SSBP2) |
| **SSH2** | NM_033389.2 | slingshot protein phosphatase 2(SSH2) |
| **STARD3** | NM_006804.2 | StAR related lipid transfer domain containing 3(STARD3) |
| **STIM2** | NM_020860.1 | stromal interaction molecule 2(STIM2) |
| **STOML1** | NM_004809.3 | stomatin like 1(STOML1) |
| **SUCLG2** | NM_003848.1 | succinate-CoA ligase GDP-forming beta subunit(SUCLG2) |
| **SUPT3H** | NM_181356.1 | SPT3 homolog, SAGA and STAGA complex component(SUPT3H) |
| **SUV39H2** | NM_024670.3 | suppressor of variegation 3-9 homolog 2(SUV39H2) |
| **SYN2** | NM_003178.4 | synapsin II(SYN2) |
| **TBC1D10B** | NM_015527.2 | TBC1 domain family member 10B(TBC1D10B) |
| **TCP11L1** | NM_018393.2 | t-complex 11 like 1(TCP11L1) |
| **TIA1** | NM_022037.1 | TIA1 cytotoxic granule associated RNA binding protein(TIA1) |
| **TMEM110** | NM_198563.1 | transmembrane protein 110(TMEM110) |
| **TMEM135** | NM_022918.2 | transmembrane protein 135(TMEM135) |
| **TMTC3** | NM_181783.2 | transmembrane and tetratricopeptide repeat containing 3(TMTC3) |
| **TNPO3** | NM_012470.2 | transportin 3(TNPO3) |
| **TRERF1** | NM_033502.1 | transcriptional regulating factor 1(TRERF1) |
| **TRIM9** | NM_015163.4 | tripartite motif containing 9(TRIM9) |
| **TRPA1** | NM_007332.2 | transient receptor potential cation channel subfamily A member 1(TRPA1) |
| **TRPS1** | NM_014112.2 | transcriptional repressor GATA binding 1(TRPS1) |
| **TSC1** | NM_001008567.1 | tuberous sclerosis 1(TSC1) |
| **TSPAN33** | NM_178562.2 | tetraspanin 33(TSPAN33) |
| **TTLL7** | NM_024686.4 | tubulin tyrosine ligase like 7(TTLL7) |
| **TUSC3** | NM_006765.2 | tumor suppressor candidate 3(TUSC3) |
| **UBAC1** | NM_016172.2 | UBA domain containing 1(UBAC1) |
| **UBE2D2** | NM_181838.1 | ubiquitin conjugating enzyme E2 D2(UBE2D2) |
| **UBE2F** | NM_080678.1 | ubiquitin conjugating enzyme E2 F (putative)(UBE2F) |
| **UBE2I** | NM_194260.1 | ubiquitin conjugating enzyme E2 I(UBE2I) |
| **UBE2O** | NM_022066.2 | ubiquitin conjugating enzyme E2 O(UBE2O) |
| **VAT1** | NM_006373.3 | vesicle amine transport 1(VAT1) |
| **VAT1L** | NM_020927.1 | vesicle amine transport 1 like(VAT1L) |

**Supplementary Table 4. Upregulated genes in HMCs treated with anti-dsDNA antibodies that contain a target binding site for let-7a.**

| **ID** | **RefSeq** | **Gene Name** |
| --- | --- | --- |
| **ABCC5** | NM_001023587.1 | ATP binding cassette subfamily C member 5(ABCC5) |
| **ABT1** | NM_013375.2 | activator of basal transcription 1(ABT1) |
| **ACER3** | NM_018367.5 | alkaline ceramidase 3(ACER3) |
| **ACTR10** | NM_018477.2 | actin-related protein 10 homolog(ACTR10) |
| **ACVR1B** | NM_020328.2 | activin A receptor type 1B(ACVR1B) |
| **ACVR2A** | NM_001616.3 | activin A receptor type 2A(ACVR2A) |
| **ADAMTS1** | NM_006988.3 | ADAM metallopeptidase with thrombospondin type 1 motif 1(ADAMTS1) |
| **ADCY9** | NM_001116.2 | adenylate cyclase 9(ADCY9) |
| **ADIPOR2** | NM_024551.2 | adiponectin receptor 2(ADIPOR2) |
| **AHCTF1** | XM_942402.1 | AT-hook containing transcription factor 1(AHCTF1) |
| **ALKBH1** | NM_006020.2 | alkB homolog 1, histone H2A dioxygenase(ALKBH1) |
| **AMMECR1L** | NM_031445.2 | AMMECR1 like(AMMECR1L) |
| **AMT** | NM_000481.2 | aminomethyltransferase(AMT) |
| **ANKRD12** | NM_015208.3 | ankyrin repeat domain 12(ANKRD12) |
| **ANKRD28** | NM_015199.2 | ankyrin repeat domain 28(ANKRD28) |
| **ANKRD46** | NM_198401.2 | ankyrin repeat domain 46(ANKRD46) |
| **ANKRD49** | NM_017704.2 | ankyrin repeat domain 49(ANKRD49) |
| **ANKRD52** | NM_173595.3 | ankyrin repeat domain 52(ANKRD52) |
| **AP4E1** | NM_007347.3 | adaptor related protein complex 4 epsilon 1 subunit(AP4E1) |
| **APPBP2** | NM_006380.2 | amyloid beta precursor protein binding protein 2(APPBP2) |
| **ARHGEF7** | NM_003899.2 | Rho guanine nucleotide exchange factor 7(ARHGEF7) |
| **ARID3A** | NM_005224.2 | AT-rich interaction domain 3A(ARID3A) |
| **ARID3B** | NM_006465.2 | AT-rich interaction domain 3B(ARID3B) |
| **ARL5A** | NM_001037174.1 | ADP ribosylation factor like GTPase 5A(ARL5A) |
| **ARL6IP6** | NM_152522.3 | ADP ribosylation factor like GTPase 6 interacting protein 6(ARL6IP6) |
| **ARMC8** | NM_213654.1 | armadillo repeat containing 8(ARMC8) |
| **ARPP19** | NM_006628.4 | cAMP regulated phosphoprotein 19(ARPP19) |
| **ARRDC4** | NM_183376.1 | arrestin domain containing 4(ARRDC4) |
| **ASPHD2** | NM_020437.3 | aspartate beta-hydroxylase domain containing 2(ASPHD2) |
| **ATG10** | NM_031482.3 | autophagy related 10(ATG10) |
| **ATP2A2** | NM_170665.2 | ATPase sarcoplasmic/endoplasmic reticulum Ca2+ transporting 2(ATP2A2) |
| **ATP8B4** | NM_024837.2 | ATPase phospholipid transporting 8B4 (putative)(ATP8B4) |
| **ATPAF1** | NM_022745.3 | ATP synthase mitochondrial F1 complex assembly factor 1(ATPAF1) |
| **ATXN1L** | NM_001137675.2 | ataxin 1 like(ATXN1L) |
| **ATXN7L2** | NM_153340.3 | ataxin 7 like 2(ATXN7L2) |
| **ATXN7L3** | NM_001098833.1 | ataxin 7 like 3(ATXN7L3) |
| **BBX** | NM_020235.3 | BBX, HMG-box containing(BBX) |
| **BCAP29** | NM_001008405.1 | B-cell receptor associated protein 29(BCAP29) |
| **BCAT1** | NM_005504.4 | branched chain amino acid transaminase 1(BCAT1) |
| **BCL2L1** | NM_138578.1 | BCL2 like 1(BCL2L1) |
| **BRWD1** | NM_001007246.1 | bromodomain and WD repeat domain containing 1(BRWD1) |
| **BRWD3** | NM_153252.3 | bromodomain and WD repeat domain containing 3(BRWD3) |
| **BTBD3** | NM_181443.1 | BTB domain containing 3(BTBD3) |
| **BTF3L4** | NM_152265.2 | basic transcription factor 3 like 4(BTF3L4) |
| **C11orf57** | NM_018195.3 | chromosome 11 open reading frame 57(C11orf57) |
| **C14orf28** | NM_001017923.1 | chromosome 14 open reading frame 28(C14orf28) |
| **C15orf39** | NM_015492.4 | chromosome 15 open reading frame 39(C15orf39) |
| **C18orf21** | NM_031446.3 | chromosome 18 open reading frame 21(C18orf21) |
| **C1orf21** | NM_030806.3 | chromosome 1 open reading frame 21(C1orf21) |
| **C3orf52** | NM_024616.1 | chromosome 3 open reading frame 52(C3orf52) |
| **C5orf51** | NM_175921.4 | chromosome 5 open reading frame 51(C5orf51) |
| **CALU** | NM_001219.2 | calumenin(CALU) |
| **CASP3** | NM_004346.3 | caspase 3(CASP3) |
| **CBL** | NM_005188.2 | Cbl proto-oncogene(CBL) |
| **CBX2** | NM_032647.2 | chromobox 2(CBX2) |
| **CBX5** | NM_012117.1 | chromobox 5(CBX5) |
| **CCDC93** | NM_019044.3 | coiled-coil domain containing 93(CCDC93) |
| **CCL7** | NM_006273.2 | C-C motif chemokine ligand 7(CCL7) |
| **CCND1** | NM_053056.2 | cyclin D1(CCND1) |
| **CCND2** | NM_001759.2 | cyclin D2(CCND2) |
| **CCNJ** | NM_019084.2 | cyclin J(CCNJ) |
| **CCNT2** | NM_058241.1 | cyclin T2(CCNT2) |
| **CCNY** | NM_145012.3 | cyclin Y(CCNY) |
| **CD200** | NM_001004196.2 | CD200 molecule(CD200) |
| **CD276** | NM_025240.2 | CD276 molecule(CD276) |
| **CDC14B** | NM_033332.1 | cell division cycle 14B(CDC14B) |
| **CDC25A** | NM_001789.2 | cell division cycle 25A(CDC25A) |
| **CDC34** | NM_004359.1 | cell division cycle 34(CDC34) |
| **CDC42SE1** | NM_001038707.1 | CDC42 small effector 1(CDC42SE1) |
| **CDK6** | NM_001259.5 | cyclin dependent kinase 6(CDK6) |
| **CDV3** | NM_017548.3 | CDV3 homolog(CDV3) |
| **CEBPD** | NM_005195.3 | CCAAT/enhancer binding protein delta(CEBPD) |
| **CEP120** | NM_153223.2 | centrosomal protein 120(CEP120) |
| **CEP135** | NM_025009.3 | centrosomal protein 135(CEP135) |
| **CERCAM** | NM_016174.3 | cerebral endothelial cell adhesion molecule(CERCAM) |
| **CGNL1** | NM_032866.3 | cingulin like 1(CGNL1) |
| **CHD7** | NM_017780.2 | chromodomain helicase DNA binding protein 7(CHD7) |
| **CHD9** | NM_025134.4 | chromodomain helicase DNA binding protein 9(CHD9) |
| **CHST3** | NM_004273.2 | carbohydrate sulfotransferase 3(CHST3) |
| **CHSY3** | NM_175856.4 | chondroitin sulfate synthase 3(CHSY3) |
| **CHUK** | NM_001278.3 | conserved helix-loop-helix ubiquitous kinase(CHUK) |
| **CLDN12** | NM_012129.2 | claudin 12(CLDN12) |
| **CLP1** | NM_006831.1 | cleavage and polyadenylation factor I subunit 1(CLP1) |
| **CMTM6** | NM_017801.2 | CKLF like MARVEL transmembrane domain containing 6(CMTM6) |
| **CNOT2** | NM_014515.4 | CCR4-NOT transcription complex subunit 2(CNOT2) |
| **COIL** | NM_004645.2 | coilin(COIL) |
| **COL11A1** | NM_001854.2 | collagen type XI alpha 1 chain(COL11A1) |
| **COL15A1** | NM_001855.3 | collagen type XV alpha 1 chain(COL15A1) |
| **COL1A1** | NM_000088.3 | collagen type I alpha 1 chain(COL1A1) |
| **COL3A1** | NM_000090.3 | collagen type III alpha 1 chain(COL3A1) |
| **COL5A2** | NM_000393.3 | collagen type V alpha 2 chain(COL5A2) |
| **CPA4** | NM_016352.2 | carboxypeptidase A4(CPA4) |
| **CPEB2** | NM_182646.1 | cytoplasmic polyadenylation element binding protein 2(CPEB2) |
| **CPEB3** | NM_014912.3 | cytoplasmic polyadenylation element binding protein 3(CPEB3) |
| **CPM** | NM_001874.3 | carboxypeptidase M(CPM) |
| **CPSF4** | NM_001081559.1 | cleavage and polyadenylation specific factor 4(CPSF4) |
| **CRBN** | NM_016302.2 | cereblon(CRBN) |
| **CTHRC1** | NM_138455.2 | collagen triple helix repeat containing 1(CTHRC1) |
| **CTSC** | NM_148170.2 | cathepsin C(CTSC) |
| **CXorf36** | NM_024689.1 | chromosome X open reading frame 36(CXorf36) |
| **CYB561D1** | NM_001134404.1 | cytochrome b561 family member D1(CYB561D1) |
| **CYTH3** | NM_004227.3 | cytohesin 3(CYTH3) |
| **DCAF15** | NM_138353.2 | DDB1 and CUL4 associated factor 15(DCAF15) |
| **DCLRE1B** | NM_022836.2 | DNA cross-link repair 1B(DCLRE1B) |
| **DCUN1D2** | NM_001014283.1 | defective in cullin neddylation 1 domain containing 2(DCUN1D2) |
| **DCUN1D3** | NM_173475.1 | defective in cullin neddylation 1 domain containing 3(DCUN1D3) |
| **DDTL** | NM_001084393.1 | D-dopachrome tautomerase-like(DDTL) |
| **DDX19A** | NM_018332.3 | DEAD-box helicase 19A(DDX19A) |
| **DDX19B** | NM_001014451.1 | DEAD-box helicase 19B(DDX19B) |
| **DKK3** | NM_015881.5 | dickkopf WNT signaling pathway inhibitor 3(DKK3) |
| **DLST** | NM_001933.3 | dihydrolipoamide S-succinyltransferase(DLST) |
| **DNA2** | NM_001080449.1 | DNA replication helicase/nuclease 2(DNA2) |
| **DNAJA2** | NM_005880.2 | DnaJ heat shock protein family (Hsp40) member A2(DNAJA2) |
| **DNAL1** | NM_031427.1 | dynein axonemal light chain 1(DNAL1) |
| **DPH3** | NM_206831.1 | diphthamide biosynthesis 3(DPH3) |
| **DYRK2** | NM_006482.2 | dual specificity tyrosine phosphorylation regulated kinase 2(DYRK2) |
| **E2F2** | NM_004091.2 | E2F transcription factor 2(E2F2) |
| **E2F6** | NM_198256.2 | E2F transcription factor 6(E2F6) |
| **EFHD2** | NM_024329.4 | EF-hand domain family member D2(EFHD2) |
| **EGLN2** | NM_053046.2 | egl-9 family hypoxia inducible factor 2(EGLN2) |
| **EIF4G2** | NM_001418.3 | eukaryotic translation initiation factor 4 gamma 2(EIF4G2) |
| **ELK4** | NM_001973.2 | ELK4, ETS transcription factor(ELK4) |
| **ENTPD7** | NM_020354.2 | ectonucleoside triphosphate diphosphohydrolase 7(ENTPD7) |
| **ERGIC1** | NM_020462.1 | endoplasmic reticulum-golgi intermediate compartment 1(ERGIC1) |
| **ESPL1** | NM_012291.4 | extra spindle pole bodies like 1, separase(ESPL1) |
| **EZH2** | NM_004456.3 | enhancer of zeste 2 polycomb repressive complex 2 subunit(EZH2) |
| **FAM103A1** | NM_031452.2 | family with sequence similarity 103 member A1(FAM103A1) |
| **FAM104A** | NM_032837.1 | family with sequence similarity 104 member A(FAM104A) |
| **FAM118A** | NM_017911.1 | family with sequence similarity 118 member A(FAM118A) |
| **FAM135A** | NM_020819.2 | family with sequence similarity 135 member A(FAM135A) |
| **FARP1** | NM_005766.2 | FERM, ARH/RhoGEF and pleckstrin domain protein 1(FARP1) |
| **FAS** | NM_152872.1 | Fas cell surface death receptor(FAS) |
| **FBXO45** | NM_001105573.1 | F-box protein 45(FBXO45) |
| **FGD6** | NM_018351.2 | FYVE, RhoGEF and PH domain containing 6(FGD6) |
| **FGF5** | NM_004464.3 | fibroblast growth factor 5(FGF5) |
| **FIGNL2** | NM_001013690.2 | fidgetin like 2(FIGNL2) |
| **FKBP10** | NM_021939.2 | FK506 binding protein 10(FKBP10) |
| **FMO4** | NM_002022.1 | flavin containing monooxygenase 4(FMO4) |
| **FNDC3A** | NM_001079673.1 | fibronectin type III domain containing 3A(FNDC3A) |
| **FNDC3B** | NM_001135095.1 | fibronectin type III domain containing 3B(FNDC3B) |
| **FNIP1** | NM_001008738.2 | folliculin interacting protein 1(FNIP1) |
| **FOXN3** | NM_001085471.1 | forkhead box N3(FOXN3) |
| **FOXP1** | NM_032682.4 | forkhead box P1(FOXP1) |
| **FRAS1** | NM_206841.1 | Fraser extracellular matrix complex subunit 1(FRAS1) |
| **FRS2** | NM_006654.3 | fibroblast growth factor receptor substrate 2(FRS2) |
| **FZD4** | NM_012193.2 | frizzled class receptor 4(FZD4) |
| **GAB2** | NM_080491.1 | GRB2 associated binding protein 2(GAB2) |
| **GABPA** | NM_002040.2 | GA binding protein transcription factor alpha subunit(GABPA) |
| **GALNT1** | NM_020474.2 | polypeptide N-acetylgalactosaminyltransferase 1(GALNT1) |
| **GALNT4** | NM_003774.3 | polypeptide N-acetylgalactosaminyltransferase 4(GALNT4) |
| **GAN** | NM_022041.2 | gigaxonin(GAN) |
| **GAS7** | NM_201432.1 | growth arrest specific 7(GAS7) |
| **GDAP2** | NM_017686.2 | ganglioside induced differentiation associated protein 2(GDAP2) |
| **GDPD1** | NM_182569.2 | glycerophosphodiester phosphodiesterase domain containing 1(GDPD1) |
| **GFOD1** | NM_018988.2 | glucose-fructose oxidoreductase domain containing 1(GFOD1) |
| **GHR** | NM_000163.2 | growth hormone receptor(GHR) |
| **GJC1** | NM_001080383.1 | gap junction protein gamma 1(GJC1) |
| **GK5** | NM_001039547.1 | glycerol kinase 5 (putative)(GK5) |
| **GNPTAB** | NM_024312.3 | N-acetylglucosamine-1-phosphate transferase alpha and beta subunits(GNPTAB) |
| **GNS** | NM_002076.2 | glucosamine (N-acetyl)-6-sulfatase(GNS) |
| **GOLGA7** | NM_001002296.1 | golgin A7(GOLGA7) |
| **GOLT1B** | NM_016072.3 | golgi transport 1B(GOLT1B) |
| **GOPC** | NM_020399.2 | golgi associated PDZ and coiled-coil motif containing(GOPC) |
| **GPR156** | NM_153002.1 | G protein-coupled receptor 156(GPR156) |
| **GRPEL2** | NM_152407.3 | GrpE like 2, mitochondrial(GRPEL2) |
| **GXYLT1** | NM_001099650.1 | glucoside xylosyltransferase 1(GXYLT1) |
| **H2AFV** | NM_138635.3 | H2A histone family member V(H2AFV) |
| **HABP4** | NM_014282.1 | hyaluronan binding protein 4(HABP4) |
| **HAS2** | NM_005328.1 | hyaluronan synthase 2(HAS2) |
| **HBEGF** | NM_001945.1 | heparin binding EGF like growth factor(HBEGF) |
| **HELZ** | NM_014877.3 | helicase with zinc finger(HELZ) |
| **HIC2** | NM_015094.2 | HIC ZBTB transcriptional repressor 2(HIC2) |
| **HIF3A** | NM_022462.3 | hypoxia inducible factor 3 alpha subunit(HIF3A) |
| **HIPK2** | NM_022740.2 | homeodomain interacting protein kinase 2(HIPK2) |
| **HK2** | NM_000189.4 | hexokinase 2(HK2) |
| **ICMT** | NM_012405.3 | isoprenylcysteine carboxyl methyltransferase(ICMT) |
| **IDH2** | NM_002168.2 | isocitrate dehydrogenase (NADP(+)) 2, mitochondrial(IDH2) |
| **IGF2BP3** | NM_006547.2 | insulin like growth factor 2 mRNA binding protein 3(IGF2BP3) |
| **IKBKAP** | NM_003640.2 | inhibitor of kappa light polypeptide gene enhancer in B-cells, kinase complex-associated protein(IKBKAP) |
| **IKBKE** | NM_014002.2 | inhibitor of kappa light polypeptide gene enhancer in B-cells, kinase epsilon(IKBKE) |
| **INTS2** | NM_020748.1 | integrator complex subunit 2(INTS2) |
| **IPO9** | NM_018085.4 | importin 9(IPO9) |
| **KATNAL1** | NM_001014380.1 | katanin catalytic subunit A1 like 1(KATNAL1) |
| **KCTD10** | NM_031954.3 | potassium channel tetramerization domain containing 10(KCTD10) |
| **KCTD17** | NM_024681.1 | potassium channel tetramerization domain containing 17(KCTD17) |
| **KCTD21** | NM_001029859.1 | potassium channel tetramerization domain containing 21(KCTD21) |
| **KHNYN** | NM_015299.2 | KH and NYN domain containing(KHNYN) |
| **KIAA0895L** | NM_001040715.1 | KIAA0895 like(KIAA0895L) |
| **KIFC2** | NM_145754.2 | kinesin family member C2(KIFC2) |
| **KLF9** | NM_001206.2 | Kruppel like factor 9(KLF9) |
| **KLHDC8B** | NM_173546.1 | kelch domain containing 8B(KLHDC8B) |
| **KLHL24** | NM_017644.3 | kelch like family member 24(KLHL24) |
| **L2HGDH** | NM_024884.1 | L-2-hydroxyglutarate dehydrogenase(L2HGDH) |
| **LAMP2** | NM_013995.1 | lysosomal associated membrane protein 2(LAMP2) |
| **LBR** | NM_002296.2 | lamin B receptor(LBR) |
| **LEPROTL1** | NM_015344.1 | leptin receptor overlapping transcript-like 1(LEPROTL1) |
| **LGR4** | NM_018490.1 | leucine rich repeat containing G protein-coupled receptor 4(LGR4) |
| **LRFN4** | NM_024036.3 | leucine rich repeat and fibronectin type III domain containing 4(LRFN4) |
| **LRRC20** | NM_018239.2 | leucine rich repeat containing 20(LRRC20) |
| **LRRC8B** | NM_015350.1 | leucine rich repeat containing 8 family member B(LRRC8B) |
| **MAP3K3** | NM_203351.1 | mitogen-activated protein kinase kinase kinase 3(MAP3K3) |
| **MAPK11** | NM_002751.5 | mitogen-activated protein kinase 11(MAPK11) |
| **MAPK1IP1L** | NM_144578.3 | mitogen-activated protein kinase 1 interacting protein 1 like(MAPK1IP1L) |
| **MAPK6** | NM_002748.2 | mitogen-activated protein kinase 6(MAPK6) |
| **MAPK9** | NM_002752.3 | mitogen-activated protein kinase 9(MAPK9) |
| **MARS2** | NM_138395.2 | methionyl-tRNA synthetase 2, mitochondrial(MARS2) |
| **MBD2** | NM_015832.3 | methyl-CpG binding domain protein 2(MBD2) |
| **MBTPS2** | NM_015884.1 | membrane bound transcription factor peptidase, site 2(MBTPS2) |
| **MED28** | NM_025205.3 | mediator complex subunit 28(MED28) |
| **MED6** | NM_005466.2 | mediator complex subunit 6(MED6) |
| **MED8** | NM_201542.2 | mediator complex subunit 8(MED8) |
| **MESDC1** | NM_022566.1 | mesoderm development candidate 1(MESDC1) |
| **MFSD8** | NM_152778.1 | major facilitator superfamily domain containing 8(MFSD8) |
| **MGLL** | NM_007283.5 | monoglyceride lipase(MGLL) |
| **MTDH** | NM_178812.2 | metadherin(MTDH) |
| **MTPN** | NM_145808.2 | myotrophin(MTPN) |
| **MXD1** | NM_002357.2 | MAX dimerization protein 1(MXD1) |
| **NAPEPLD** | NM_198990.3 | N-acyl phosphatidylethanolamine phospholipase D(NAPEPLD) |
| **NDUFA4** | NM_002489.2 | NDUFA4, mitochondrial complex associated(NDUFA4) |
| **NEDD4L** | NM_015277.3 | neural precursor cell expressed, developmentally down-regulated 4-like, E3 ubiquitin protein ligase(NEDD4L) |
| **NEK3** | NM_152720.1 | NIMA related kinase 3(NEK3) |
| **NIPAL4** | NM_001099287.1 | NIPA like domain containing 4(NIPAL4) |
| **NLK** | NM_016231.4 | nemo like kinase(NLK) |
| **NME6** | NM_005793.3 | NME/NM23 nucleoside diphosphate kinase 6(NME6) |
| **NOVA1** | NM_002515.2 | NOVA alternative splicing regulator 1(NOVA1) |
| **NPEPL1** | NM_024663.3 | aminopeptidase-like 1(NPEPL1) |
| **NPHP3** | NM_153240.3 | nephrocystin 3(NPHP3) |
| **NRAS** | NM_002524.2 | neuroblastoma RAS viral oncogene homolog(NRAS) |
| **OLR1** | NM_002543.3 | oxidized low density lipoprotein receptor 1(OLR1) |
| **OPA3** | NM_025136.1 | optic atrophy 3 (autosomal recessive, with chorea and spastic paraplegia)(OPA3) |
| **OSMR** | NM_003999.1 | oncostatin M receptor(OSMR) |
| **PAG1** | NM_018440.3 | phosphoprotein membrane anchor with glycosphingolipid microdomains 1(PAG1) |
| **PARP8** | XM_943889.1 | poly(ADP-ribose) polymerase family member 8(PARP8) |
| **PARS2** | NM_152268.2 | prolyl-tRNA synthetase 2, mitochondrial (putative)(PARS2) |
| **PBX2** | NM_002586.4 | PBX homeobox 2(PBX2) |
| **PDE12** | NM_177966.4 | phosphodiesterase 12(PDE12) |
| **PDGFB** | NM_002608.1 | platelet derived growth factor subunit B(PDGFB) |
| **PGM2L1** | NM_173582.3 | phosphoglucomutase 2 like 1(PGM2L1) |
| **PGRMC1** | NM_006667.2 | progesterone receptor membrane component 1(PGRMC1) |
| **PHC3** | NM_024947.2 | polyhomeotic homolog 3(PHC3) |
| **PLA2G15** | NM_012320.3 | phospholipase A2 group XV(PLA2G15) |
| **PLAGL2** | NM_002657.2 | PLAG1 like zinc finger 2(PLAGL2) |
| **PLXND1** | NM_015103.1 | plexin D1(PLXND1) |
| **PM20D2** | NM_001010853.1 | peptidase M20 domain containing 2(PM20D2) |
| **POLR2D** | NM_004805.2 | RNA polymerase II subunit D(POLR2D) |
| **POU2F1** | NM_002697.2 | POU class 2 homeobox 1(POU2F1) |
| **PPP2R2A** | NM_002717.2 | protein phosphatase 2 regulatory subunit Balpha(PPP2R2A) |
| **PRDM1** | NM_001198.2 | PR/SET domain 1(PRDM1) |
| **PYGO2** | NM_138300.3 | pygopus family PHD finger 2(PYGO2) |
| **RAB15** | NM_198686.1 | RAB15, member RAS oncogene family(RAB15) |
| **RAB3GAP2** | NM_012414.3 | RAB3 GTPase activating non-catalytic protein subunit 2(RAB3GAP2) |
| **RAB40C** | NM_021168.2 | RAB40C, member RAS oncogene family(RAB40C) |
| **RAB8B** | NM_016530.2 | RAB8B, member RAS oncogene family(RAB8B) |
| **RALB** | NM_002881.2 | RAS like proto-oncogene B(RALB) |
| **RBM38** | NM_017495.4 | RNA binding motif protein 38(RBM38) |
| **RDX** | NM_002906.3 | radixin(RDX) |
| **RNF5** | NM_006913.2 | ring finger protein 5(RNF5) |
| **RNF7** | NM_183237.1 | ring finger protein 7(RNF7) |
| **RNF8** | NM_003958.2 | ring finger protein 8(RNF8) |
| **RPL36A** | NM_021029.3 | ribosomal protein L36a(RPL36A) |
| **RPUSD3** | NM_173659.2 | RNA pseudouridylate synthase domain containing 3(RPUSD3) |
| **RRM2** | NM_001034.1 | ribonucleotide reductase regulatory subunit M2(RRM2) |
| **RTKN** | NM_033046.2 | rhotekin(RTKN) |
| **RUFY2** | NM_017987.4 | RUN and FYVE domain containing 2(RUFY2) |
| **SAP30L** | NM_024632.4 | SAP30 like(SAP30L) |
| **SBNO1** | NM_018183.2 | strawberry notch homolog 1(SBNO1) |
| **SCARA3** | NM_182826.1 | scavenger receptor class A member 3(SCARA3) |
| **SCD** | NM_005063.4 | stearoyl-CoA desaturase(SCD) |
| **SEC14L1** | NM_003003.1 | SEC14 like lipid binding 1(SEC14L1) |
| **SEC24C** | NM_198597.1 | SEC24 homolog C, COPII coat complex component(SEC24C) |
| **SEC31B** | NM_015490.3 | SEC31 homolog B, COPII coat complex component(SEC31B) |
| **SEMA3F** | NM_004186.2 | semaphorin 3F(SEMA3F) |
| **SEMA4C** | NM_017789.3 | semaphorin 4C(SEMA4C) |
| **SERF2** | NM_001018108.2 | small EDRK-rich factor 2(SERF2) |
| **SESTD1** | NM_178123.3 | SEC14 and spectrin domain containing 1(SESTD1) |
| **SFT2D3** | NM_032740.3 | SFT2 domain containing 3(SFT2D3) |
| **SH2B3** | NM_005475.1 | SH2B adaptor protein 3(SH2B3) |
| **SH3RF3** | NM_001099289.1 | SH3 domain containing ring finger 3(SH3RF3) |
| **SIM2** | NM_005069.2 | single-minded family bHLH transcription factor 2(SIM2) |
| **SLC10A7** | NM_001029998.2 | solute carrier family 10 member 7(SLC10A7) |
| **SLC12A9** | NM_020246.2 | solute carrier family 12 member 9(SLC12A9) |
| **SLC25A24** | NM_213651.1 | solute carrier family 25 member 24(SLC25A24) |
| **SLC25A4** | NM_001151.2 | solute carrier family 25 member 4(SLC25A4) |
| **SLC2A12** | NM_145176.2 | solute carrier family 2 member 12(SLC2A12) |
| **SLC31A1** | NM_001859.2 | solute carrier family 31 member 1(SLC31A1) |
| **SLC31A2** | NM_001860.2 | solute carrier family 31 member 2(SLC31A2) |
| **SLC35D2** | NM_007001.1 | solute carrier family 35 member D2(SLC35D2) |
| **SLC37A4** | NM_001467.4 | solute carrier family 37 member 4(SLC37A4) |
| **SLC38A9** | NM_173514.1 | solute carrier family 38 member 9(SLC38A9) |
| **SLC4A4** | NM_003759.2 | solute carrier family 4 member 4(SLC4A4) |
| **SLC5A6** | NM_021095.1 | solute carrier family 5 member 6(SLC5A6) |
| **SLC6A15** | NM_182767.3 | solute carrier family 6 member 15(SLC6A15) |
| **SLC7A14** | NM_020949.1 | solute carrier family 7 member 14(SLC7A14) |
| **SLC9A9** | NM_173653.1 | solute carrier family 9 member A9(SLC9A9) |
| **SMAP2** | NM_022733.1 | small ArfGAP2(SMAP2) |
| **SMARCAD1** | NM_020159.2 | SWI/SNF-related, matrix-associated actin-dependent regulator of chromatin, subfamily a, containing DEAD/H box 1(SMARCAD1) |
| **SMUG1** | NM_014311.1 | single-strand-selective monofunctional uracil-DNA glycosylase 1(SMUG1) |
| **SNAP23** | NM_003825.2 | synaptosome associated protein 23(SNAP23) |
| **SNN** | NM_003498.4 | stannin(SNN) |
| **SPCS3** | NM_021928.1 | signal peptidase complex subunit 3(SPCS3) |
| **SPIRE1** | NM_020148.2 | spire type actin nucleation factor 1(SPIRE1) |
| **SRGAP1** | NM_020762.1 | SLIT-ROBO Rho GTPase activating protein 1(SRGAP1) |
| **SSH1** | NM_018984.2 | slingshot protein phosphatase 1(SSH1) |
| **ST3GAL1** | NM_003033.2 | ST3 beta-galactoside alpha-2,3-sialyltransferase 1(ST3GAL1) |
| **STARD3NL** | NM_032016.2 | STARD3 N-terminal like(STARD3NL) |
| **STK24** | NM_003576.3 | serine/threonine kinase 24(STK24) |
| **STK40** | NM_032017.1 | serine/threonine kinase 40(STK40) |
| **STX3** | NM_004177.3 | syntaxin 3(STX3) |
| **STXBP5** | NM_139244.2 | syntaxin binding protein 5(STXBP5) |
| **SULF1** | NM_015170.1 | sulfatase 1(SULF1) |
| **SURF4** | NM_033161.2 | surfeit 4(SURF4) |
| **SYNC** | NM_030786.2 | syncoilin, intermediate filament protein(SYNC) |
| **SYNCRIP** | NM_006372.3 | synaptotagmin binding cytoplasmic RNA interacting protein(SYNCRIP) |
| **SYNJ2BP** | NM_018373.1 | synaptojanin 2 binding protein(SYNJ2BP) |
| **SYT1** | NM_005639.1 | synaptotagmin 1(SYT1) |
| **TAF9B** | NM_015975.4 | TATA-box binding protein associated factor 9b(TAF9B) |
| **TAOK1** | NM_020791.1 | TAO kinase 1(TAOK1) |
| **TEAD3** | NM_003214.3 | TEA domain transcription factor 3(TEAD3) |
| **TEX261** | NM_144582.2 | testis expressed 261(TEX261) |
| **THBS1** | NM_003246.2 | thrombospondin 1(THBS1) |
| **TMED5** | NM_016040.3 | transmembrane p24 trafficking protein 5(TMED5) |
| **TMEM2** | NM_013390.1 | transmembrane protein 2(TMEM2) |
| **TMEM26** | NM_178505.5 | transmembrane protein 26(TMEM26) |
| **TMEM41A** | NM_080652.2 | transmembrane protein 41A(TMEM41A) |
| **TMOD2** | NM_014548.2 | tropomodulin 2(TMOD2) |
| **TNFAIP3** | NM_006290.2 | TNF alpha induced protein 3(TNFAIP3) |
| **TNFRSF1B** | NM_001066.2 | TNF receptor superfamily member 1B(TNFRSF1B) |
| **TNFSF10** | NM_003810.2 | tumor necrosis factor superfamily member 10(TNFSF10) |
| **TOR1AIP2** | NM_145034.2 | torsin 1A interacting protein 2(TOR1AIP2) |
| **TP53** | NM_000546.3 | tumor protein p53(TP53) |
| **TRABD** | NM_025204.2 | TraB domain containing(TRABD) |
| **TRAPPC1** | NM_021210.3 | trafficking protein particle complex 1(TRAPPC1) |
| **TRHDE** | NM_013381.1 | thyrotropin releasing hormone degrading enzyme(TRHDE) |
| **TRIB1** | NM_025195.2 | tribbles pseudokinase 1(TRIB1) |
| **TRIB2** | NM_021643.1 | tribbles pseudokinase 2(TRIB2) |
| **TRIM41** | NM_201627.1 | tripartite motif containing 41(TRIM41) |
| **TSC22D2** | NM_014779.2 | TSC22 domain family member 2(TSC22D2) |
| **TTC39C** | NM_153211.2 | tetratricopeptide repeat domain 39C(TTC39C) |
| **TTL** | NM_153712.4 | tubulin tyrosine ligase(TTL) |
| **TTLL4** | NM_014640.3 | tubulin tyrosine ligase like 4(TTLL4) |
| **TXLNA** | NM_175852.3 | taxilin alpha(TXLNA) |
| **UBE2G2** | NM_003343.4 | ubiquitin conjugating enzyme E2 G2(UBE2G2) |
| **UBN2** | NM_173569.2 | ubinuclein 2(UBN2) |
| **UBXN4** | NM_014607.3 | UBX domain protein 4(UBXN4) |
| **UCHL5** | NM_015984.2 | ubiquitin C-terminal hydrolase L5(UCHL5) |
| **UFM1** | NM_016617.1 | ubiquitin fold modifier 1(UFM1) |
| **UHRF1BP1** | NM_017754.3 | UHRF1 binding protein 1(UHRF1BP1) |
| **ULK2** | NM_014683.2 | unc-51 like autophagy activating kinase 2(ULK2) |
| **USP12** | NM_182488.1 | ubiquitin specific peptidase 12(USP12) |
| **USP38** | NM_032557.4 | ubiquitin specific peptidase 38(USP38) |
| **USP47** | NM_017944.3 | ubiquitin specific peptidase 47(USP47) |
| **UTP15** | NM_032175.2 | UTP15, small subunit processome component(UTP15) |
| **UTRN** | NM_007124.2 | utrophin(UTRN) |
| **VANGL2** | NM_020335.1 | VANGL planar cell polarity protein 2(VANGL2) |
| **VASH2** | NM_024749.2 | vasohibin 2(VASH2) |
| **VCPIP1** | NM_025054.3 | valosin containing protein interacting protein 1(VCPIP1) |
| **VGLL3** | NM_016206.2 | vestigial like family member 3(VGLL3) |
| **WARS2** | NM_201263.2 | tryptophanyl tRNA synthetase 2, mitochondrial(WARS2) |
| **WASL** | NM_003941.2 | Wiskott-Aldrich syndrome like(WASL) |
| **WDR37** | NM_014023.3 | WD repeat domain 37(WDR37) |
| **XRN1** | NM_019001.2 | 5'-3' exoribonuclease 1(XRN1) |
| **YAF2** | NM_001012424.1 | YY1 associated factor 2(YAF2) |
| **YOD1** | NM_018566.3 | YOD1 deubiquitinase(YOD1) |
| **YPEL2** | NM_001005404.3 | yippee like 2(YPEL2) |
| **YTHDF3** | NM_152758.4 | YTH N6-methyladenosine RNA binding protein 3(YTHDF3) |
| **ZBTB39** | NM_014830.1 | zinc finger and BTB domain containing 39(ZBTB39) |
| **ZBTB5** | NM_014872.1 | zinc finger and BTB domain containing 5(ZBTB5) |
| **ZCCHC3** | NM_033089.6 | zinc finger CCHC-type containing 3(ZCCHC3) |
| **ZCCHC9** | NM_032280.1 | zinc finger CCHC-type containing 9(ZCCHC9) |
| **ZMAT3** | NM_152240.1 | zinc finger matrin-type 3(ZMAT3) |
| **ZNF197** | NM_006991.3 | zinc finger protein 197(ZNF197) |
| **ZNF200** | NM_003454.2 | zinc finger protein 200(ZNF200) |
| **ZNF202** | NM_003455.2 | zinc finger protein 202(ZNF202) |
| **ZNF215** | NM_013250.1 | zinc finger protein 215(ZNF215) |
| **ZNF24** | NM_006965.1 | zinc finger protein 24(ZNF24) |
| **ZNF275** | NM_001080485.1 | zinc finger protein 275(ZNF275) |
| **ZNF280B** | NM_080764.2 | zinc finger protein 280B(ZNF280B) |
| **ZNF362** | NM_152493.2 | zinc finger protein 362(ZNF362) |
| **ZNF473** | NM_015428.1 | zinc finger protein 473(ZNF473) |
| **ZNF512B** | NM_020713.1 | zinc finger protein 512B(ZNF512B) |
| **ZNF583** | NM_152478.1 | zinc finger protein 583(ZNF583) |
| **ZNF689** | NM_138447.1 | zinc finger protein 689(ZNF689) |
| **ZNF697** | NM_001080470.1 | zinc finger protein 697(ZNF697) |

**Supplementary Table 5. Upregulated genes in HMCs treated with anti-dsDNA antibodies that contain a target binding site for miR-10a/b.**

| **ID** | **RefSeq** | **Gene Name** |
| --- | --- | --- |
| **AAK1** | NM_014911 | AP2 associated kinase 1 |
| **ABCC3** | NM_001144070 | ATP-binding cassette, sub-family C (CFTR/MRP), member 3 |
| **BCL6** | NM_001706 | B-cell CLL/lymphoma 6 |
| **DPF2** | NM_006268 | D4, zinc and double PHD fingers family 2 |
| **E2F3** | NM_001949 | E2F transcription factor 3 |
| **EFHC1** | NM_018100 | EF-hand domain (C-terminal) containing 1 |
| **EPHA10** | NM_173641 | EPH receptor A10 |
| **EPHA2** | NM_004431 | EPH receptor A2 |
| **EDEM1** | NM_014674 | ER degradation enhancer, mannosidase alpha-like 1 |
| **FBXO22** | NM_147188 | FBXO22 opposite strand (non-protein coding); F-box protein 22 |
| **INO80D** | NM_017759 | INO80 complex subunit D |
| **LYSMD3** | NM_198273 | LysM, putative peptidoglycan-binding, domain containing 3 |
| **NEK6** | NM_001145001 | NIMA (never in mitosis gene a)-related kinase 6 |
| **RB1CC1** | NM_014781 | RB1-inducible coiled-coil 1 |
| **RBMS3** | NM_001003793 | RNA binding motif, single stranded interacting protein |
| **SIX4** | NM_017420 | SIX homeobox 4 |
| **SMAD2** | NM_005901 | SMAD family member 2 |
| **SMURF1** | NM_181349 | SMAD specific E3 ubiquitin protein ligase 1 |
| **STARD13** | NM_178006 | StAR-related lipid transfer (START) domain containing 13 |
| **TIAM1** | NM_003253 | T-cell lymphoma invasion and metastasis 1 |
| **WNK3** | NM_001002838 | WNK lysine deficient protein kinase 3 |
| **ANK3** | NM_020987 | ankyrin 3, node of Ranvier (ankyrin G) |
| **ANKFY1** | NM_016376 | ankyrin repeat and FYVE domain containing 1 |
| **ANXA7** | NM_001156 | annexin A7 |
| **ARRDC3** | NM_020801 | arrestin domain containing 3 |
| **BCR** | NM_004327 | breakpoint cluster region |
| **BAZ2B** | NM_013450 | bromodomain adjacent to zinc finger domain, 2B |
| **CREB1** | NM_004379 | cAMP responsive element binding protein 1 |
| **CREBL2** | NM_001310 | cAMP responsive element binding protein-like 2 |
| **CAMK2G** | NM_001222 | calcium/calmodulin-dependent protein kinase II gamma |
| **CASK** | NM_003688 | calcium/calmodulin-dependent serine protein kinase (MAGUK family) |
| **CEP350** | NM_014810 | centrosomal protein 350 kDa |
| **DOCK11** | NM_144658 | dedicator of cytokinesis 11 |
| **DVL3** | NM_004423 | dishevelled, dsh homolog 3 (*Drosophila*) |
| **FLRT2** | NM_013231 | fibronectin leucine rich transmembrane protein 2 |
| **FXR2** | NM_004860 | fragile X mental retardation, autosomal homolog 2 |
| **GLS** | NM_014905 | Glutaminase |
| **IGDCC4** | NM_020962 | immunoglobulin superfamily, DCC subclass, member 4 |
| **IPO8** | NM_006390 | importin 8 |
| **ITPKC** | NM_025194 | inositol 1,4,5-trisphosphate 3-kinase C |
| **IFFO2** | NM_001136265 | intermediate filament family orphan 2 |
| **JARID2** | NM_004973 | jumonji, AT rich interactive domain 2 |
| **L3MBTL3** | NM_001007102 | l(3)mbt-like 3 (*Drosophila*) |
| **LPHN1** | NM_014921 | latrophilin 1 |
| **LRRFIP1** | NM_001137550 | leucine rich repeat (in FLII) interacting protein 1 |
| **LCOR** | NM_001170765 | ligand dependent nuclear receptor corepressor |
| **MED1** | NM_004774 | mediator complex subunit 1 |
| **MTF2** | NM_007358 | metal response element binding transcription factor 2 |
| **MAPKBP1** | NM_014994 | mitogen-activated protein kinase binding protein 1 |
| **MAP4K4** | NM_145687 | mitogen-activated protein kinase kinase kinase kinase 4 |
| **MTMR3** | NM_021090 | myotubularin related protein 3 |
| **NONO** | NM_007363 | non-POU domain containing, octamer-binding |
| **NFIX** | NM_002501 | nuclear factor I/X (CCAAT-binding transcription factor) |
| **NFAT5** | NM_006599 | nuclear factor of activated T-cells 5, tonicity-responsive |
| **NCOR2** | NM_006312 | nuclear receptor co-repressor 2 |
| **NCOA6** | NM_001318240 | nuclear receptor coactivator 6 |
| **NR2C2** | NM_001291694 | nuclear receptor subfamily 2, group C, member 2 |
| **NUP50** | NM_007172 | nucleoporin 50 kDa |
| **NACC1** | NM_052876 | nucleus accumbens associated 1, BEN and BTB (POZ) domain containing |
| **PISD** | NM_178022 | phosphatidylserine decarboxylase |
| **PDE4A** | NM_001243121 | phosphodiesterase 4A, cAMP-specific (phosphodiesterase E2 dunce homolog, *Drosophila*) |
| **PIK3CA** | NM_006218 | phosphoinositide-3-kinase, catalytic, alpha polypeptide |
| **PCBD2** | NM_032151 | pterin-4 alpha-carbinolamine dehydratase/dimerisation cofactor of hepatocyte nuclear factor 1 alpha (TCF1) 2 |
| **PURB** | NM_033224 | purine-rich element binding protein B |
| **RPRD1A** | NM_001303412 | regulation of nuclear pre-mRNA domain containing 1A |
| **RTN4R** | NM_023004 | reticulon 4 receptor |
| **SCARB2** | NM_005506 | scavenger receptor class B, member 2 |
| **SERPINE1** | NM_000602 | serpin peptidase inhibitor, clade E (nexin, plasminogen activator inhibitor type 1), member 1 |
| **SLC41A2** | NM_032148 | solute carrier family 41, member 2 |
| **SNX29** | NM_032167 | sorting nexin 29 |
| **SNX4** | NM_003794 | sorting nexin 4 |
| **SPAG9** | NM_001130528 | sperm associated antigen 9 |
| **STRN** | NM_003162 | striatin, calmodulin binding protein |
| **SDC1** | NM_002997 | syndecan 1 |
| **TMEM132B** | NM_052907 | transmembrane protein 132B; hypothetical LOC121296 |
| **TMEM183A** | NM_138391 | transmembrane protein 183A |
| **TMEM183B** | NM_052907 | transmembrane protein 183B |
| **UBE2I** | NM_194260 | ubiquitin-conjugating enzyme E2I (UBC9 homolog, yeast) |
| **WAPAL** | NM_015045 | wings apart-like homolog (*Drosophila*) |
| **ZNF608** | NM_020747 | zinc finger protein 608 |
|  |  |  |

**Supplementary Table 6. List of primer sequences**

| **Official gene name** | **Sequence primer** | **Amplicon Size (bp)** |
| --- | --- | --- |
| *GADPH* | Forward 5’-GCACCGTCAAGGCTGAGAAC-3’  Reverse 5’-ATGGTGGTGAAGACGCCAGT-3’ | 142 |
| *IL6* | Forward 5’-GGCACTGGCAGAAAACAACC-3’  Reverse 5’-GCAAGTCTCCTCATTGAATCC-3’ | 85 |
| *IL8* | Forward 5’-ACACTGCGCCAACACAGAAATTA-3’  Reverse 5’-TTTGCTTGAAGTTTCACTGGCATC-3’ | 185 |
| *IL1B* | Forward 5’-ACAGATGAAGTGCTCCTTCCA-3’  Reverse 5’-GTCGGAGATTCGTAGCTGGAT-3’ | 73 |
| *TNF* | Forward 5’-CTTCTCCTTCCTGATCGTGG-3’  Reverse 5’-GCTGGTTATCTCTCAGCTCCA-3’ | 266 |
| *MMP10* | Forward 5’-GGCTCTTTCACTCAGCCAAC-3’  Reverse 5’-TCCCGAAGGAACAGATTTTG-3’ | 176 |
| *CREB1* | Forward 5’-ACGAAAGCAGTGACGGAGG-3’  Reverse 5’-CGGTGGGAGCAGATGATGTT-3’ | 198 |
| *NFAT5* | Forward 5’-CAACAACATGACACTGGCGG-3’  Reverse 5’-TCGAAAAACCAATCTGGCACG-3’ | 124 |
| *PIK3CA* | Forward 5’-TCCAGACGCATTTCCACAGC-3’  Reverse 5’-GTCACATAAGGGTTCTCCTCCA-3’ | 186 |
| *SMAD2* | Forward 5’-CACAGCCCTCACTCACTGTA-3’  Reverse 5’-GCACTCAGCAAAAACTTCCCC-3’ | 170 |
| *MAP4K4* | Forward 5’-TGTTAAAACGGGTCAGTTGGC-3’  Reverse 5’-TGTCCTGGAGGGCTCTTTTTG-3’ | 159 |
| *MAP3K7* | Forward 5’-ACTTGATGCGGTACTTTC-3’  Reverse 5’-GGTTGCGGCGATCCTA-3’ | 350 |
| *KLF4* | Forward 5’-GGGCTGCGGCAAAACCTACACA-3’  Reverse 5’-CCATCCACAGCCGTCCCAGTCA-3’ | 103 |
| *HOXA1* | Forward 5’-CCAGGAGCTCAGGAAGAAGAGAT-3’  Reverse 5’-CCCTCTGAGGCATCTGATTGGGTTT-3’ | 247 |

Bp, base pairs.
